# Clinician–Artificial Intelligence Collaboration for Mediterranean Meal-Plan Generation: Development, Technical Feasibility, and Professional Acceptability of the MAI-DIET Framework

**DOI:** 10.3390/nu18162661

**Published:** 2026-08-14

**Authors:** Konstantinos Divanis, Alexandra Foscolou, Georgios Prosalentis, Charalampos Mylonas, Maria I. Antoniou, Athina Krokou, Antonios-Nikolaos Filias, Eleni Papagiannopoulou, Panagiotis D. Dousis, Aikaterini D. Polychronidou, Georgia Gkioxari, Aristea Gioxari

**Affiliations:** 1 Department of Nutritional Science and Dietetics, School of Health Sciences, University of the Peloponnese, Antikalamos, 24100 Kalamata, Greece; nds22026@go.uop.gr (K.D.); alexandra.foscolou@go.uop.gr (A.F.); nds22087@go.uop.gr (G.P.); nds22067@go.uop.gr (C.M.); nds23030@go.uop.gr (M.I.A.); ds24048@go.uop.gr (A.K.); nds21136@go.uop.gr (A.-N.F.); nds23158@go.uop.gr (E.P.); nds22027@go.uop.gr (P.D.D.); nds22125@go.uop.gr (A.D.P.); 2 Division of Computing and Mathematical Sciences, Caltech, Pasadena, CA 91125, USA

**Keywords:** Mediterranean diet, AI-assisted meal-plan generation, recipe-based dietary planning, clinician-supervised artificial intelligence, nutrient-calculation pipeline, healthcare-professional acceptability

## Abstract

Background/Objectives: Adherence to the Mediterranean diet has declined in recent decades, highlighting the need for practical, technology-enabled tools to support its adoption. This study developed and evaluated MAI-DIET, a clinician-supervised, data-driven, AI-assisted programmatic framework designed to generate Greek–Mediterranean recipe-based meal plans for apparently healthy community-dwelling adults. Methods: MAI-DIET is combined with standardized food-composition and recipe libraries and a rule-based module that performs meal-plan generation and nutrient calculations. Claude Opus 4.7 supported predefined operator-supervised transformation tasks, and provided the interface for launching the rule-based module. Six 28-day recipe-based dietary plans were generated for hypothetical adults with energy goals ranging from 1600 to 2600 kcal/day. The generated plans were not tested in the intended population. For each plan, detailed nutritional analysis was performed according to predefined criteria. The quality of the dietary plans was assessed using validated scores, i.e., MedDietScore, dietary phytochemical index (DPI), and GR-UPFAST. Early-stage acceptability was evaluated by 103 healthcare professionals using a five-point Likert questionnaire. Results: All plans met the predefined energy criteria, while most nutrient targets were achieved. Deviations were observed for sodium, particularly in the higher-energy plans (reaching +36.3%), and for calcium, which was 17.5% below the EFSA population reference intake in the 1600 kcal/day plan. MedDietScore ranged between 35 and 36/55, while DPI was 47.0–50.9% and GR-UPFAST was 2.0–4.5/70. Overall acceptability was favorable (3.84 ± 0.59), with Cronbach’s alpha values of 0.838–0.952. Conclusions: The findings support the technical feasibility of the MAI-DIET framework and its preliminary acceptability among healthcare professionals. Further evaluation is required before conclusions can be drawn regarding its practical effectiveness.

## 1. Introduction

The Mediterranean dietary pattern, characterized by the high consumption of fruits, vegetables, legumes, whole grains, olive oil, nuts, and fish, with a moderate intake of dairy and low intake of red and processed meats, is widely recognized as a health-promoting dietary model [1]. Substantial evidence has linked greater adherence to this pattern with a reduced risk of noncommunicable diseases, particularly cardiometabolic disorders; improved health-related quality of life; and healthy aging [1,2]. Adherence to the Mediterranean dietary pattern has been associated with reduced all-cause and cardiovascular mortality [3,4]. In the PREDIMED randomized-controlled trial, which included 7447 participants, adherence to the Mediterranean diet supplemented with extra-virgin olive oil or nuts was associated with a nearly 30% reduction in major cardiovascular events [5]. In line with these findings, the CORDIOPREV randomized-controlled trial, which included 1002 patients with coronary heart disease, showed that a Mediterranean dietary pattern provided greater protection against recurrent major cardiovascular events than a low-fat diet [6].

Nevertheless, adherence to the Mediterranean diet has declined over recent decades across the Mediterranean basin, largely reflecting a nutrition transition toward more Westernized dietary patterns characterized by the higher consumption of ultra-processed foods, refined grains, red and processed meat, sweets, and saturated fats [7,8]. In Greece, historically regarded as one of the heartlands of the Mediterranean diet, adherence to this dietary pattern is declining, a trend that may contribute to the growing cardiometabolic health burden [9]. In the ATTICA prospective cohort study, about half of participants moved from high to low adherence between 2002 and 2012, while 30% of cardiovascular disease, type 2 diabetes, hypertension, and hypercholesterolemia cases were prevented following high long-term adherence to the Mediterranean diet [9]. This decline highlights the need for practical tools that can effectively translate Mediterranean diet evidence into actionable meal plans for community-dwelling populations.

Digital tools may support public health nutrition and clinical practice by helping healthcare professionals promote healthier eating habits and support dietary behavior change [10,11,12]. More recently, artificial intelligence (AI), particularly large language models (LLMs), has been used to generate dietary plans, with some outputs showing moderate adherence to clinical guidelines [13]. However, these outputs require expert oversight and validated nutrient-calculation methods, because LLMs may have limitations in reasoning, personalization, and numerical accuracy [13,14,15,16,17,18]. Evidence from AI-generated dietary plans suggests that energy and nutrient calculations may sometimes be inaccurate or inconsistent, supporting the need for structured calculation procedures linked to validated food-composition data [19,20,21]. Recent evidence has indicated that LLMs may be better suited to predefined transformation tasks, such as summarization, patient communication, recipe-data structuring, and presentation of recommendations generated by rule-based nutrition engines, rather than direct nutrient calculation or autonomous meal planning [22].

To address these challenges, the MAI-DIET framework was designed to translate Greek–Mediterranean diet evidence into practical, recipe-based meal-planning outputs for apparently healthy community-dwelling adults. The framework combined standardized food-composition and recipe libraries, a rule-based nutrient-calculation and meal-planning module, and human review. The LLM supported predefined, operator-supervised transformation tasks, including recipe-data structuring and library review, and served as the interface for launching the rule-based computational module responsible for meal-plan generation. The LLM itself performed no meal-plan generation or nutrient calculations.

Therefore, the present study aimed to: (i) develop the MAI-DIET framework; (ii) evaluate the nutrient adequacy and overall quality of its generated meal plans using predefined nutrient criteria and established diet-quality indices; (iii) compare their structural consistency with plans generated directly by an LLM; and (iv) assess early-stage acceptability by practicing healthcare professionals.

## 2. Materials and Methods

### 2.1. Study Design

This study used a cross-sectional, early-stage acceptability design comprising three sequential components: (A) the MAI-DIET framework, which was developed to generate Greek–Mediterranean recipe-based dietary plans for adults, combining standardized food-composition and recipe libraries, a rule-based nutrient-calculation and meal-planning module, and human review; (B) the nutritional quality of the generated outputs, which was evaluated by applying established and validated indices of Mediterranean diet adherence and overall diet quality to six 28-day meal plans generated for six hypothetical individuals; and (C) the professional acceptability of these outputs, which was assessed through ratings provided by practicing healthcare professionals.

### 2.2. The MAI-DIET Workflow

**Figure 1:**
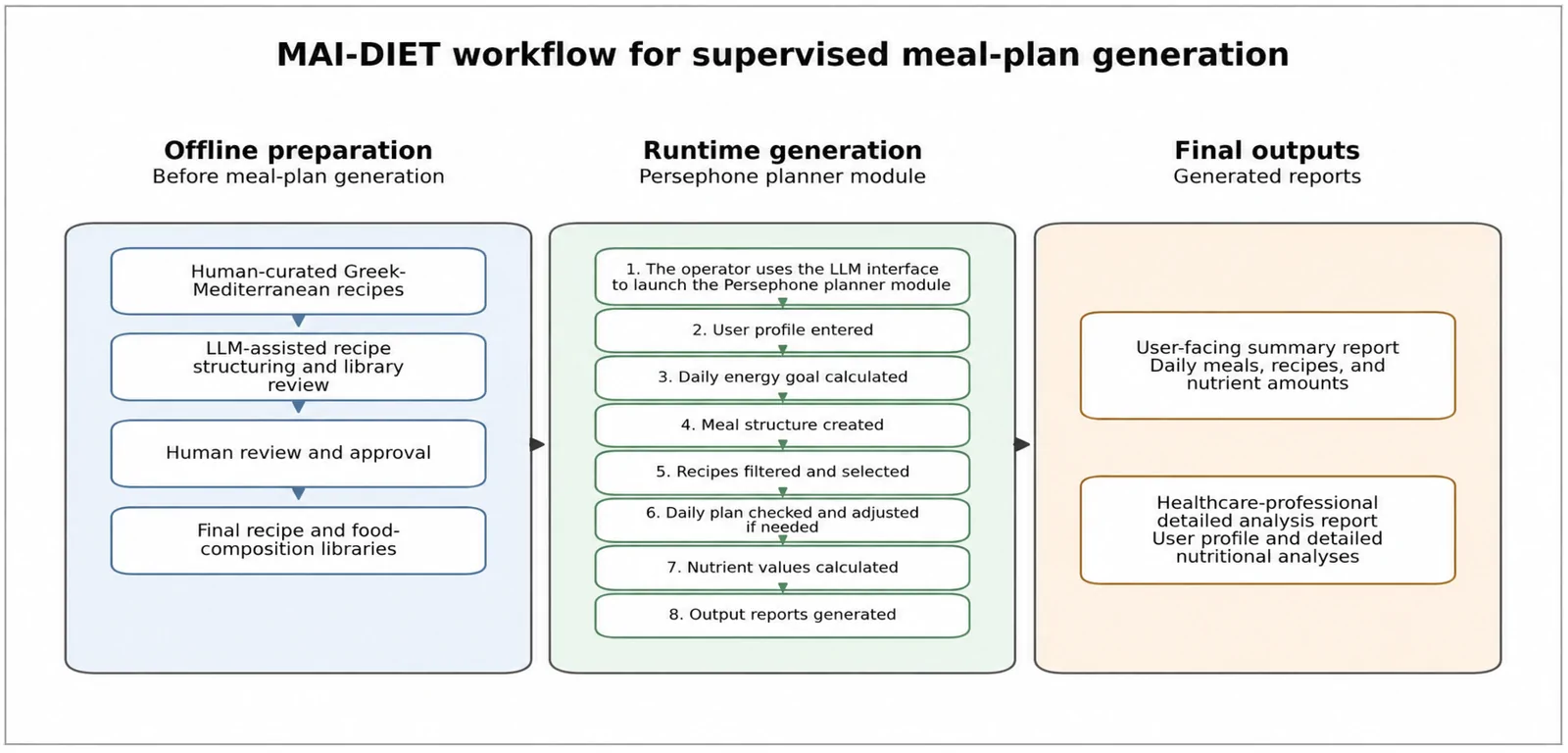
MAI-DIET workflow for supervised meal-plan generation. The figure shows the offline preparation stage, the runtime meal-plan generation stage, and the final output reports. LLM-assisted tasks were performed under operator supervision and reviewed by humans before use. At runtime, all computation was performed by the “Persephone” planner module developed by our team. The module calculated energy needs, selected recipes, checked daily plans, calculated nutrient values, and generated reports for users and healthcare professionals. The supervising operator launched the module through an LLM session that served solely as an execution interface; the LLM performed no calculations or recipe selection.

The MAI-DIET framework consisted of the following interacting components: (A) Claude Opus 4.7 [23] supported predefined data-transformation tasks during system development and served as the operator-facing interface for initiating the planning process, under explicit operator supervision. Once the planning process was launched, the LLM had no role in runtime meal-plan generation and performed no calculations of energy requirements, nutrient values, macronutrient distributions, scoring results, or ingredient-level nutrient totals. (B) A rule-based computational module was developed by the research team, hereafter referred to as the “Persephone” planner module, which performed all computational and meal-plan generation steps, from processing user inputs to exporting the final documents. (C) A human-review component, through which the principal investigators reviewed the recipe and food-composition libraries and verified ingredient-mapping decisions. Figure 1 summarizes the MAI-DIET workflow, including the offline preparation stage, the runtime meal-plan generation stage, and the final output reports.

### 2.3. MAI-DIET Architecture

**Figure 2:**
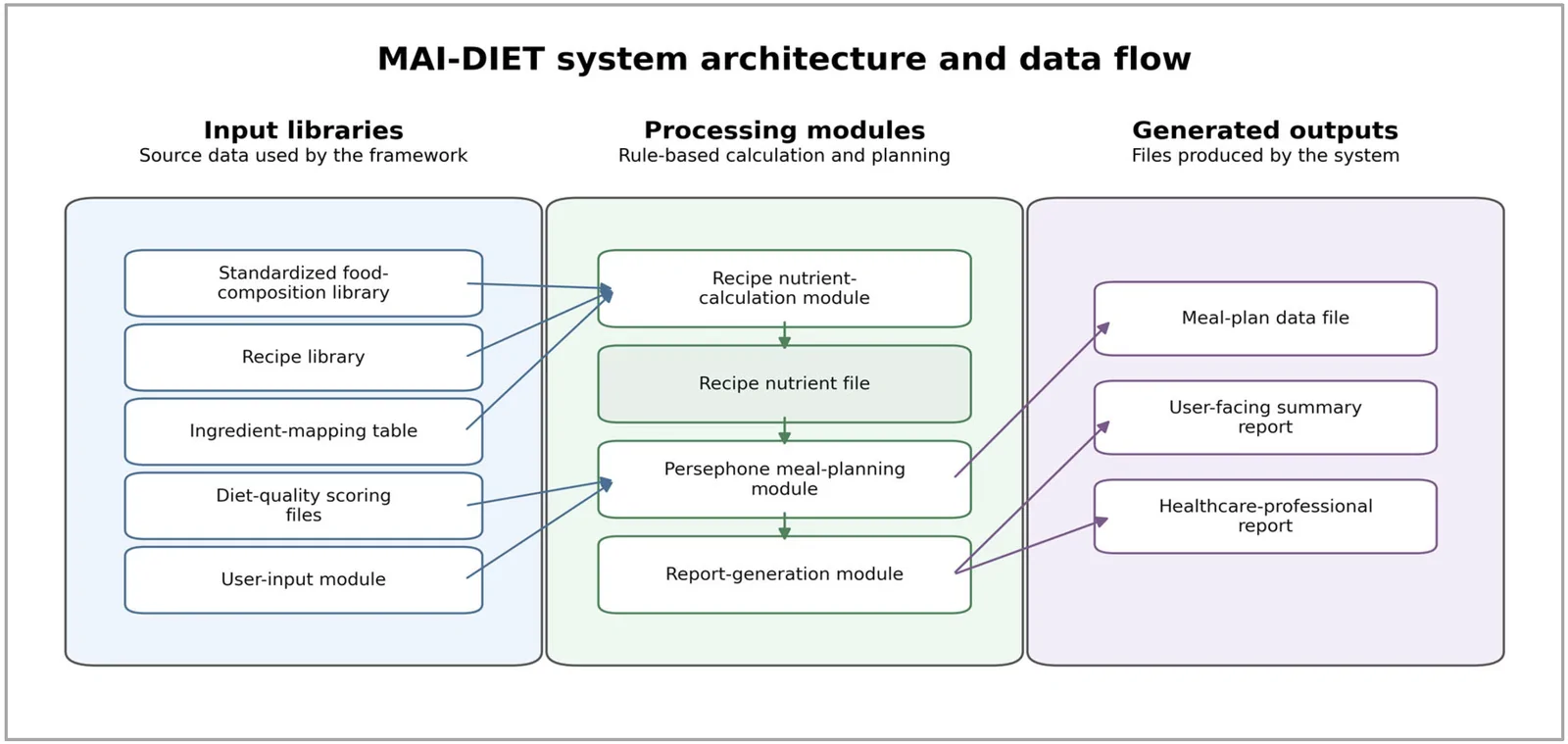
MAI-DIET software architecture and data flow. The MAI-DIET framework was implemented as a set of interconnected Python modules and library files (Python version 3.14.0; Python Software Foundation, Wilmington, DE, USA). The input libraries included the standardized food-composition library, recipe library, ingredient-mapping table, diet-quality scoring files, and user-input module. These inputs were processed by the recipe nutrient-calculation module, the “Persephone” meal-planning module, and the report-generation module. The recipe nutrient file served as the central reference file (green color box) for the nutrient values used during meal-plan generation and report production. This ensured that the nutrient values used during recipe selection were consistent with those presented in the exported reports.

The MAI-DIET framework was implemented as a set of interconnected Python modules and library files (Figure 2). Python version 3.14.0 (Python Software Foundation, Wilmington, DE, USA) was used as the rule-based computational environment for the MAI-DIET framework. The Python modules imported the standardized recipe and food-composition libraries, linked recipe ingredients to their matched food-composition entries, applied cooking-retention factors where required, and calculated recipe-level energy and nutrient values. The same Python workflow was then used by the “Persephone” planner module to process user inputs, estimate energy targets, scale recipe portions, filter and select recipes, check daily energy and nutrient tolerance criteria, and generate the final output reports.

Thus, all numerical calculations and meal-plan generation steps were performed by Python-based rule-defined procedures, while the LLM supported predefined transformation tasks under operator-supplied constraints. The LLM worked within rules, controlled vocabularies, or predefined decision limits supplied by the operator (Table 1). For meal-plan generation, the LLM served only as the interface through which the operator launched the deterministic “Persephone” planning module; all subsequent computations were performed by the module independently of the LLM. The framework contained no retrieval-augmented generation component or associated retrieval infrastructure, such as embedding models or vector databases. All LLM-assisted tasks listed in Table 1 were performed using Claude Opus 4.7 through the provider’s standard interactive interface between 16 and 30 April 2026. The interface did not provide access to generation parameters, such as temperature, top-p, or maximum token limits; therefore, these parameters were set to default values.

The LLM proposed hypothetical anthropometric inputs and scale-factor overrides, which were processed by the deterministic module and saved only after operator approval. During development, the human operator used the LLM interface to instruct Persephone to generate candidate plans with alternative seed sequences. The operator then selected the sequence that produced the least recipe repetition over the 28-day period. Selection was based solely on recipe variety, while nutrient outputs were not examined. The selected seed sequence was subsequently applied unchanged to all six evaluated plans (Table 1).

The LLM did not generate recipes, propose new dishes, or write free-text culinary content; all recipes were human-curated, as described above. The role of the LLM was limited to rule-based transformation of this material under human supervision. This combination of human-defined rules, supervised review, and constrained LLM-assisted transformation reflects a distinctive methodological characteristic of the MAI-DIET framework. Every LLM contribution was recorded in version-controlled records. These records were reviewable by an experienced AI-engineer (G.G.) at any stage of the system development or plan production. Bit-level reproducibility was achieved only for the deterministic-substrate operations (the “Persephone” planner module). LLM-attributed decisions were auditable but not bit-replayable across LLM sessions.

### 2.4. Database Development

The MAI-DIET’s development involved creating food and recipe libraries reflecting Greek–Mediterranean dietary practice. Together, these libraries provided the basis for generating recipe-based meal plans and calculating their nutritional content.

Candidate recipes were identified by experienced dietitians through targeted searches of Greek and Mediterranean culinary sources, including academic publications, established Greek dietary websites, traditional cookbooks, and traditional family recipes documented by the investigators. For each recipe, key culinary characteristics were recorded to create a structured entry in the production database. These included the dish name (e.g., traditional Greek moussaka), meal category (breakfast, lunch, dinner, or snack), main ingredient or principal food component (e.g., spinach in traditional Greek spinach pie), ingredient quantities in grams or milliliters (including foods, seasonings, spices, and herbs), number of servings, cooking method (e.g., none, boiled, simmered, grilled, baked, sautéed, fried), preparation time, and preparation instructions.

Recipe information was converted into the structured database using an extract–transform–load (ETL) process. The original recipe information served as the input, and the output was a standardized database entry. The transformation rules included converting non-metric household measures into grams or milliliters where needed, standardizing ingredient names, and classifying cooking methods into predefined categories to support consistent nutrient-retention calculations. During this phase, the LLM was used only to support the structured transformation process. Each transformed recipe entry was reviewed by the principal dietitians (A.G. and A.F.) before inclusion in the production database. The final recipe library included 239 recipes, which were decomposed into 1577 individual ingredient entries.

For each ingredient included in the recipe library, a standardized food-composition entry was created providing per-100 g nutrient values across all nutrient and metadata fields. Nutrient values were obtained from public structured-data resources, namely the United States Department of Agriculture FoodData Central database [27] and the OpenNutrition Model Context Protocol nutrient server [28]. Each candidate ingredient was systematically searched in both reference food-composition databases. For each ingredient, the quality of the match with the reference food-composition databases was recorded and later used in the provenance-quality scoring process. The final standardized food-composition library included 535 food entries: 491 matched to both FoodData Central and OpenNutrition, 30 matched only to OpenNutrition, and 14 matched only to FoodData Central. Of these 535 entries, 229 are directly referenced by the recipe library. No food entry was generated by the AI system. Approximately 1700 ingredient name candidates from the recipe library were resolved to standardized food entries through a mapping table. Each entry in the table records a match tier (exact or contained) and a match confidence, and all entries were reviewed by the principal investigators (G.G., A.G., A.F.) before meal-plan generation.

Herein, tomato was used as an example, showing how it was identified in the original recipe, matched to a standardized food-composition entry, adjusted for cooking where applicable, and included in the final recipe-level nutrient calculation. The example recipe was “Green Bean and Potato Olive Oil Stew,” a traditional Greek dish characterized by long simmering and a substantial amount of fresh tomato (Table 2).

### 2.5. Energy and Nutrient Computation

The system converted user demographic inputs into a daily kilocalorie target through a series of deterministic steps:

A. Basal metabolic rate (BMR), expressed as kcal/day, was estimated using a predefined equation-selection rule (Supplementary File S1 Table S1). For individuals with a BMI ≥ 30 kg/m^2^, the Mifflin–St Jeor equation was applied [29]. For adults aged 65 years or older with a BMI below 30 kg/m^2^, the Henry (2005) [30] equations were applied. For the remaining adults—those with a BMI below 30 kg/m^2^ and age below 65 years—the Harris–Benedict Revised equations were applied [31,32]. All six evaluated personas fell in the Harris–Benedict Revised cohort (ages 28–35 years, BMI 20.8–26.6 kg/m^2^).
B. Total daily energy expenditure (TEE), expressed as kcal/day, was estimated by multiplying the BMR by the relevant physical activity level coefficient [26].
C. The daily energy goal, expressed as kcal/day, was derived from total energy expenditure (TEE) and adjusted according to the weight goal selected by the user. For a weight-maintenance goal, the energy goal was set equal to TEE. For a weight-loss goal an energy deficit was applied up to 500 kcal/day in compliance with the 2013 AHA/ACC/TOS Guidelines for the Management of Overweight and Obesity in Adults [33]. For a weight-gain goal, a corresponding energy surplus was applied by up to 500 kcal/day. The adjusted energy goal was subject to a predefined lower bound of 1000 kcal/day, consistent with the lower limit of the range described for low-calorie diets [34]; the upper bound was set at 110% of the individual’s TEE, corresponding to a 10% energy surplus and allowing the target to scale with estimated energy requirements, including those of highly active adults [35].
D. Macronutrient criteria were set as 45–55% of energy from carbohydrates, 15–20% from protein, and 30–40% from fat. A monounsaturated (MUFA)-dominant fat profile was specified to reflect the Mediterranean dietary pattern [36,37]. Micronutrient targets for vitamin C, vitamin B12, folate, iron, calcium, fiber, and marine n-3 fatty acids were based on sex-specific EFSA population reference intakes or adequate intakes [38]. The sodium target was based on the WHO recommendation of <2000 mg/day and the AHA threshold of ≤2300 mg/day [39,40].
E. Recipe portions were scaled according to the user’s daily energy goal. A scaling factor was calculated by dividing the user’s energy goal by 2000 kcal/day, which was used as the reference energy level. For example, a goal of 1600 kcal/day produced a scaling factor of 0.80, whereas a goal of 2400 kcal/day produced a scaling factor of 1.20. Because recipe-based meal plans could not always match the exact energy goal, predefined tolerance ranges based on the scaling factor were used to determine whether each daily plan was acceptable. This approach is consistent with evidence that portion size affects total energy intake [34].

### 2.6. Meal-Structure Generation, Recipe Filtering, and Recipe Selection

For each meal plan, the system created a daily meal structure based on the number of days in the plan and the selected number of snacks. The user’s daily calorie goal was then distributed across meals using a prespecified energy-allocation rule—18% for breakfast, 32% for lunch, 29% for dinner, and 10.5% for each of the two snacks (21% combined)—to allow for the standardization of energy distribution consistent with the evidence that meal timing and frequency are associated with cardiometabolic health [41]. Candidate recipes were then filtered in a fixed sequence according to reported allergens, predefined nutrition criteria (i.e., energy, carbohydrate, protein, fat, MUFA, marine n-3, vitamin C, vitamin B12, folate, iron, calcium, fiber, and sodium), preparation times, and nutrient-data confidence levels. After filtering, the system selected one recipe for each day and each meal occasion using a predefined scoring rule (Supplementary File S1 Table S2). The selected recipe was the one that best matched the assigned energy goal, avoided excessive repetition of similar recipes, supported preferred food-group choices, and remained within predefined fat-content limits. Lower scores indicated better suitability for the assigned meal or snack.

Food-group frequency limits were applied to support variety and alignment with the Greek National Dietary Guide for Adults [36]. Additional limits were used to avoid frequent repetition of the same recipes across the 28-day meal plan. After each daily plan was generated, the system checked whether the total daily energy and fat percentage were within the predefined acceptable range. If they were not, the system replaced one eligible meal and recalculated the daily plan for at most two passes per check. Because the system followed fixed rules and applied a fixed seed sequence, using identical inputs and the same recipe library produced identical 28-day plans.

The deterministic “Persephone” planner used a greedy sequential heuristic with hard-constraint filtering, weighted scoring, and bounded local repair. It did not perform global optimization, constraint programming, backtracking, or metaheuristic search.

Constraint filtering and relaxation: For each meal slot, recipes that violated predefined constraints on repetition, meal variety, food-category frequency, or projected fat intake were excluded. If no candidate met the energy tolerance, the band was progressively widened while all structural constraints remained enforced.

Weighted scoring: Eligible recipes were ranked according to energy-target proximity, previous use, food-group balance, projected fat contribution, and a random tie-break. The lowest-scoring candidate was selected without reconsideration.

Anchor allocation and repair: Weekly anchor meals for fish or seafood, legumes, white meat, and red meat were assigned before the remaining slots were filled. Completed days underwent energy and fat checks, with up to two replacements per check. Energy-tolerance bands were 0.87–1.07 times the target for weight-loss plans, 0.88–1.12 for maintenance plans, and 0.93–1.17 for weight-gain plans.

Implementation and sensitivity analysis: The framework was implemented in Python. Pseudocode and full constraint specifications are provided in Supplementary File S1 Tables S3 and S4. Seed sensitivity was assessed by regenerating each of the six profiles using five seed sequences to yield 30 plans and comparing the resulting energy and nutrient outcomes.

### 2.7. Recipe Nutrient Calculations and Quality Control

Recipe-based nutrient values were calculated using a separate rebuild script. For each recipe, the script reviewed all ingredients and calculated the total nutrient content from the amount of each ingredient used and its nutrient values per 100 g in the standardized food-composition library. For nutrients that may be lost during cooking, such as water-soluble vitamins and ethanol, cooking-method-specific retention factors were applied (Supplementary File S1 Table S5). These retention factors were taken from the USDA Table of Nutrient Retention Factors, Release 6, which provides reference values for nutrient losses during food preparation [42]. Each recipe was assigned a confidence tier—high, medium, or low—based on two gram-weighted scores computed by the rebuild script. The first, mass coverage (mc), is the percentage of the recipe’s non-seasoning ingredient weight that is accounted for by a successfully matched canonical food entry. The second, provenance quality (pq), is a gram-weighted average of each matched ingredient’s source-quality score multiplied by a mapping-confidence modifier (1.0 for high-confidence mappings, 0.85 for medium, and 0.5 for low). A recipe was assigned high if mc ≥ 95% and pq ≥ 80, medium if mc ≥ 80% and pq ≥ 55, and low in all other cases, including any recipe in which a non-seasoning ingredient had no valid canonical food match. Of the 252 recipe and side-dish entries in the final production library, 79.4% was classified as high-confidence. Recipes classified as low-confidence were excluded from the meal-selection process.

### 2.8. Output Reports

For each generated meal plan, the system produced two output reports. The first output was a summary report intended for the user. It presented each day’s meals and recipes in meal-by-meal tables, together with the daily amounts of energy and nutrients.

The second output was a detailed analysis report intended for healthcare professionals involved in the user’s dietary care. It included user characteristics, namely sex, age, BMI, weight goal, physical activity status, documented allergies, and dietary-plan length. It also included detailed nutritional analyses of the generated dietary plans, including daily energy content, daily macronutrient distribution, fat composition [43], n-3 fatty acid content [37], weekly energy-intake trend, daily micronutrient content compared with Dietary Reference Values [38,39] and the American Heart Association [40], daily essential amino acid content [44], cooking-related nutrient-retention analysis using USDA-derived retention values [42], and the omega-6-to-omega-3 fatty acid ratio [45,46]. The report also included GR-UPFAST scoring, developed by our team, to assess the ultra-processed food contents of recipes [47].

Both reports read the same recipe nutrient file; the day-by-day values in the user summary and the daily trajectories charted in the deep analysis are therefore identical by design.

### 2.9. Production of Recipe-Based Dietary Plans and Evaluations

Six 28-day recipe-based dietary plans were produced for six hypothetical individuals. The plans covered daily energy goals from 1600 to 2600 kcal/day and included four female and two male profiles (Table 3). Their ages ranged from 28 to 35 years and BMI from 20.8 to 26.6 kg/m^2^. Energy goals represented a mild energy deficit for two plans and weight maintenance for the remaining four plans. All plans were alcohol-free and included three meals and two snacks per day. No real-patient data were used in any evaluated plan.

#### 2.9.1. Quality Assessment of the Produced Dietary Plans with Validated Scores

The quality of the six 28-day recipe-based dietary plans was assessed using three validated dietary indices. Mediterranean diet adherence was evaluated using the MedDietScore, which ranges from 0 to 55, with higher scores indicating greater adherence to the Mediterranean dietary pattern [24]. The phytochemical content of the plans was assessed using the dietary phytochemical index, which ranges from 0 to 100 and reflects the percentage of daily energy derived from phytochemical-rich foods; higher values indicate greater contribution from plant-derived bioactive compounds [25]. The ultra-processed food content was assessed using GR-UPFAST, which ranges from 0 to 70, with higher scores indicating a greater ultra-processed food intake [47].

#### 2.9.2. Comparison with Direct LLM Generation

For comparison, Claude Sonnet 5 (Anthropic, San Francisco, CA, USA) directly generated one 28-day dietary plan for each of the same six profiles without a deterministic computational layer. A single generation was used per profile in both study arms, without regeneration or selection. The prompt specified the participant profile, plan duration, five daily eating occasions, Mediterranean-style and alcohol-free requirements, ingredient quantities, and reporting format, but provided no nutrient targets or compliance criteria. The same criteria (i.e., energy, carbohydrate, protein, fat, monounsaturated fatty acids, marine n-3, vitamin C, vitamin B12, folate, iron, calcium, fiber, and sodium) were subsequently applied to both sets of plans. The complete prompt and comparison are provided in Supplementary File S2.

#### 2.9.3. Early-Stage Acceptability of the Produced Dietary Plans by Healthcare Professionals

Study design and methods: Healthcare professionals, including registered dietitians, physicians, pharmacists, and nurses, were invited via electronic communications distributed to healthcare facilities across Greece, including hospitals, clinics, and dietetic practices. Each participant was asked to evaluate one of the six 28-day dietary plans. Random allocation of the six dietary plans was performed by an external researcher independent of the evaluation process, using a 1:1:1:1:1:1 allocation ratio among the invited healthcare professionals. Participants were asked to review the assigned plan and complete an anonymous acceptability questionnaire administered through Google Forms. The questionnaire was purpose-built for this evaluation, which was conducted from April to June 2026. The questionnaire is provided in the Supplementary Materials (Supplementary File S1 Table S6). The questionnaire comprised two parts. Part A included informed consent and five items on the assigned dietary plan, respondent sex, and previous use of AI tools. Part B comprised items assessed on a 5-point Likert scale, i.e., not at all = 1, slightly = 2, moderately = 3, very = 4; very much = 5, and included four domains that reflected: nutritional adequacy and structure of the dietary plan (Domain 1; six items); practical and professional applicability of the dietary plan (Domain 2; five items); recipe quality and Mediterranean compatibility (Domain 3; 8 items); and documentation, nutritional analysis, and audit-file quality (Domain 4; five items).

Ethics: For ethical purposes, Part A included an information and consent page describing the study aim, voluntary participation, confidentiality, estimated completion time, and use of data for research. Participants provided electronic informed consent before proceeding to Part B. Only consenting participants were included in the analysis.

Primary outcome and sample size: The primary acceptability outcome was the proportion of healthcare professionals who rated each item as satisfactory, defined as a score of 4 or 5 on the 5-point scale. A threshold of at least 70% satisfactory ratings was set to indicate acceptable performance, representing agreement by a clear majority of respondents and following approaches used in early-stage acceptability evaluations of AI-based clinical decision-support tools [48]. Based on an expected acceptability rate of 70%, 81 completed questionnaires were required to estimate this proportion with a 95% confidence interval of approximately ±10 percentage points. To allow for about 15% incomplete or unusable questionnaires, we aimed to recruit at least 96 healthcare professionals.

Statistical analysis: All Likert-type variables were treated as ordinal variables and were presented as frequencies, percentages or medians and interquartile ranges. “Positive responses” were defined as ratings of 4 or 5 (i.e., “much” or “very much”). Domain scores were calculated as mean scores on a 1–5 scale. An overall professional acceptability score was calculated from the evaluation items, excluding the item assessing applicability outside Mediterranean populations, which was analyzed separately. Internal consistency was evaluated using Cronbach’s alpha. Exploratory comparisons between the six meal plans and score domains were performed using Kruskal–Wallis tests (for variables with three or more groups). Statistical significance was set at *p*-value < 0.05. All statistical analyses were performed using the SPSS software, version 31.0 (IBM Corp., Armonk, NY, USA).

## 3. Results

### 3.1. Compliance of the Produced Dietary Plans with Design Specifications

**Figure 3:**
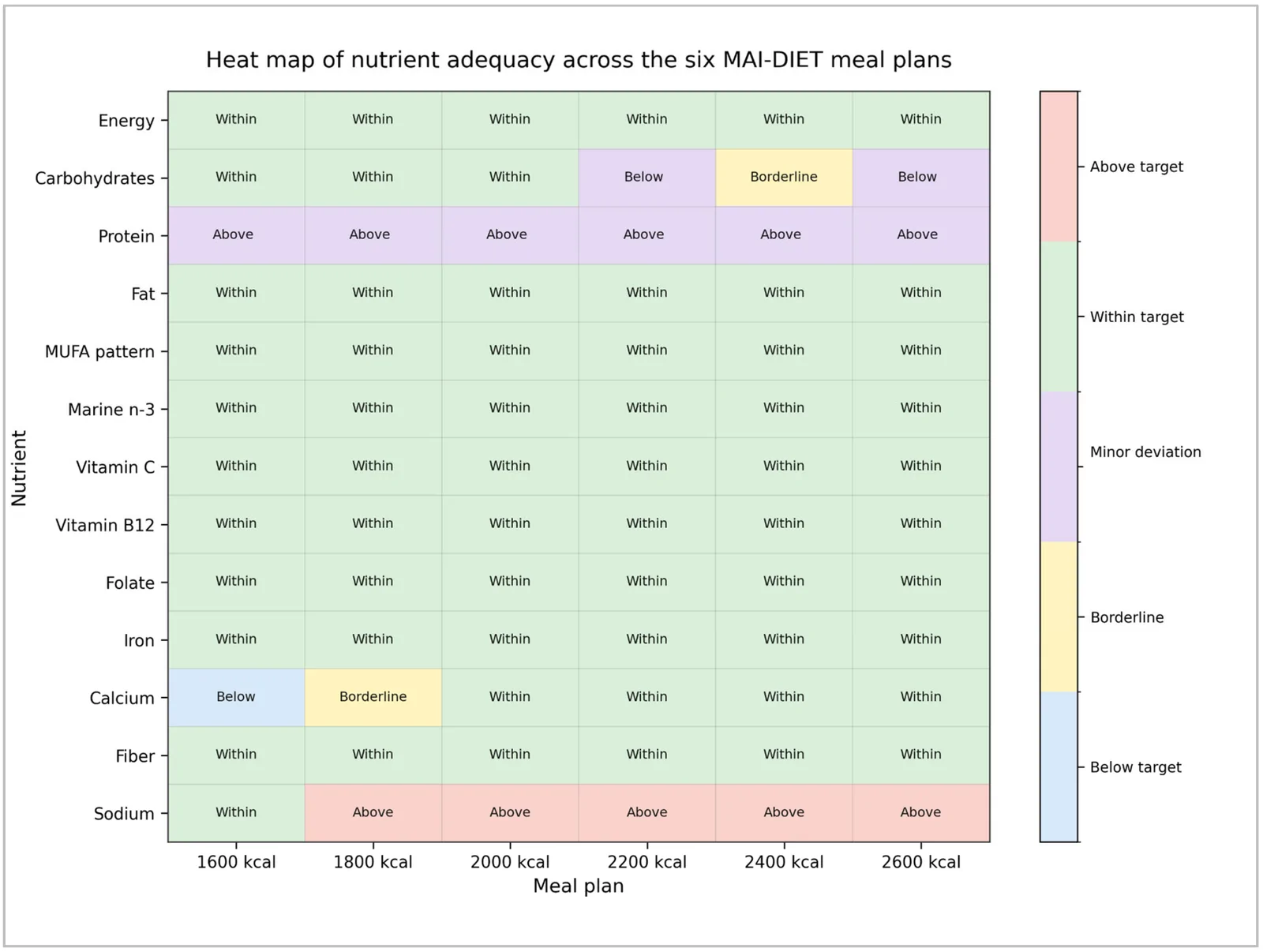
Colors indicate nutrient status relative to the predefined targets based on mean daily intake. “Borderline” denotes a deviation of <1% from the predefined target, whereas “minor” denotes a deviation of ≥1% but <10%. “Above or below” denotes a deviation of ≥10%. MUFA, monounsaturated fatty acid. Full mean ± SD values for the meal plans are provided in Supplementary File S1 Table S7.

Compliance of the generated dietary plans with predefined nutrient goals [49,50,51,52] is shown in the following heat map (Figure 3) and in Supplementary File S1 Table S7. All plans met the predefined energy criteria. Carbohydrate goals were met in the 1600 kcal and 2000 kcal (with marginal deviation) plans. At higher energy levels, carbohydrate was 1.9% and 2.4% below the predefined range in the 2200 and 2600 kcal plans, respectively, while the 2400 kcal plan was borderline (0.9% below). Protein was modestly above the upper target in all six plans, with deviations ranging from 5.5% to 7.5%. Fat targets and the MUFA-dominant pattern were achieved across all plans. The goals for marine n-3 fatty acids, vitamin C, vitamin B12, folate, and iron were also met. Sodium deviations were most pronounced in the 2200–2600 kcal plans (+24.9% to +36.3%). Calcium was 17.5% below the EFSA population reference intake in the 1600 kcal plan and borderline in the 1800 kcal plan.

Numerical consistency in macronutrient content between the user-facing and healthcare-professional-facing outputs was also confirmed. Supplementary File S1 Table S8 provides a representative day-by-day example for the 2000 kcal meal plan, demonstrating that macronutrient values were closely matched across the two output reports.

The technical performance of the framework was assessed independently of the professional evaluation. Recipe nutrient values were verified by recomputing a random sample of 25 recipes from ingredient masses, per-100 g values in the standardized food-composition library, and the applicable cooking-retention factors, using a script written independently of the production pipeline; all 100 field comparisons matched within rounding, with a mean absolute difference of 0.0012% and no recipe differing by more than 1%. All 28 days finished within the predefined energy tolerance band in each of the six plans. Generation of one complete 28-day plan required 1.5–1.8 s of wall-clock time on a consumer desktop computer, excluding document export. Across the 252 recipe and side-dish entries, 200 (79.4%) were classified as high confidence, 34 (13.5%) medium, and 18 (7.1%) low, with low-confidence entries excluded from recipe selection. These metrics are consolidated in Supplementary File S1 Table S9.

The seed-sensitivity analysis showed that energy and macronutrient outcomes were effectively insensitive to the random seed. Across all six profiles, the relative ranges did not exceed 3.2% for mean daily energy or 4.4% for the percentage of energy derived from any macronutrient; protein remained close to 20% of energy, carbohydrate between 44% and 47%, and fat between 35% and 38% in every run. Several micronutrients showed greater variability: marine n-3 fatty acids and vitamin C varied by up to 19.6% and 19.4%, respectively, while vitamin B12 and folate varied by up to 12.9% and 12.3%, respectively. In the 1800–2600 kcal plans, sodium remained above the WHO and AHA targets across all tested seed sequences. Calcium in the 1600 kcal plan remained below the EFSA population reference intake in every run. The full results are provided in Supplementary File S1 Table S10.

### 3.2. Quality Assessment of the Produced Dietary Plans with Validated Scores

Validated tools to assess the quality of the generated plans were applied, namely MedDietScore, DPI, and GR-UPFAST (Table 4). All six plans had a MedDietScore of 35 or greater, corresponding to good Mediterranean diet adherence [48]. Energy derived from foods rich in phytochemicals accounted for approximately 50% of the total energy content, while the content in ultra-processed foods was minimal, namely less than five out of 70.

### 3.3. Comparison with Direct LLM Generation

Compared with the six direct LLM plans, the MAI-DIET plans showed less repetition, fewer placeholder meal names, and more consistent daily energy content (Table 5). Exact repetition of an earlier day occurred on 21 of 168 comparator-plan days but on none of the MAI-DIET days. Maximum dish use was 27 versus seven times per plan and seven versus two times per week. Generic placeholders occurred in 88 of 840 comparator-meal slots versus 23 of 840 MAI-DIET slots. Daily energy variability was also lower overall with MAI-DIET, with a maximum within-plan range of 233 kcal compared with 721 kcal for direct LLM generation.

### 3.4. Acceptability of the Produced Dietary Plans by Healthcare Professionals

As presented in Supplementary File S1 Table S11, the study sample included 103 healthcare professionals (71.8% females). The majority of healthcare professionals had previously used AI tools (78.6%) and 74.7% reported using digital applications several times per week or every day. The most evaluated meal plan was that of 1800 kcal/day (24.3%).

Domain scores and internal consistency are presented in Table 6. The overall professional acceptability score suggested a generally favorable evaluation of the MAI-DIET-framework outputs (mean score 3.84 ± 0.59). The highest rated domain was Domain 4 (i.e., the documentation) (mean score 4.01 ± 0.63), while the lowest rated domain was Domain 2 (i.e., practical and professional applicability). Internal consistency was very good to excellent across the domains, with Cronbach’s alpha values ranging from 0.838 to 0.952.

Item-level evaluation from the *N* = 103 healthcare professionals is presented in Supplementary File S1 Table S12. Overall, participants favorably rated most aspects of the MAI-DIET-generated dietary plan, with median scores of 4.0 for almost all items. Regarding the “nutritional adequacy and structure of the diet plan” domain (Domain 1), positive responses ranged from 69.9% for overall meal-plan balance to 81.6% for energy target adequacy. The “practical and professional applicability of the diet plan” domain (Domain 2) showed more moderate ratings, particularly for real-life applicability (54.4%) and use in professional practice (57.3%), whereas clarity of food and meal options was rated positively by 73.8% of participants. In “recipe quality and Mediterranean compatibility” domain (Domain 3), all items were well evaluated, especially questions about the alignment with the meal-plan rationale (84.5%) and the Mediterranean diet compatibility (86.4%). However, lower positive responses were observed for the professional usability of outputs (57.3%). The “documentation, nutritional analysis, and audit-file quality” domain (Domain 4) was also favorably evaluated, with positive responses ranging from 73.8% for user-specific file distinction to 85.4% for micronutrient analysis adequacy. Finally, perceived adaptability to non-Mediterranean populations received the lowest scores, with only 38.8% positive responses.

Health professional evaluation scores according to the energy level of the evaluated meal plan are presented in Supplementary Table S13. Overall acceptability did not differ significantly across the six energy levels (*p* = 0.190). Similarly, no statistically significant differences in the four domain scores were observed across the assigned meal-plan energy levels (all *p*’s > 0.05).

## 4. Discussion

This study developed MAI-DIET by combining human-curated Mediterranean recipe and food-composition libraries with a deterministic meal-planning engine. Six 28-day plans for hypothetical users were evaluated for nutrient adequacy and diet quality, compared with direct LLM-generated plans, and assessed by healthcare professionals. Energy and fat targets were met across all plans, while carbohydrates and proteins showed only minor deviations. Most micronutrient targets were achieved, with exceptions for sodium and calcium at specific energy levels. The plans showed high Mediterranean diet adherence, substantial phytochemical-rich food content, and minimal ultra-processed food content. Professional acceptability was generally favorable.

To contextualize the findings, the six MAI-DIET plans were compared with plans generated directly by an LLM for the same profiles. Both approaches produced mean daily energy values close to the requested targets—within 1.0% for direct LLM generation and 1.8% for MAI-DIET. However, MAI-DIET showed substantially less whole-day repetition, dish reuse, and day-to-day energy variation, reflecting the planner’s explicit constraints and repair checks. Because the nutrient values in the comparator plans were reported by the LLM and were not independently recalculated using a food-composition database, this comparison concerns structural consistency rather than nutrient accuracy.

The use of AI in dietary planning is growing rapidly, and several approaches have been reported in the recent literature. Studies have shown that LLM-generated meal plans can partially align with dietary guidelines, but that energy and nutrient calculations are a recurring source of inaccuracy and inconsistency [13,19,20]. The MAI-DIET framework addresses this challenge by separating what the AI does from what a deterministic calculation module does. This architecture is closer to rule-based clinical decision support than to LLM-generated dietary planning. A comparable approach combining AI-based recommendation logic with Mediterranean diet promotion has been reported [21]. Within this broader field of AI-assisted nutrition recommendation, MAI-DIET is distinguished by ingredient-level nutritional traceability, reviewed food-composition mappings, cooking-retention adjustments, and deterministic nutrient computation, thereby supporting auditable outputs and reproducible runtime generation under identical inputs. The evidence on LLM numerical inaccuracy cited above motivated the design of the present framework and was not intended as a direct performance comparison. The LLM was used for structured text transformation, data-preparation tasks, and user-interface access with all energy computations, recipe selections, and nutrient calculations being performed by a fixed rule-based engine operating on reviewed, version-controlled food-composition data. Human oversight was embedded throughout: dietitian reviews covered the recipe database, the food-composition library, and the generated plans before any outputs were evaluated.

The MAI-DIET framework was not designed to replace clinical judgment, it was designed to give a clinician a structured, auditable, and reproducible starting point. The nutritional reliability of MAI-DIET outputs rests on ingredient-level verification. Every ingredient in the recipe library was individually matched to a public reference database (FoodData Central and/or OpenNutrition), the quality of each match was recorded and propagated through gram-weighted calculations to assign each recipe a confidence tier—high, medium, or low. Recipes classified as low-confidence were excluded from plan generation. This means every nutrient value produced by the engine is traceable to a verified ingredient record rather than to an approximation or an estimated aggregate. Beyond ingredient-level accuracy, the engine encodes nutritional safeguards against monotony and macronutrient drift. A variety penalty discourages repeated selection of the same recipe, food-group frequency limits enforce alignment with the Greek National Dietary Guide, a fat-content penalty biases selection away from recipes that would push the daily fat distribution above an acceptable ceiling, and daily energy and fat-percentage are checked after plan assembly, with slot replacement applied if either fall outside the acceptable range. These mechanisms promote dietary variety and help limit energy and nutrient deviations. The framework also provides a dual-format output that serves both the patient and the supervising clinician from the same underlying calculation. The reproducibility of outputs is a direct property of the engine design rather than a goal pursued post hoc.

Dietary quality assessment using validated indices showed a systematic favorable pattern across all six evaluated plans. Mediterranean diet adherence, assessed using the MedDietScore [24], fell within the good adherence range. This finding is consistent with the intended purpose of the recipe library, which was designed to support and promote eating patterns aligned with the Mediterranean diet. Additionally, ultra-processed food content, assessed with the GR-UPFAST tool [47], was minimal in all plans, while the dietary phytochemical index indicated a substantial contribution from phytochemical-rich plant foods [25]. Together, these findings indicate that the MAI-DIET plans were consistent with favorable diet-quality characteristics.

All plans met the predefined energy and fat criteria and maintained a MUFA-dominant profile [49,50,52,53]. Protein was modestly above the predefined target across all plans, while carbohydrate was slightly below the target in the higher energy plans (2200–2600 kcal/day). These findings should be interpreted as small deviations from study-specific targets rather than as evidence of overall dietary inadequacy. Mediterranean dietary patterns can accommodate variation in macronutrient distribution across populations, and clinical studies have reported metabolic benefits from Mediterranean diets with higher protein, lower carbohydrate, or modified fat contents [49,50,52]. Moreover, evaluation of the Healthy Diet Basket showed that healthy dietary patterns can achieve high overall nutrient adequacy while allowing variation in individual nutrient intakes [51]. The criteria for vitamin C, vitamin B12, folate, iron, fiber, and marine n-3 fatty acids were met across all plans. This finding is consistent with evidence associating Mediterranean diet adherence with greater micronutrient adequacy and higher n-3 fatty-acid intake, reflecting the contribution of vegetables, fruits, legumes, nuts, fish, and seafood [54,55,56]. Calcium content in the 1600 kcal plan was below the EFSA population reference intake, which may be clinically relevant for individuals with similarly low energy requirements [57].

The sodium content exceeded WHO and AHA recommendations, particularly for meal plans ≥ 2200 kcal, which could be attributed to the inherent sodium content of Greek–Mediterranean recipes and the nature of the Mediterranean dietary pattern at higher energy levels [58], rather than an engine error. The current planner does not apply a sodium ceiling or calcium floor during recipe selection; both are planned for future development. These deviations persisted across all tested seed sequences at higher energy levels, suggesting that they reflected the plan structure rather than the specific seed sequence used.

The acceptability ratings by the healthcare professionals were generally favorable across the four domains, with most items achieving a median score of 4.0, indicating overall agreement regarding the nutritional adequacy, Mediterranean compatibility, and documentation quality of the dietary plans. The highest positive responses were observed for Mediterranean diet compatibility, alignment with the meal-plan rationale, and micronutrient analysis adequacy. These observations may suggest that Greek healthcare professionals perceived the plan as nutritionally sound and culturally appropriate for Mediterranean settings. However, the lower rating for adaptability to non-Mediterranean populations should be interpreted cautiously, as all evaluators were Greek healthcare professionals and may not reflect the perspectives of culturally diverse populations. Adaptation may require locally available and culturally familiar foods while preserving the Mediterranean diet’s core nutritional characteristics; therefore, its transferability should be assessed with healthcare professionals and intended users from the relevant populations [59]. Nevertheless, the findings of the present study support the technical feasibility of the framework and its preliminary acceptability among healthcare professionals.

Several limitations should be acknowledged. Recruitment through electronic invitations may have favored digitally engaged professionals. Moreover, all participants practiced in Greece; therefore, the findings may not be generalizable to healthcare professionals in other settings. The seed-sensitivity analysis included only five seed sequences across six profiles, and the resulting ranges should be considered indicative rather than interpreted as confidence intervals. Variation in several micronutrient outcomes across seeds also indicated that values from a single generated plan should not be regarded as fixed properties of the framework. The “Persephone” planner selected recipes sequentially and permitted only limited subsequent revision; therefore, it cannot guarantee the optimal combination of meals across an entire day or week. Future development could incorporate constraint or integer programming to support joint optimization and sensitivity analyses using more seeds. Finally, evaluation in real-world users will be important to establish the framework’s usability, acceptability, adherence, and practical effectiveness.

## 5. Conclusions

These early-stage findings support the feasibility of combining LLM-assisted data preparation with deterministic computation. This approach generated recipe-based Mediterranean dietary plans that broadly met energy and nutrient targets and aligned with established diet-quality indices. Across six 28-day meal plans ranging from 1600 to 2600 kcal/day, the framework achieved good adherence to the Mediterranean diet, a substantial contribution from phytochemical-rich foods, and minimal ultra-processed food content. Feedback by practicing healthcare professionals was generally favorable, with documentation quality receiving the highest ratings. Clinical evaluation, including comparisons with dietitian-generated plans or guideline-based systems, is needed to establish the framework’s usability, acceptability, effects on dietary adherence, and practical effectiveness.

## Figures and Tables

**Table 1:** LLM tasks in the MAI-DIET framework.

LLM Task	Explanation	Example
Hypothetical profile creation	Assisted in the creating the hypothetical profiles (age, sex, body mass, height, and physical activity level) used to generate the six evaluated 28-day plans.	Specified the profile for the 2000 kcal male scenario. The senior dietitian reviewed and approved the values before plan generation.
Resolution of inconsistent profile inputs	Proposed how to resolve contradictory or incomplete profile values before plan generation.	Resolved a conflict between a stated activity description and the assigned PAL category. The approved resolution was recorded before the plan was generated.
Standardized food-library review	Reviewed food-composition entries and ingredient mappings against FoodData Central descriptions and flagged entries for correction.	Identified that a canned-legume entry should reference the drained rather than undrained nutritional record.
Diet-quality scoring-file preparation	Mapped each food item in the recipe library to its category in the MedDietScore, DPI, and GR-UPFAST scoring rubrics.	Assigned chickpeas to the legume component of the MedDietScore rubric [24] and to the plant-protein category of the DPI rubric [25].
Per-day energy override suggestion	Suggested the scale factor to apply when a daily plan fell outside the energy-tolerance criteria applied by the module (0.87–1.07 × target for weight-loss plans, 0.88–1.12 × target for maintenance plans, 0.93–1.17 × target for weight-gain plans). The “Persephone” module executed all recalculations.	Proposed scaling portion sizes upward for a day that fell below the lower tolerance boundary. The human operator approved the adjustment before it was logged.
ETL support for recipe-database development	Converted raw recipe text into structured database entries following predefined rules for gram conversion, ingredient naming, and cooking-method classification.	Converted the ingredient entry “400 g, chopped tomatoes” into a canonical record linked to the food library.
Interface for deterministic meal-plan generation	Served as the interface through which the human operator executed the deterministic “Persephone” module. The operator supplied the profile parameters; the language model launched the module by issuing a programmatic call to its plan-generation function and returned the resulting files.	Executed the module for the 2000 kcal male profile by issuing a programmatic call with the operator-supplied parameters and the module’s default seed sequence [42, 123, 456, 789], returning the generated plan and reports unchanged.

The LLM supported predefined data-transformation tasks during system development and served as the operator-facing interface for initiating the planning process while under explicit operator supervision. All LLM-supported outputs were reviewed before use. The LLM was not used during runtime meal-plan generation or nutrient calculation. LLM: large language model; BMI: body mass index; PAL: physical activity level [26].

**Table 2:** Single-ingredient pipeline trace: tomato in the recipe “Green Bean and Potato Olive Oil Stew.”

Step	Pipeline Stage	Action Performed	Action Purpose
1	Original recipe source	The original recipe listed tomato as: “400 g, chopped tomatoes.”	This entry provided the original ingredient quantity before database structuring.
2	Structured recipe entry	Tomato was entered into the recipe database with a quantity of 400 g. It was classified as a core ingredient and marked as consumed in the final dish.	This step allowed the system to calculate the ingredient’s contribution to the recipe’s total energy and nutrient content.
3	Food-name standardization	The ingredient name “tomatoes” was standardized to match the food-composition library.	This step created the standardized ingredient name used for food-composition matching.
4	Ingredient mapping	“Tomatoes” were linked to the standardized food entry. The match was classified as high confidence because it was an exact ingredient match.	This step linked the recipe ingredient to the correct standardized food entry and recorded the confidence level of the match.
5	Food-composition entry	The standardized food entry was tomato, linked to FoodData Central ID and the corresponding OpenNutrition entry.	This was the reference source for tomato nutrient values used in the calculation.
6	Nutrient values per 100 g	The selected food-composition entry provided nutrient values per 100 g: 18.8 kcal, 14.8 mg vitamin C, and 21 µg folate.	These per-100 g values were used as the basis for calculating the energy and nutrient contribution of the ingredient.
7	Cooking-retention adjustment	Because the recipe involved long simmering, cooking-retention factors were applied to heat-sensitive nutrients. Vitamin C and folate were both multiplied by 0.50, based on USDA retention factors.	This step adjusted selected nutrient values to account for expected nutrient losses during cooking.
8	Final recipe-level contribution	For 400 g of tomato, the final contribution to the recipe was 75.2 kcal, 29.6 mg of vitamin C, and 42 µg of folate. Vitamin C and folate were lower than pre-cooking values because retention factors were applied.	This final value was used in the total nutrient calculation for the complete recipe and in dietary-plan outputs.

This example shows how the ingredient in a recipe was linked to nutrient data from a public food-composition database. The cooking method was used to adjust nutrient values where needed, and the quality of the ingredient match was used to assign a confidence level to the recipe.

**Table 3:** Summary for the six 28-day plans produced for hypothetical individuals.

Plan	Target (kcal/d)	Sex	Age (y)	Height (cm)	Body Mass (kg)	BMI	PAL	Goal
1	1600	F	35	155	50	20.8	1.375 (light)	Maintenance
2	1800	F	35	163	65	24.5	1.55 (mod)	Mild loss
3	2000	M	35	170	72	24.9	1.55 (mod)	Mild loss
4	2200	F	35	168	75	26.6	1.55 (mod)	Maintenance
5	2400	F	28	165	64	23.5	1.725 (very)	Maintenance
6	2600	M	30	178	67	21.1	1.725 (very)	Maintenance

BMI: body mass index, PAL: physical activity level [26]; F: female; M: male; y: years.

**Table 4:** Dietary quality scores of MAI-DIET-generated dietary plans.

Dietary Plan	MedDietScore (0–55; Higher = Better Adherence)	DPI % (Higher = More Plant-Derived Bioactive Compounds)	GR-UPFAST (0–70; Lower = Less UPF)
1600 kcal/day	36	50.9	2.0
1800 kcal/day	36	49.5	3.0
2000 kcal/day	35	49.6	3.5
2200 kcal/day	36	47.0	4.5
2400 kcal/day	36	48.0	4.0
2600 kcal/day	36	48.9	4.5

DPI: dietary phytochemical index, UPF: ultra-processed foods.

**Table 5:** Error classes observed in six 28-day meal plans generated by direct large-language-model generation and in the six MAI-DIET plans produced for the same profiles.

Error Class	Direct LLM Generation (Six Plans)	MAI-DIET (Six Plans)	MAI-DIET Safeguard
Days repeating an earlier day in all five eating occasions	21 of 168 days; in one plan days 15–28 repeat days 1–14 ingredient for ingredient	0 of 168 days	C1, C2, and C9: A recipe cannot recur within a day, cannot exceed two uses per calendar week, and cannot occupy the same slot on consecutive days
Maximum uses of one dish per plan	3 to 27 over 28 days; 1 to 7 within one calendar week	6 to 7 over 28 days; 2 within one calendar week in all six plans	C2: Hard cap of two uses per recipe per calendar week, applied before scoring
Meal slots named by a generic placeholder	88 of 840 slots; 21 distinct placeholder names	23 of 840 slots; 1 distinct placeholder name	Dish names are fields of the curated recipe library; the engine does not generate names
Day-to-day variation in energy	SD 23.0 to 183.9 kcal; widest plan spanned 721 kcal between its lightest and heaviest day	SD 33.9 to 62.3 kcal; widest plan spanned 233 kcal	Each completed day is re-checked against a fixed tolerance band around the daily target, and a non-anchor meal is exchanged when the day falls outside it

SD: standard deviation. C1, C2, and C9 are the constraint identifiers used in the MAI-DIET planning module and are specified in Supplementary File S1 Table S4. Each system produced 6 × 28 × 5 = 840 core meal slots.

**Table 6:** Domain scores and internal consistency.

Domains	N of Items	Mean ± SD	Median (IQR)	Range	Cronbach’s Alpha
Domain 1	6	3.89 ± 0.68	4.0 (3.67–4.33)	1.5–5.0	0.892
Domain 2	5	3.58 ± 0.83	3.8 (3.0–4.0)	1.4–5.0	0.894
Domain 3	8	3.85 ± 0.57	3.9 (3.59–4.25)	2.0–5.0	0.871
Domain 4	5	4.01 ± 0.63	4.0 (3.75–4.45)	2.0–5.0	0.838
Overall professional acceptability	24	3.84 ± 0.59	3.9 (3.58–4.21)	1.9–5.0	0.952

Domain scores were calculated as mean composite scores on a 1–5 scale. Domain 1: Nutritional adequacy and structure of the diet plan; Domain 2: Practical and professional applicability of the diet plan; Domain 3: Recipe quality and Mediterranean compatibility; Domain 4: Documentation, nutritional analysis, and audit-file quality. The perceived generalizability item was not included in the overall score.

## Data Availability

The source code of the MAI-DIET framework, the standardized food-composition and recipe libraries, and the six generated 28-day meal plans are available from the corresponding author on reasonable request. No real-patient data were used at any stage of this study.

## Supplementary Materials

The following supporting information can be downloaded at: https://www.mdpi.com/article/10.3390/nu18162661/s1. Supplementary File S1 [Table S1. Equation-selection rule applied for the estimation of basal metabolic rate; Table S2. Recipe selection scoring rule; Table S3. Pseudocode for 28-day plan generation and recipe selection; Table S4. Specification of the constraints applied during recipe selection; Table S5. Applied cooking-method-specific retention factors for nutrient losses during cooking; Table S6. Questionnaire to evaluate the acceptability of the MAI-DIET framework from healthcare professionals; Table S7. Comparison of nutrient goals with MAI-DIET-generated daily nutrient outputs across six energy-level meal plans; Table S8. Day-by-day energy and macronutrient output for the 2000 kcal plan (Case 3, male, 28 days), with comparison between the user-facing summary report and the healthcare-professional-facing deep analysis report; Table S9. Consolidated technical performance metrics of the MAI-DIET framework; Table S10. Seed-sensitivity analysis of generated plan outcomes across five seed sequences; Table S11. Participant characteristics, AI experience, digital application use, and assigned meal-plan energy level among health professionals (*N* = 103); Table S12. Item-level professional evaluation of the MAI-DIET-generated monthly meal plan and recipes; Table S13. Health professional evaluation scores according to the energy level of the evaluated meal plan]; Supplementary File S2 [Table S1. Energy outcome and criteria compliance for both systems across the six matched profiles; Figure S1. Side-by-side compliance heat maps for the six matched plans; Figure S2. Compliance of the 1600 kcal/day language-model plan against the thirteen reference criteria applied in Table S7; Figure S3. Compliance of the 1800 kcal/day language-model plan against the thirteen reference criteria applied in Table S7; Figure S4. Compliance of the 2000 kcal/day language-model plan against the thirteen reference criteria applied in Table S7; Figure S5. Compliance of the 2200 kcal/day language-model plan against the thirteen reference criteria applied in Table S7; Figure S6. Compliance of the 2400 kcal/day language-model plan against the thirteen reference criteria applied in Table S7; Figure S7. Compliance of the 2600 kcal/day language-model plan against the thirteen reference criteria applied in Table S7; Table S2. Eight error classes measured in the six directly generated plans and in the six MAI-DIET plans produced for the same profiles; Table S3. Conditions under which the comparator plans were generated; Supplementary Section S1. The comparator prompt as issued.].

## Abbreviations

The following abbreviations are used in this manuscript:

AI	Artificial Intelligence
BMI	Body Mass Index
BMR	Basal Metabolic Rate
DPI	Dietary Phytochemical Index
ETL	Extract–Transform–Load
LLM	Large Language Model
PAL	Physical Activity Level
TEE	Total Energy Expenditure
