# Supplementary material for: Clinician–Artificial Intelligence Collaboration for Mediterranean Meal-Plan Generation: Development, Technical Feasibility, and Professional Acceptability of the MAI-DIET Framework
Source: Nutrients. 2026 Aug 14;18(16):2661. doi: 10.3390/nu18162661 (PMC13516381; doi:10.3390/nu18162661)
Supplement: Supplementary file 1 [file nutrients-18-02661-s001.zip › nutrients-4447558_MAI-DIET_R1_Supplementary File S1.pdf]

Supplementary File S1

Supplementary Table S1. Equation-selection rule applied for the estimation of basal metabolic rate.

User characteristics	Equation	Reference
**BMI ≥ 30 kg/m², male**	BMR = 10W + 6.25H - 5A + 5	Mifflin-St Jeor et al. (1990) [29]
**BMI ≥ 30 kg/m², female**	BMR = 10W + 6.25H - 5A – 161	Mifflin-St Jeor et al. (1990) [29]
**BMI < 30 kg/m², age ≥ 65, male**	BMR = 13.5W + 5.003H − 4.5A + 542	Henry (2005) [30]
**BMI < 30 kg/m², age ≥ 65, female**	BMR = 10.1W + 3.776H − 1.5A + 506	Henry (2005) [30]
**BMI < 30 kg/m², age < 65, male**	BMR = 88.362 + 13.397W + 4.799H − 5.677A	Harris-Benedict Revised (Roza & Shizgal, 1984) [31]
**BMI < 30 kg/m², age < 65, female**	BMR = 447.593 + 9.247W + 3.098H − 4.330A	Harris-Benedict Revised (Roza & Shizgal, 1984) [31]

W: weight in kg; H: height in cm; A: age in years; BMI: body mass index.

Supplementary Table S2. Recipe selection scoring rule.

$S(r) = d\_kcal(r) + 150 \times c\_cum(r) - 20 \times I[fg(r) \in P] + j + p\_fat(r)$		
**Term**	**Meaning**	**Role in recipe selection**
S(r)	Total score for recipe r.	The recipe with the lowest score was selected.
d_kcal(r)	Absolute difference between the recipe energy content and the energy target for that meal or snack, capped at 150 kcal.	Smaller values indicate a closer energy match.
c_cum(r)	Cumulative count of how many times recipe r, or a similar recipe, had already been used.	The term is multiplied by 150 to penalize repetition and promote variety.
I[fg(r) ∈ P]	Indicator for whether the food group of recipe r is already represented in the set P of food groups used in the plan so far.	If true, 20 points are subtracted from the score, favouring cohesion with food groups already used in the plan.
j	A random value drawn uniformly from [−10, +10], added to every candidate score.	Prevents deterministic selection when scores are otherwise equal; applied to all candidates, not only to tied ones.
p_fat(r)	Penalty applied when adding recipe r to the day would cause the projected daily fat intake to exceed 35% of energy. Computed as (projected fat% − 35) × 8; zero otherwise.	Penalises selections that would push the day's projected fat content above 35% of energy.

When the day's cumulative energy at the time of recipe selection is below 70% of the daily target, the slot-level energy target is raised by 8% to bias selection toward more energy-dense options [Persephone_planner_V4.py, line 1000: target_kcal = target_kcal * 1.08].

Supplementary Table S3. Pseudocode for 28-day plan generation and recipe selection.

A. Full 28-day plan generation

```
FUNCTION generate_monthly_plan(user, db, nutrition, seeds = [42, 123, 456, 789]):  # line 1466
   weeks = ceil(plan_days / 7)  # 28 days -> 4 weeks
   CUMULATIVE_COUNTS = empty counter  # cross-week variety memory (line 1477)
   all_weeks = []
   FOR week_index IN 0 .. weeks-1:
      seed = seeds[week_index MOD length(seeds)]  # week 1->42, 2->123, 3->456, 4->789 (line 1480)
      week = generate_weekly_plan(user with plan_days=7, db, nutrition, seed)
      renumber week's days to the global day number (day + week_index*7)  # line 1486
      all_weeks.append(week)
      CUMULATIVE_COUNTS += counts of every recipe_id used this week  # line 1489
   RETURN concatenation of all_weeks
FUNCTION generate_weekly_plan(user, db, nutrition, seed = 42):  # line 1136
   SEED the single global random generator with `seed`  # line 1139  <-- only randomness source
 # --- 1. Energy target ---
   IF user.daily_kcal_override is provided:  # line 1142
      daily_target = round(override)  # target is FORCED
   ELSE:
      bmr  = compute_bmr(sex, weight, height, age)  # HB-Revised / Mifflin / Oxford by cohort (line 316)
      tdee = round(bmr * activity_factor)  # line 399
      daily_target = adjust_for_goal(tdee, bmi, goal)  # +/- fixed kcal by goal & BMI, clamped (line 448)
   k = daily_target / 2000  # portion scaling constant (line 522)
 # --- 2. Structure & pools ---
   template = build_meal_template(daily_target, num_snacks, ...)  # per-day kcal split per slot (line 532)
   pools    = build_recipe_pools(load_recipe_data(db, nutrition), ...)  # line 1176-1184
 # pools are lists built by iterating the recipe DataFrame IN ROW ORDER (line 771)
 # every recipe's nutrients are pre-scaled by k
   attach the 13 fixed sides to pools["side"]  # line 1187
 # --- 3. Weekly anchors (pre-assignment) ---
   anchors = allocate_anchors(pools, plan_days, goal, k)  # line 1226 / 1088
 # for each week, walk ANCHOR_TEMPLATE (fish x2, legume x2, white-meat x2, red-meat x1)
 # pick, per (day,slot), the top-3 recipes of the required category nearest the
 # slot's target kcal, then random.choice among those 3  # line 1120-1125
   week_used = counter; category_counts = counter; breakfast_type_counts = counter
   previous_day_slots = {}  # for the consecutive-day rule (C9)
 # --- 4. Day-by-day construction ---
   FOR day IN 1 .. plan_days:
      day_meals = []
      slot_order = [lunch_main, dinner_main, breakfast, snack_1, snack_2,  # line 1242-1252
                  (lunch_appetizer if enabled), (desserts if scheduled)]
      main_meal_deficit = 0
      FOR slot IN slot_order:
         slot_target = template target for (day, slot)  # line 1258
         IF slot is a snack AND main_meal_deficit > 0:  # line 1260-1263
            slot_target += main_meal_deficit / (remaining snack slots)  # redistribute shortfall
 # 4a. try the pre-assigned anchor for this slot, if still legal
         recipe = anchors[(day,slot)] IF it passes C1/C2/C9/C5 ELSE none  # line 1267-1278
 # 4b. otherwise run the greedy selector
         IF recipe is none:
            SET selection context (slot, day_meals, previous_day_slots, ...)  # line 1283-1293
```

```
            recipe = select_meal(pool for this slot, slot_target, tol=0.20, ...)  # line 1297
            IF recipe is none AND a category filter was applied:
                recipe = select_meal(... same, but with no category filter)  # line 1310-1323
        IF recipe is none: skip this slot  # line 1325-1326
        append recipe to day_meals; mark day_used / week_used / counters  # line 1339-1354
        IF slot is lunch_main or dinner_main:
            main_meal_deficit += (slot_target - recipe.kcal)  # line 1357-1359
            side = select_side(recipe, slot_target - recipe.kcal, ...)  # attach side if main is >=120 kcal short (line 1362)
            IF side: append side; reduce main_meal_deficit by side kcal  # line 1372-1410
  # 4c. post-assembly repair (bounded)
      day_meals = validate_day(day_meals, daily_target, goal, pools, week_used, k)  # line 1426
      sort day_meals into canonical slot order; compute day totals & in-tolerance flag  # line 1428-1442
      previous_day_slots = {slot -> recipe_id} from this day  # line 1445
   RETURN DataFrame(all days) , text_report
```

validate_day — bounded local repair (line 1495)

```
FUNCTION validate_day(day_meals, target, goal, pools, week_used, k):
   (floor, ceiling) = tolerance_band(target, goal)  # e.g. lose: 0.87x .. 1.07x (line 589)
   REPEAT up to 2 times:  # fat repair (line 1616)
      IF day fat% > 40: attempt_swap(reason = "fat") ELSE stop
   REPEAT up to 2 times:  # energy repair (line 1621)
      IF floor <= day kcal <= ceiling: stop
      ELSE attempt_swap(reason = "kcal")
   RETURN day_meals
FUNCTION attempt_swap(reason):  # line 1528
   order the NON-ANCHOR meals as the swap candidates:
      reason == "fat"  -> highest fat-density first
      reason == "kcal" & day over ceiling  -> highest-kcal first
      reason == "kcal" & day under floor   -> lowest-kcal first
   FOR each old_meal in that order:
      replacement = select_meal(same slot's pool, old_meal's slot target, ...,  # line 1567
                                excluding old_meal's id)
      IF no replacement or same id: continue
      build the candidate day with replacement swapped in
      ACCEPT only if it strictly improves the objective:  # line 1589-1596
         reason == "fat"  -> new day fat% < old day fat%
         reason == "kcal" -> |new day kcal - target| < |old day kcal - target|
      IF accepted: commit swap, update counters, RETURN true
   RETURN false  # no improving swap found
```

B. `select_meal` — the greedy per-slot selector (line 1016)

```
FUNCTION select_meal(pool, target_kcal, tolerance, day_used, week_used,
                day_groups, plan_food_groups, cumulative_counts,
                category_counts, category_caps, food_category_filter):
   DEFINE eligible(recipe, band):  # hard feasibility filter (line 1035)
      REJECT if recipe.id in excluded_recipe_ids  # line 1036
      REJECT if recipe == yesterday's recipe in this slot   (C9)  # line 1040
      REJECT if recipe.id in day_used                    (C1)  # line 1042
      REJECT if week_used[recipe.id] >= 2                 (C2)  # line 1044
      REJECT if food_category_filter set and category mismatch  # line 1046
      REJECT if recipe is a main and its category cap is reached (C5)  # line 1048
      REJECT if breakfast/snack and its similarity-group already used today (C3)  # line 1052
      REJECT if snack, nut-based, and a nut snack already used today (C4)  # line 1056
```

```
      REJECT if breakfast and this breakfast-type used >=2 this week (C6)  # line 1058
      REJECT if adding it makes a >=3-meal day exceed 50% fat (C8)  # line 1062
      IF band is "none": ACCEPT (no energy constraint)  # line 1065
      ELSE ACCEPT only if target*(1-band) <= recipe.kcal <= target*(1+band)  # line 1067-1069
 # relaxation ladder: widen the energy band step by step, finally drop it
   FOR band IN [tolerance, tolerance+0.10, tolerance+0.20, tolerance+0.30, none]:  # line 1071
      candidates = [r in pool where eligible(r, band)]
      IF candidates non-empty: BREAK  # accept the first band that yields any feasible recipe
   IF candidates empty: RETURN none  # line 1078
 # among the feasible candidates, pick the single best by the score (lower = better)
   RETURN argmin over candidates of score_candidate(recipe, target_kcal,  # line 1081-1085
                                     plan_food_groups, cumulative_counts)
FUNCTION score_candidate(recipe, target_kcal, plan_food_groups, cumulative_counts):  # line 988
   IF the day so far is below 70% of the daily target:
      target_kcal = target_kcal * 1.08  # energy-centering nudge (line 999-1000)
   kcal_distance  = min(|recipe.kcal - target_kcal|, 150)  # capped at 150        (line 1002)
   variety_penalty= 150 * cumulative_counts[recipe.id]  # repeat penalty        (line 1003)
   overlap_bonus  = -20 if recipe's food group already appears in the plan else 0   (line 1004)
   jitter         = uniform_random(-10, +10)  # tie-breaking noise     (line 1005)
   fat_penalty    = (projected day fat% - 35) * 8   if that would exceed 35% else 0 (line 1006-1012)
   RETURN kcal_distance + variety_penalty + overlap_bonus + jitter + fat_penalty  # line 1013
```

Language-neutral pseudocode corresponding to the deterministic planner module. Each meal slot is filled by a single greedy selection, after which a bounded repair pass is applied to the assembled day. No global objective is optimized and no backtracking search is performed. Reading the selector plainly: for each slot the engine (1) filters the pool down to recipes that satisfy every hard rule at the tightest energy band it can, widening the band only if nothing qualifies; then (2) scores the survivors with a fixed weighted sum and takes the best one. It never revisits an earlier slot except through the bounded validate_day repair. There is no search tree, no backtracking, no global objective being maximized.

Supplementary Table S4. Specification of the constraints applied during recipe selection.

Identifier	Constraint applied	Stage of the pipeline
C1	A recipe may not be used more than once within the same day.	Eligibility filter, recipe selection
C2	A recipe may not be used more than twice within the same week.	Eligibility filter, recipe selection
C3	At breakfast and snack slots, a recipe may not be used if another recipe of the same similarity group has already been used on that day.	Eligibility filter, recipe selection
C4	No more than one nut-based snack may be used per day.	Eligibility filter, recipe selection
C5	Main dishes are subject to a weekly cap per food category.	Eligibility filter, recipe selection
C6	No more than two instances of a given breakfast type may be used per week.	Eligibility filter, recipe selection
C8	For days containing three or more meals, a recipe is rejected if adding it would take projected daily fat above 50% of energy.	Eligibility filter, recipe selection
C9	A recipe may not be used in the same meal slot on two consecutive days.	Eligibility filter, recipe selection
Energy band	A recipe is accepted only if its energy content falls within the slot energy tolerance band. Where no candidate qualifies, the band is widened in three successive steps of 10 percentage points and, at the final step, removed for that slot.	Eligibility filter, applied after C1–C9

Constraint identifiers follow the labelling used in the source code, in which no constraint is labelled C7. Constraints C1–C9 are structural and remain enforced at every step of the relaxation sequence; the slot energy band is the only constraint that is relaxed. Pseudocode for the selection procedure is provided in **Supplementary File S1**.

Supplementary Table S5. Applied cooking-method-specific retention factors upon nutrient losses during cooking.

Cooking method	Vit. C	Folate	Thiamin	Riboflavin	Niacin	Vit. B6	Ethanol
None or stirred	1.00	1.00	1.00	1.00	1.00	1.00	1.00
Simmered, short	0.75	0.80	0.80	0.90	0.85	0.85	0.60
Simmered, long	0.50	0.50	0.60	0.80	0.70	0.65	0.35
Poached	0.60	0.60	0.70	0.85	0.75	0.70	0.40
Braised	0.25	0.30	0.40	0.70	0.55	0.50	0.15
Baked, long	0.40	0.50	0.60	0.80	0.75	0.70	0.25

Values are the fraction of each nutrient retained after preparation based on USDA Table of Nutrient Retention Factors.

Supplementary Table S6. Questionnaire to evaluate acceptability of the MAI-DIET framework from healthcare professionals.

**Ερωτηματολόγιο Αξιολόγησης Διαιτολογίων και Συνταγών MAI.DIET**	
**Έχω διαβάσει και κατανοήσει τις παραπάνω πληροφορίες σχετικά με τη συμμετοχή μου στην έρευνα. Κατανοώ ότι η συμμετοχή μου είναι εθελοντική και συναινώ στη χρήση των απαντήσεών μου για ερευνητικούς σκοπούς.**	
**1**	☐ Ναι, συναινώ να συμμετάσχω
	☐ Όχι, δεν συναινώ να συμμετάσχω
**Δήλωση Διαιτολογίου**	
**2**	**Ποιο είναι το ημερήσιο ενεργειακό περιεχόμενο του διαιτολογίου που λάβατε στο email;**
	☐ 1600 kcal ☐ 1800 kcal ☐ 2000 kcal ☐ 2200 kcal ☐ 2400 kcal ☐ 2600 kcal
**Πληροφορίες Χρήστη**	
**3**	**Φύλο**
	☐ Άνδρας ☐ Γυναίκα
**4**	**Έχετε χρησιμοποιήσει στο παρελθόν εργαλεία Τεχνητής Νοημοσύνης (AI);**
	☐ Ναι ☐ Όχι
**5**	**Πόσο συχνά χρησιμοποιείτε ψηφιακές εφαρμογές γενικά;**
	☐ Ποτέ ☐ Σπάνια ☐ Περιστασιακά ☐ Αρκετές φορές/εβδομάδα ☐ Καθημερινά
**Μηνιαίο Διαιτολόγιο**	
**6**	**Το μηνιαίο διαιτολόγιο ανταποκρίνεται ικανοποιητικά στον προκαθορισμένο θερμιδικό στόχο;**
	☐ Καθόλου ☐ Λίγο ☐ Μέτρια ☐ Πολύ ☐ Πάρα πολύ
**7**	**Η κατανομή των θερμίδων στα επιμέρους γεύματα είναι ισορροπημένη;**
	☐ Καθόλου ☐ Λίγο ☐ Μέτρια ☐ Πολύ ☐ Πάρα πολύ
**8**	**Το μηνιαίο διαιτολόγιο περιλαμβάνει όλες τις βασικές ομάδες τροφίμων;**
	☐ Καθόλου ☐ Λίγο ☐ Μέτρια ☐ Πολύ ☐ Πάρα πολύ
**9**	**Το μηνιαίο διαιτολόγιο παρουσιάζει επαρκή ποικιλία τροφίμων και γευμάτων;**
	☐ Καθόλου ☐ Λίγο ☐ Μέτρια ☐ Πολύ ☐ Πάρα πολύ
**10**	**Η σύνθεση του μηνιαίου διαιτολογίου είναι συνολικά ισορροπημένη;**
	☐ Καθόλου ☐ Λίγο ☐ Μέτρια ☐ Πολύ ☐ Πάρα πολύ
**11**	**Το μηνιαίο διαιτολόγιο είναι διατροφικά επαρκές ως πρότυπο ημερήσιο πρόγραμμα;**
	☐ Καθόλου ☐ Λίγο ☐ Μέτρια ☐ Πολύ ☐ Πάρα πολύ
**12**	**Το μηνιαίο διαιτολόγιο είναι ρεαλιστικό και εφαρμόσιμο στην καθημερινή πράξη;**
	☐ Καθόλου ☐ Λίγο ☐ Μέτρια ☐ Πολύ ☐ Πάρα πολύ
**13**	**Οι επιλογές τροφίμων και γευμάτων είναι σαφείς και εύχρηστες;**
	☐ Καθόλου ☐ Λίγο ☐ Μέτρια ☐ Πολύ ☐ Πάρα πολύ
**14**	**Το διαιτολόγιο είναι τέτοιο που θα μπορούσε να αξιοποιηθεί στην επαγγελματική πρακτική;**
	☐ Καθόλου ☐ Λίγο ☐ Μέτρια ☐ Πολύ ☐ Πάρα πολύ
**15**	**Θα δίνατε ένα παρόμοιο διαιτολόγιο σε διαιτώμενό/ή σας, με μικρές ή χωρίς αλλαγές.**
	☐ Καθόλου ☐ Λίγο ☐ Μέτρια ☐ Πολύ ☐ Πάρα πολύ
**Συνταγές**	
**13**	**Οι συνταγές είναι συμβατές με τις αρχές της Μεσογειακής διατροφής.**
	☐ Καθόλου ☐ Λίγο ☐ Μέτρια ☐ Πολύ ☐ Πάρα πολύ
**14**	**Οι συνταγές είναι απλές και πρακτικές στην εκτέλεση.**
	☐ Καθόλου ☐ Λίγο ☐ Μέτρια ☐ Πολύ ☐ Πάρα πολύ
**15**	**Οι συνταγές χρησιμοποιούν υλικά εύκολα διαθέσιμα.**
	☐ Καθόλου ☐ Λίγο ☐ Μέτρια ☐ Πολύ ☐ Πάρα πολύ
**16**	**Οι συνταγές είναι δημιουργικές/ευφάνταστες.**
	☐ Καθόλου ☐ Λίγο ☐ Μέτρια ☐ Πολύ ☐ Πάρα πολύ
**17**	**Οι συνταγές ταιριάζουν ουσιαστικά με το ύφος και τη λογική του διαιτολογίου;**
	☐ Καθόλου ☐ Λίγο ☐ Μέτρια ☐ Πολύ ☐ Πάρα πολύ
**18**	**Κατά πόσο οι συνταγές είναι κατάλληλες για εφαρμογή στον ελληνικό/μεσογειακό πληθυσμό, λαμβάνοντας υπόψη διατροφικές συνήθειες και κουλτούρα;**
	☐ Καθόλου ☐ Λίγο ☐ Μέτρια ☐ Πολύ ☐ Πάρα πολύ
**20**	**Κατά πόσο οι συνταγές θα μπορούσαν να εφαρμοστούν σε πληθυσμούς εκτός Μεσογείου (π.χ. Β. Ευρώπη ή ΗΠΑ), λαμβάνοντας υπόψη διαθεσιμότητα τροφίμων και διατροφικές συνήθειες;**
	☐ Καθόλου ☐ Λίγο ☐ Μέτρια ☐ Πολύ ☐ Πάρα πολύ

Συνολική αξιολόγηση	
**21**	**Συνολικά, το διαιτολόγιο είναι ποιοτικό ως παραγόμενο αποτέλεσμα AI;**
	☐ Καθόλου ☐ Λίγο ☐ Μέτρια ☐ Πολύ ☐ Πάρα πολύ
**22**	**Συνολικά, οι συνταγές προσθέτουν αξία στο διαιτολόγιο;**
	☐ Καθόλου ☐ Λίγο ☐ Μέτρια ☐ Πολύ ☐ Πάρα πολύ
**23**	**Συνολικά, η εφαρμογή παράγει διαιτολόγια και συνταγές επαγγελματικά αξιοποιήσιμα;**
	☐ Καθόλου ☐ Λίγο ☐ Μέτρια ☐ Πολύ ☐ Πάρα πολύ
**24**	**Τα αρχεία ανάλυσης αποτυπώνουν επαρκώς κατά πόσο το διαιτολόγιο είναι συμβατό με τις αρχές της Μεσογειακής διατροφής.**
	☐ Καθόλου ☐ Λίγο ☐ Μέτρια ☐ Πολύ ☐ Πάρα πολύ
**25**	**Η παρουσίαση και η ανάλυση των μακροθρεπτικών συστατικών είναι επαρκής για να κριθεί αν το διαιτολόγιο ευθυγραμμίζεται με τις διατροφικές κατευθυντήριες οδηγίες.**
	☐ Καθόλου ☐ Λίγο ☐ Μέτρια ☐ Πολύ ☐ Πάρα πολύ
**26**	**Η παρουσίαση και η ανάλυση των μικροθρεπτικών συστατικών είναι επαρκής για να κριθεί η διατροφική επάρκεια του διαιτολογίου.**
	☐ Καθόλου ☐ Λίγο ☐ Μέτρια ☐ Πολύ ☐ Πάρα πολύ
**27**	**Η διάκριση μεταξύ αρχείου για διαιτώμενο/-η και αρχείου για επαγγελματία είναι ουσιαστική και εξυπηρετεί αποτελεσματικά τις ανάγκες κάθε χρήστη.**
	☐ Καθόλου ☐ Λίγο ☐ Μέτρια ☐ Πολύ ☐ Πάρα πολύ
**28**	**Η οπτική παρουσίαση των στοιχείων ανάλυσης (π.χ. πίνακες, γραφήματα, χρωματική επισήμανση) πιστεύετε ότι ενισχύει τη χρησιμότητα των αρχείων;**
	☐ Καθόλου ☐ Λίγο ☐ Μέτρια ☐ Πολύ ☐ Πάρα πολύ

Supplementary Table S7. Comparison of nutrient goals with MAIDIET-generated daily nutrient outputs across six energy-level meal plans.

Dietary plan	Nutrients	Nutrient goal	MAI-DIET output (mean ± SD/day)	Nutrient status
**1600 kcal**	Energy (kcal/day)	1600 (1408–1792)	1627.7 ± 33.9	Criteria met
	Carbohydrates (g/day)	180–220 (45–55 % E)	182.7 ± 22.5	Criteria met
	Protein (g/day)	60–80 (15–20 % E)	85.9 ± 18.2	+7.4 %; Slightly above the predefined target, but unlikely to be clinically meaningful [49-51]
	Fat (g/day)	53–71 (30–40 % E)	63.5 ± 8.2	Criteria met
	MUFA (% fat)	MUFA-dominant pattern	52 % of fat	Criteria met
	Marine n-3 (g/day)	≥ 0.25 g/day (EFSA)	0.49 ± 0.72	Criteria met
	Vitamin C (mg/day)	95 (EFSA PRI, F)	133.8 ± 104.7	Criteria met
	Vitamin B12 (µg/day)	4.0 (EFSA AI)	7.4 ± 9.3	Criteria met
	Folate (µg/day)	330 (EFSA PRI)	342.3 ± 99.0	Criteria met
	Iron (mg/day)	16 (EFSA PRI, F)	16.4 ± 4.7	Criteria met
	Calcium (mg/day)	950 (EFSA PRI)	783.5 ± 187.9	-17.5 %; Criteria not met
	Fiber (g/day)	≥ 25 g/day (EFSA AI)	26.3 ± 6.7 g/day	Criteria met
	Sodium (mg/day)	WHO < 2000; AHA ≤2300	1878.0 ± 357.8 mg/day	Criteria met
**1800 kcal**	Energy (kcal/day)	1800 (1566–1926)	1822.4 ± 37.9	Criteria met
	Carbohydrates (g/day)	202–248 (45–55 % E)	203.2 ± 23.4	Criteria met
	Protein (g/day)	68–90 (15–20 % E)	96.8 ± 18.8	+7.5 %; Slightly above the predefined target, but unlikely to be clinically meaningful [49-51]
	Fat (g/day)	60–80 (30–40 % E)	71.5 ± 5.8	Criteria met
	MUFA (% fat)	MUFA-dominant pattern	51 % of fat	Criteria met
	Marine n-3 (g/day)	≥ 0.25 g/day (EFSA)	0.55 ± 0.81	Criteria met
	Vitamin C (mg/day)	95 (EFSA PRI, F)	133.4 ± 95.2	Criteria met
	Vitamin B12 (µg/day)	4.0 (EFSA AI)	8.4 ± 10.4	Criteria met
	Folate (µg/day)	330 (EFSA PRI)	387.4 ± 118.1	Criteria met
	Iron (mg/day)	16 (EFSA PRI, F)	18.7 ± 5.2	Criteria met
	Calcium (mg/day)	950 (EFSA PRI)	946.4 ± 315.6	-0.4 %; Borderline criteria met
	Fiber (g/day)	≥ 25 g/day (EFSA AI)	28.3 ± 5.3 g/day	Criteria met
	Sodium (mg/day)	WHO < 2000; AHA ≤2300	2318.2 ± 515.2 mg/day	+15.9 %; Criteria not met
**2000 kcal**	Energy (kcal/day)	2000 (1740–2140)	2032.6 ± 46.3	Criteria met
	Carbohydrates (g/day)	225–275 (45–55 % E)	224.2 ± 25.0	Criteria met
	Protein (g/day)	75–100 (15–20 % E)	106.7 ± 25.3	+6.7 %; Slightly above the predefined target, but unlikely to be clinically meaningful [49-51]
	Fat (g/day)	67–89 (30–40 % E)	81.2 ± 9.6	Criteria met
	MUFA (% fat)	MUFA-dominant pattern	51 % of fat	Criteria met
	Marine n-3 (g/day)	≥ 0.25 g/day (EFSA)	0.62 ± 0.89	Criteria met
	Vitamin C (mg/day)	110 (EFSA PRI, M)	143.6 ± 140.7	Criteria met
	Vitamin B12 (µg/day)	4.0 (EFSA AI)	9.5 ± 11.7	Criteria met

	Folate (µg/day)	330 (EFSA PRI)	450.6 ± 149.4	Criteria met
	Iron (mg/day)	11 (EFSA PRI, M)	20.7 ± 5.7	Criteria met
	Calcium (mg/day)	950 (EFSA PRI)	1048.6 ± 315.4	Criteria met
	Fiber (g/day)	≥ 25 g/day (EFSA AI)	31.3 ± 6.6 g/day	Criteria met
	Sodium (mg/day)	WHO < 2000; AHA ≤2300	2555.9 ± 609.1 mg/day	+11.0 %; Criteria not met
**2200 kcal**	Energy (kcal/day)	2200 (1936–2464)	2234.4 ± 50.1	Criteria met
	Carbohydrates (g/day)	248–302 (45–55 % E)	243.2 ± 33.7	-1.9 %; Slightly below the predefined target, but unlikely to be clinically meaningful [52]
	Protein (g/day)	82–110 (15–20 % E)	117.0 ± 29.1	+6.4 %; Slightly above the predefined target, but unlikely to be clinically meaningful [49-51]
	Fat (g/day)	73–98 (30–40 % E)	90.6 ± 10.1	Criteria met
	MUFA (% fat)	MUFA-dominant pattern	51 % of fat	Criteria met
	Marine n-3 (g/day)	≥ 0.25 g/day (EFSA)	0.83 ± 1.01	Criteria met
	Vitamin C (mg/day)	95 (EFSA PRI, F)	122.1 ± 80.6	Criteria met
	Vitamin B12 (µg/day)	4.0 (EFSA AI)	10.2 ± 11.2	Criteria met
	Folate (µg/day)	330 (EFSA PRI)	471.9 ± 133.3	Criteria met
	Iron (mg/day)	16 (EFSA PRI, F)	23.4 ± 6.8	Criteria met
	Calcium (mg/day)	950 (EFSA PRI)	1195.2 ± 355.8	Criteria met
	Fiber (g/day)	≥ 25 g/day (EFSA AI)	33.6 ± 7.5 g/day	Criteria met
	Sodium (mg/day)	WHO < 2000; AHA ≤2300	2873.0 ± 612.0 mg/day	+24.9 %; Criteria not met
**2400 kcal**	Energy (kcal/day)	2400 (2112–2688)	2420.0 ± 53.7	Criteria met
	Carbohydrates (g/day)	270–330 (45–55 % E)	267.5 ± 32.9	-0.9 %; Borderline criteria met [52]
	Protein (g/day)	90–120 (15–20 % E)	126.6 ± 30.2	+5.5 %; Slightly above the predefined target, but unlikely to be clinically meaningful [49-51]
	Fat (g/day)	80–107 (30–40 % E)	96.0 ± 10.2	Criteria met
	MUFA (% fat)	MUFA-dominant pattern	51 % of fat	Criteria met
	Marine n-3 (g/day)	≥ 0.25 g/day (EFSA)	0.79 ± 1.04	Criteria met
	Vitamin C (mg/day)	95 (EFSA PRI, F)	126.0 ± 96.6	Criteria met
	Vitamin B12 (µg/day)	4.0 (EFSA AI)	11.4 ± 12.8	Criteria met
	Folate (µg/day)	330 (EFSA PRI)	481.8 ± 88.1	Criteria met
	Iron (mg/day)	16 (EFSA PRI, F)	25.8 ± 8.4	Criteria met
	Calcium (mg/day)	950 (EFSA PRI)	1213.1 ± 340.7	Criteria met
	Fiber (g/day)	≥ 25 g/day (EFSA AI)	36.5 ± 6.3 g/day	Criteria met
	Sodium (mg/day)	WHO < 2000; AHA ≤2300	2955.1 ± 616.3 mg/day	+28.5 %; Criteria not met
**2600 kcal**	Energy (kcal/day)	2600 (2288–2912)	2632.0 ± 62.3	Criteria met
	Carbohydrates (g/day)	292–358 (45–55 % E)	285.1 ± 41.6	-2.4 %; Slightly below the predefined target, but unlikely to be clinically meaningful [52]
	Protein (g/day)	98–130 (15–20 % E)	139.2 ± 35.3	+7.1 %; Slightly above the predefined target, but unlikely to be clinically meaningful [49-51]

	Fat (g/day)	87–116 (30–40 % E)	106.5 ± 11.3	Criteria met
	MUFA (% fat)	MUFA-dominant pattern	50 % of fat	Criteria met
	Marine n-3 (g/day)	≥ 0.25 g/day (EFSA)	1.11 ± 1.26	Criteria met
	Vitamin C (mg/day)	110 (EFSA PRI, M)	155.2 ± 145.6	Criteria met
	Vitamin B12 (μg/day)	4.0 (EFSA AI)	12.4 ± 11.4	Criteria met
	Folate (μg/day)	330 (EFSA PRI)	528.3 ± 152.9	Criteria met
	Iron (mg/day)	11 (EFSA PRI, M)	26.8 ± 7.2	Criteria met
	Calcium (mg/day)	950 (EFSA PRI)	1402.9 ± 467.5	Criteria met
	Fiber (g/day)	≥ 25 g/day (EFSA AI)	40.0 ± 8.4 g/day	Criteria met
	Sodium (mg/day)	WHO < 2000; AHA ≤2300	3135.8 ± 751.1 mg/day	+36.3; Criteria not met

Abbreviations: MUFA, monounsaturated fatty acids; % E, percentage of total energy intake; n-3, omega-3 fatty acids; PRI, Population Reference Intake (EFSA); AI, Adequate Intake (EFSA); EFSA, European Food Safety Authority; WHO, World Health Organization; AHA, American Heart Association; SD, standard deviation; F, female; M, male.

Supplementary Table S8. Day-by-day energy and macronutrient output for the 2000 kcal plan (Case 3, male, 28 days), with comparison between the user-facing summary report and the healthcare-professional-facing deep analysis report.

Day	Energy (kcal)			Protein (g)			Carbohydrates (g)			Fat (g)		
	User summary	Deep analysis	Δ (g)	User summary	Deep analysis	Δ (g)	User summary	Deep analysis	Δ (g)	User summary	Deep analysis	Δ (g)
**1**	2100	2100	0.0	95.8	95.8	0.0	241.4	241.4	0.0	87.3	87.3	0.0
**2**	2075	2075	0.0	142.3	142.3	0.0	223.0	223.0	0.0	73.0	73.0	0.0
**3**	2070	2070	0.0	115.1	115.1	0.0	224.8	224.8	0.0	80.8	80.8	0.0
**4**	2093	2093	0.0	140.6	140.6	0.0	210.7	210.7	0.0	80.1	80.1	0.0
**5**	2078	2078	0.0	110.4	110.4	0.0	218.9	218.9	0.0	86.0	86.0	0.0
**6**	2088	2088	0.0	81.9	81.9	0.0	266.4	266.4	0.0	82.2	82.2	0.0
**7**	2020	2020	0.0	103.7	103.7	0.0	219.2	219.2	0.0	82.6	82.6	0.0
**8**	1951	1951	0.0	87.8	87.8	0.0	223.4	223.4	0.0	82.3	82.3	0.0
**9**	2100	2100	0.0	113.5	113.5	0.0	230.1	230.1	0.0	83.1	83.1	0.0
**10**	2025	2025	0.0	156.5	156.5	0.0	181.3	181.3	0.0	76.9	76.9	0.0
**11**	1982	1982	0.0	105.9	105.9	0.0	263.6	263.6	0.0	62.9	62.9	0.0
**12**	1943	1943	0.0	99.8	99.8	0.0	232.9	232.9	0.0	68.4	68.4	0.0
**13**	2000	2000	0.0	98.2	98.2	0.0	253.5	253.5	0.0	69.0	69.0	0.0
**14**	2000	2000	0.0	54.3	54.3	0.0	217.8	217.8	0.0	104.3	104.3	0.0
**15**	2000	2000	0.0	119.9	119.9	0.0	217.0	217.0	0.0	74.3	74.3	0.0
**16**	2078	2078	0.0	128.6	128.6	0.0	200.9	200.9	0.0	86.5	86.5	0.0
**17**	2032	2032	0.0	159.8	159.8	0.0	160.1	160.1	0.0	84.3	84.3	0.0
**18**	2029	2029	0.0	95.5	95.5	0.0	230.5	230.5	0.0	80.6	80.6	0.0
**19**	2000	2000	0.0	88.1	88.1	0.0	270.3	270.3	0.0	65.3	65.3	0.0
**20**	2000	2000	0.0	77.7	77.7	0.0	270.0	270.0	0.0	73.9	73.9	0.0
**21**	2000	2000	0.0	77.1	77.1	0.0	202.3	202.3	0.0	100.3	100.3	0.0
**22**	2088	2088	0.0	107.0	107.0	0.0	218.3	218.3	0.0	89.2	89.2	0.0
**23**	2057	2057	0.0	120.5	120.5	0.0	215.6	215.6	0.0	78.9	78.9	0.0
**24**	2039	2039	0.0	122.5	122.5	0.0	210.6	210.6	0.0	80.7	80.7	0.0
**25**	2000	2000	0.0	120.2	120.2	0.0	218.9	218.9	0.0	75.2	75.2	0.0
**26**	2065	2065	0.0	89.7	89.7	0.0	227.6	227.6	0.0	90.7	90.7	0.0
**27**	2038	2038	0.0	108.5	108.5	0.0	224.8	224.8	0.0	79.2	79.2	0.0
**28**	1962	1962	0.0	66.0	66.0	0.0	204.6	204.6	0.0	96.0	96.0	0.0
mean ± SD	*2032.6 ± 46.3*	*2032.6 ± 46.3*	*0.0*	*106.7 ± 25.3*	*106.7 ± 25.3*	*0.0*	*224.2 ± 25.0*	*224.2 ± 25.0*	*0.0*	*81.2 ± 9.6*	*81.2 ± 9.6*	*0.0*

User summary: daily values are extracted from the per-day nutrition tables in the summary report and rounded to one decimal place. Deep analysis: values are derived from the identical recipe nutrient database (MedAI_recipe_nutrition_v9_0.csv) via the same aggregation function as the summary report; macronutrient results are rendered as charts in the deep analysis report, with underlying values sharing the same computation as the user summary. Δ: absolute deviation between the two outputs. Over the 28-day plan period, the expected cumulative absolute deviation per macronutrient is $\sqrt{28} \times 0.025 \approx 0.13$ g.

Supplementary Table S9. Consolidated technical performance metrics of the MAI-DIET framework.

Domain	Metric	Result
Nutrient calculation accuracy	Independent recomputation of stored recipe nutrient values (25 randomly sampled recipes; sampling seed 42; energy, protein, carbohydrate and fat)	100 of 100 field comparisons matched within rounding
	Mean absolute difference across all compared fields	0.0012%
	Mean absolute difference, energy	0.0022 kcal (maximum 0.0050 kcal)
	Recipes differing by more than 1%	0
Planning success rate	Days within the predefined daily energy tolerance band, per plan	28 of 28 days in each of the six plans
Data quality	Recipe nutrient confidence tier (252 entries: 239 recipes and 13 side dishes)	High 200 (79.4%); medium 34 (13.5%); low 18 (7.1%)
	Entries admitted to the recipe selection pools	234 of 252; low-confidence entries excluded
	Ingredient-to-food resolution table	400 entries: 275 exact, 112 contained, 11 no match, 2 excluded
Computational efficiency	Generation of one complete 28-day plan, excluding document export	1.5–1.8 s wall-clock time
	Hardware and interpreter used for timing	AMD Ryzen 3 3200G, 4 logical CPUs; Python 3.14.0

The recomputation was performed with a script written independently of the production nutrient pipeline, operating on ingredient masses, per-100 g values in the standardized food-composition library, and the applicable cooking-retention factors. It verifies the internal arithmetic of the pipeline and is not an audit of the underlying reference nutrient values, which are taken unchanged from public food-composition sources.

Supplementary Table S10. Seed-sensitivity analysis of generated plan outcomes across five seed sequences.

Outcome	1600 kcal	1800 kcal	2000 kcal	2200 kcal	2400 kcal	2600 kcal	Maximum
Mean daily energy (kcal)	1.1	1.4	1.1	2.3	1.0	3.2	3.2
Protein (% of energy)	1.5	2.5	2.0	4.4	2.5	3.4	4.4
Carbohydrate (% of energy)	2.6	2.4	3.6	3.8	3.1	2.7	3.8
Fat (% of energy)	3.1	2.3	4.1	4.1	3.8	3.7	4.1
Sodium (mg)	4.5	5.7	4.4	5.2	3.2	8.5	8.5
Calcium (mg)	4.2	6.0	5.5	3.6	4.1	3.0	6.0
Iron (mg)	2.6	7.0	6.2	5.9	4.0	3.8	7.0
Fiber (g)	4.2	4.4	2.9	5.6	4.1	4.7	5.6
Folate (µg)	5.1	8.3	12.3	9.0	8.9	8.4	12.3
Vitamin B12 (µg)	2.3	12.9	4.6	9.8	7.0	6.0	12.9
Vitamin C (mg)	15.3	4.0	12.4	19.4	17.4	8.3	19.4
Marine n-3 fatty acids (g)	13.8	19.6	13.7	17.9	14.3	11.0	19.6

Values are the relative range across the five seed sequences, expressed as a percentage of the mean (maximum minus minimum, divided by the mean). Column headings denote the daily energy level of the plan. Each of the six profiles was regenerated under five seed sequences: the sequence used for the evaluated plans, [42, 123, 456, 789], and four further sequences drawn programmatically from a recorded meta-seed of 2026, giving 30 plans in total. The drawn sequences were [15614, 41876, 65866, 67086], [84830, 13453, 29267, 78779], [81460, 72950, 55131, 75048] and [71803, 95754, 64344, 98439]; the identical set can be redrawn from the stated meta-seed. Five seed sequences across six profiles is a small sample and the ranges reported are indicative rather than confidence intervals. The three diet-quality indices (MedDietScore, Dietary Phytochemical Index, GR-UPFAST) could not be included because they are scored manually and no automated implementation exists. Where nutrient-source data were missing, values were stored as zero; therefore, the reported micronutrient estimates may represent lower-bound estimates.

Supplementary Table S11. Participant characteristics, AI experience, digital application use and assigned meal-plan energy level among health professionals (N = 103).

**Daily energy content of the evaluated meal plan, n (%)**	
1600 kcal/day	15 (14.6)
1800 kcal/day	25 (24.3)
2000 kcal/day	19 (18.4)
2200 kcal/day	16 (15.5)
2400 kcal/day	15 (14.6)
2600 kcal/day	13 (12.6)
**Sex, n (%)**	
Females	74 (71.8)
Males	29 (28.2)
**Frequency of digital application use, n (%)**	
Never	1 (1.0)
Rarely	3 (2.9)
Occasionally	22 (21.4)
Several times per week	37 (35.9)
Daily	40 (38.8)
**Previous use of AI tools, n (%)**	
Yes	81 (78.6)
No	22 (21.4)

Values are presented as frequencies and percentages. AI: artificial intelligence.

Supplementary Table S12. Item-level professional evaluation of the MAIDIET generated monthly meal plan and recipes.

**Domain 1: Nutritional adequacy and structure of the diet plan**			
Item	Aim of each item	Median (IQR)	Positive responses, n (%)
1.1	Energy target adequacy	4.0 (4.0 – 4.0)	84 (81.6)
1.2	Balanced energy distribution	4.0 (4.0 – 4.0)	82 (79.6)
1.3	Inclusion of basic food groups	4.0 (4.0 – 5.0)	83 (80.6)
1.4	Food and meal variety	4.0 (3.0 – 4.0)	77 (74.8)
1.5	Overall meal-plan balance	4.0 (4.0 – 4.0)	72 (69.9)
1.6	Nutritional adequacy	4.0 (3.0 – 4.0)	79 (76.7)
**Domain 2: Practical and professional applicability of the diet plan**			
Item	Aim of each item	Median (IQR)	Positive responses, n (%)
2.1	Real life applicability	4.0 (3.0 – 4.0)	56 (54.4)
2.2	Clarity of food/meal options	4.0 (3.0 – 4.0)	76 (73.8)
2.3	Use in professional practice	4.0 (3.0 – 4.0)	59 (57.3)
2.4	Willingness to provide to clients	4.0 (3.0 – 4.0)	65 (63.1)
2.5	Overall meal-plan quality	4.0 (3.0 – 4.0)	74 (71.8)
**Domain 3: Recipe quality and Mediterranean compatibility**			
Item	Aim of each item	Median (IQR)	Positive responses, n (%)
3.1	Mediterranean diet compatibility	4.0 (4.0 – 5.0)	89 (86.4)
3.2	Recipe simplicity/practicality	4.0 (3.0 – 4.0)	74 (71.8)
3.3	Ingredient availability	4.0 (3.75 – 4.0)	78 (75.7)
3.4	Recipe creativity	4.0 (3.0 – 4.0)	65 (63.1)
3.5	Alignment with meal-plan rationale	4.0 (4.0 – 4.0)	87 (84.5)
3.6	Suitability for Greek/Mediterranean populations	4.0 (3.0 – 4.0)	66 (64.1)
3.7	Added value of recipes	4.0 (4.0 – 4.0)	83 (80.6)
3.8	Usability of outputs	4.0 (3.0 – 4.0)	59 (57.3)
**Domain 4: Documentation, nutritional analysis and audit-file quality**			
Item	Aim of each item	Median (IQR)	Positive responses, n (%)
4.1	Documentation of Mediterranean diet consistency	4.0 (3.0 – 4.0)	77 (74.8)
4.2	Macronutrient analysis adequacy	4.0 (4.0 – 5.0)	80 (77.7)
4.3	Micronutrient analysis adequacy	4.0 (4.0 – 4.25)	88 (85.4)
4.4	User-specific file distinction	4.0 (3.0 – 4.25)	76 (73.8)
4.5	Usefulness of visual presentation	4.0 (4.0 – 5.0)	81 (78.6)
**Perceived generalizability**			
Adaptability to non-Mediterranean populations		Median (IQR)	Positive responses, n (%)
		3.0 (3.0 – 4.0)	40 (38.8)

Positive responses were defined as ratings of 4 or 5 (i.e., “Very” or “Very much”). Individual Likert-type items were treated as ordinal variables.

Supplementary Table S13. Health professional evaluation scores according to the energy level of the evaluated meal plan.

	**1600 kcal/day (n = 15)**	**1800 kcal/day (n=25)**	**2000 kcal/day (n=19)**	**2200 kcal/day (n=16)**	**2400 kcal/day (n=15)**	**2600 kcal/day (n=13)**	***p-value***
Domain 1 Score	3.92 (3.5 – 4.2)	4.0 (3.7 – 4.5)	4.0 (3.5 – 4.5)	3.83 (3.0 – 4.2)	4.17 (4.0 – 4.5)	3.83 (3.3 – 4.2)	0.173
Domain 2 Score	4.0 (3.8 – 4.3)	3.8 (3.1 – 4.4)	3.3 (2.8 – 4.0)	3.6 (3.0 – 3.6)	4.0 (3.9 – 4.2)	3.4 (2.8 – 4.0)	0.053
Domain 3 Score	4.06 (3.6 – 4.5)	3.87 (3.5 – 4.3)	3.81 (3.7 – 4.0)	3.75 (3.0 – 4.0)	4.06 (3.8 – 4.4)	4.0 (3.4 – 4.3)	0.456
Domain 4 Score	4.1 (3.8 – 4.4)	3.9 (3.7 – 4.6)	4.1 (4.0 – 4.9)	3.8 (3.0 – 4.2)	4.3 (3.7 – 4.7)	3.8 (3.5 – 4.4)	0.06
Overall acceptability	4.04 (3.7 – 4.2)	3.83 (3.6 – 4.3)	3.88 (3.5 – 4.1)	3.71 (3.0 – 4.0)	4.2 (3.8 – 4.5)	3.67 (3.3 – 4.2)	0.190

Values are presented as median and interquartile range (IR). p-values derived from Kruskal–Wallis tests.
