# Supplementary material for: Clinician–Artificial Intelligence Collaboration for Mediterranean Meal-Plan Generation: Development, Technical Feasibility, and Professional Acceptability of the MAI-DIET Framework
Source: Nutrients. 2026 Aug 14;18(16):2661. doi: 10.3390/nu18162661 (PMC13516381; doi:10.3390/nu18162661)
Supplement: Supplementary file 1 [file nutrients-18-02661-s001.zip › nutrients-4447558_MAI-DIET_R1_Supplementary File S2.pdf]

Supplementary File S2

Persephone Deterministic Engine versus Direct Large-Language-Model Generation: Six Matched 28-Day Mediterranean Meal Plans

Companion analysis to: Clinician–Artificial Intelligence Collaboration for Mediterranean Meal-Plan Generation — Development, Technical Feasibility, and Professional Acceptability of the MAI-DIET Framework

Contents

Purpose and Method
Summary Across the Six Plans (**Table S1, Figure S1**)
Plan 1 — 1600 kcal/day: 28-day plan, comparison with the MAI-DIET plan, compliance heat map (**Figure S2**)
Plan 2 — 1800 kcal/day: 28-day plan, comparison with the MAI-DIET plan, compliance heat map (**Figure S3**)
Plan 3 — 2000 kcal/day: 28-day plan, comparison with the MAI-DIET plan, compliance heat map (**Figure S4**)
Plan 4 — 2200 kcal/day: 28-day plan, comparison with the MAI-DIET plan, compliance heat map (**Figure S5**)
Plan 5 — 2400 kcal/day: 28-day plan, comparison with the MAI-DIET plan, compliance heat map (**Figure S6**)
Plan 6 — 2600 kcal/day: 28-day plan, comparison with the MAI-DIET plan, compliance heat map (**Figure S7**)
Error Analysis of the Six Matched Plan Sets (**Table S2**)
Interpretation
Appendix. The Comparator Prompt as Issued (**Table S3**)

1. Purpose and Method

This report presents six 28-day Mediterranean meal plans generated directly by Claude Sonnet 5 (medium effort), a general-purpose large language model for the same six hypothetical adult profiles used in the main study. The plans were produced from a single specification given in one prompt per profile, stating the profile characteristics, the target daily energy, the meal structure of three meals and two snacks, a Mediterranean dietary pattern, and a requirement that every ingredient carry an explicit quantity in grams or milliliters. No nutrient reference values, tolerance bands, or compliance criteria were supplied to the model, so that the request would resemble typical use from the general population rather than a specification of the answer.

Each plan is reported in full below, followed by a short comparison against the corresponding MAI-DIET plan and a compliance heat map in the format used in the main manuscript. Nutrient values shown for the language-model plans are those reported by the model itself. Compliance status was assigned afterwards by applying the same reference criteria used for the MAI-DIET plans in **Supplementary Table S7**, so that both systems are judged against an identical rubric.

The comparison concerns direct language-model generation only. It does not constitute a comparison against dietitian-designed plans or against commercial nutrition software, both of which remain defined objectives of a separate study.

---

2. Summary Across the Six Plans

Table S1. Energy outcome and criteria compliance for both systems across the six matched profiles. Criteria are provided in **Supplementary Table S7** of the main manuscript. Unique dishes are counted across the 140 meal slots of each 28-day plan.

Plan	Target (kcal/d)	MAI-DIET mean ± SD	Claude mean ± SD	MAI-DIET criteria met	Claude criteria met	Unique dishes
1	1600	1627.7 ± 33.9	1595.0 ± 98.4	10/13	8/13	58
2	1800	1822.4 ± 37.9	1798.4 ± 23.0	9/13	9/13	60

3	2000	2032.6 ± 46.3	2004.5 ± 131.3	10/13	10/13	68
4	2200	2234.4 ± 50.1	2208.2 ± 183.9	10/13	10/13	101
5	2400	2420.0 ± 53.7	2399.8 ± 167.9	10/13	11/13	46
6	2600	2632.0 ± 62.3	2574.1 ± 90.0	10/13	11/13	75

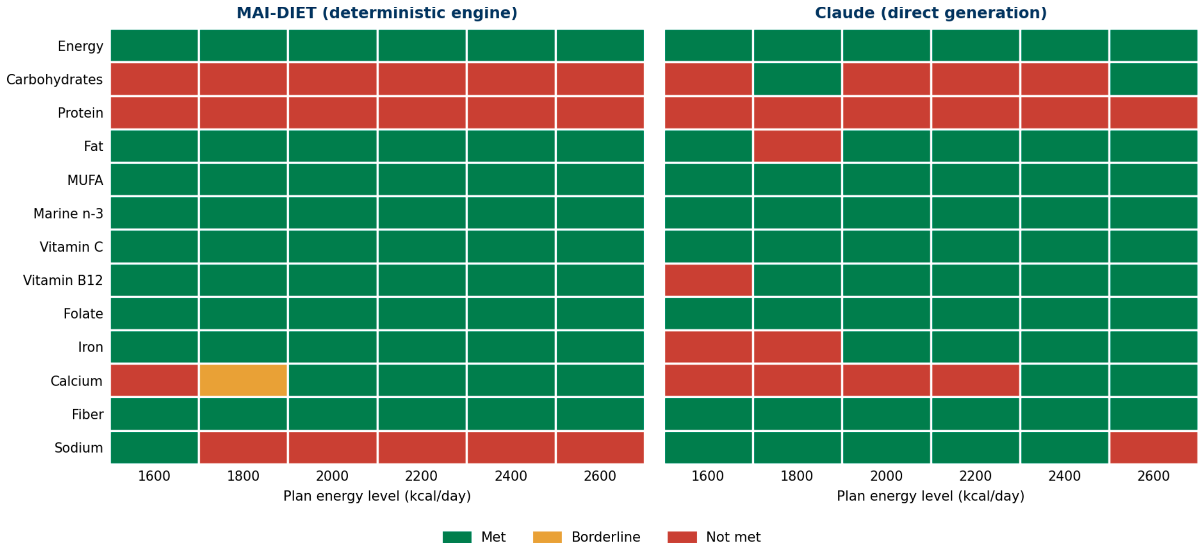

Figure S1. Side-by-side compliance heat maps for the six matched plans. Left: MAI-DIET deterministic engine. Right: direct language-model generation. Both panels apply the same thirteen reference criteria.

Across the six profiles the deterministic engine met the reference criteria in 10 to 11 instances of thirteen, with a consistent pattern of shortfall: carbohydrate contribution below the reference range, protein above it, and sodium above the WHO target at the higher energy levels. The language-model plans met between 8 and 11 criteria, and the pattern of shortfall varied between profiles rather than remaining constant. The language-model plans were consistently lower in sodium, which is an advantage over the engine at the higher energy levels, and were more frequently below the calcium reference intake.

The difference of principal interest is not the count of criteria met but the consistency of behavior. Mean daily energy in the language-model plans fell within one per cent of the requested target in every profile, while the standard deviation of daily energy ranged from 23.0 to 183.9 kcal. The corresponding range for the deterministic engine was 33.9 to 62.3 kcal. The provenance of the reported nutrient values is examined in the error analysis below.

3. Plan 1 — 1600 kcal/day

**Sex**	Female	**Age**	35 y	**Height**	155 cm
**Body mass**	50 kg	**Activity**	1.375 (lightly active)	**Goal**	Weight maintenance

3.1. Twenty-eight-day meal plan

Day 1

Breakfast. *Greek Yogurt with Honey, Walnuts and Strawberries* — Greek yogurt (2% fat) — 175 g; Honey — 17 g; Walnuts — 17 g; Strawberries — 93 g

Snack 1. *Almonds and Orange* — Almonds — 17 g; Orange — 117 g

Lunch. *Greek Salad with Chickpeas and Bread* — Tomato — 175 g; Cucumber — 117 g; Bell pepper — 70 g; Kalamata olives — 23 g; Feta cheese — 47 g; Extra virgin olive oil — 17 ml; Chickpeas (cooked) — 117 g; Whole wheat bread — 35 g **Snack 2.** *Chia Pudding with Honey* — Chia seeds — 17 g; Milk (1.5% fat) — 117 ml; Honey — 6 g

Dinner. *Shrimp Saganaki (Tomato and Feta)* — Shrimp — 140 g; Tomato — 175 g; Feta cheese — 47 g; Extra virgin olive oil — 12 ml; Whole wheat bread — 35 g

Day 2

Breakfast. *Whole Wheat Toast with Feta and Tomato* — Whole wheat bread — 70 g; Feta cheese — 35 g; Tomato — 93 g; Extra virgin olive oil — 6 ml

Snack 1. *Feta and Grapes* — Feta cheese — 35 g; Grapes — 93 g

Lunch. *Lentil Soup (Fakes)* — Lentils (cooked) — 292 g; Onion — 47 g; Carrot — 47 g; Tomato — 70 g; Extra virgin olive oil — 14 ml; Whole wheat bread — 47 g

Snack 2. *Grapes and Walnuts* — Grapes — 117 g; Walnuts — 12 g

Dinner. *Vegetable and Chickpea Soup* — Chickpeas (cooked) — 175 g; Carrot — 47 g; Celery — 35 g; Tomato — 93 g; Extra virgin olive oil — 12 ml

Day 3

Breakfast. *Vegetable Omelette with Toast* — Egg — 117 g; Extra virgin olive oil — 6 ml; Spinach — 47 g; Tomato — 47 g; Whole wheat bread — 35 g

Snack 1. *Barley Rusk with Honey* — Barley rusk (paximadi) — 29 g; Honey — 12 g

Lunch. *Grilled Chicken with Bulgur Pilaf and Salad* — Chicken breast — 140 g; Bulgur (cooked) — 175 g; Extra virgin olive oil — 12 ml; Tomato — 93 g; Cucumber — 70 g; Lemon juice — 5 ml

Snack 2. *Banana with Tahini* — Banana — 117 g; Tahini — 12 g

Dinner. *Grilled Salmon with Greek Salad* — Salmon fillet — 117 g; Tomato — 117 g; Cucumber — 93 g; Kalamata olives — 17 g; Feta cheese — 29 g; Extra virgin olive oil — 12 ml

Day 4

Breakfast. *Overnight Oats with Banana and Chia* — Rolled oats — 47 g; Milk (1.5% fat) — 175 ml; Chia seeds — 9 g; Banana — 70 g; Honey — 6 g

Snack 1. *Fresh Watermelon Slices* — Watermelon — 175 g

Lunch. *Baked Salmon with Quinoa and Zucchini* — Salmon fillet — 140 g; Quinoa (cooked) — 175 g; Zucchini — 93 g; Extra virgin olive oil — 12 ml; Lemon juice — 5 ml

Snack 2. *Dark Chocolate and Almonds* — Dark chocolate (70% cocoa) — 12 g; Almonds — 12 g

Dinner. *Turkey Meatballs with Salad* — Turkey breast — 140 g; Whole wheat bread — 17 g; Egg — 29 g; Onion — 23 g; Extra virgin olive oil — 9 ml; Tomato — 93 g; Cucumber — 70 g

Day 5

Breakfast. *Dakos-Style Rusk with Tomato and Feta* — Barley rusk (paximadi) — 47 g; Extra virgin olive oil — 9

ml; Tomato — 93 g; Feta cheese — 23 g; Dried oregano — 1 g

Snack 1. *Dried Dates and Almonds* — Dried dates — 23 g; Almonds — 12 g

Lunch. *Chickpea and Vegetable Stew (Revithia)* — Chickpeas (cooked) — 233 g; Tomato — 117 g; Onion — 47 g; Carrot — 47 g; Extra virgin olive oil — 14 ml; Whole wheat bread — 35 g

Snack 2. *Greek Yogurt with Cinnamon and Honey* — Greek yogurt (2% fat) — 140 g; Cinnamon — 1 g; Honey — 9 g

Dinner. *Stuffed Zucchini with Rice* — Zucchini — 233 g; Brown rice (cooked) — 117 g; Onion — 35 g; Tomato — 70 g; Extra virgin olive oil — 12 ml

Day 6

Breakfast. *Boiled Egg, Toast, Avocado and Cucumber* — Egg — 117 g; Whole wheat bread — 47 g; Avocado — 47 g; Cucumber — 47 g **Snack 1.** *Cottage Cheese with Strawberries* — Cottage cheese — 93 g; Strawberries — 70 g

Lunch. *Whole Wheat Pasta with Sardines and Tomato Sauce* — Whole wheat pasta (cooked) — 233 g; Sardines, canned in olive oil — 93 g; Tomato — 140 g; Extra virgin olive oil — 9 ml; Garlic — 5 g

Snack 2. *Walnuts and Dried Figs* — Walnuts — 17 g; Dried dates — 23 g

Dinner. *Braised Mixed Vegetables (Briam)* — Eggplant — 140 g; Zucchini — 140 g; Potato — 93 g; Tomato — 117 g; Extra virgin olive oil — 14 ml

Day 7

Breakfast. *Greek Yogurt with Honey, Walnuts and Fresh Fig* — Greek yogurt (0% fat) — 175 g; Honey — 12 g; Walnuts — 12 g; Fresh fig — 70 g **Snack 1.** *Toast with Tahini and Honey* — Whole wheat bread — 23 g; Tahini — 17 g; Honey — 6 g

Lunch. *White Bean Soup (Fasolada)* — White beans (cooked) — 257 g; Carrot — 47 g; Celery — 35 g; Tomato — 93 g; Extra virgin olive oil — 14 ml; Whole wheat bread — 35 g

Snack 2. *Cucumber and Carrot Sticks with Yogurt Dip* — Greek yogurt (0% fat) — 70 g; Cucumber — 47 g; Carrot — 70 g; Garlic — 2 g; Extra virgin olive oil — 3 ml

Dinner. *Grilled Sea Bream with Vegetables* — Sea bream fillet — 152 g; Zucchini — 93 g; Bell pepper — 70 g; Extra virgin olive oil — 12 ml; Lemon juice — 5 ml

Day 8

Breakfast. *Cottage Cheese, Toast and Grapes* — Cottage cheese — 117 g; Whole wheat bread — 35 g; Grapes — 93 g; Almonds — 12 g **Snack 1.** *Apple with Walnuts* — Apple — 140 g; Walnuts — 17 g

Lunch. *Grilled Shrimp with Brown Rice and Vegetables* — Shrimp — 140 g; Brown rice (cooked) — 175 g; Zucchini — 70 g; Bell pepper — 70 g; Extra virgin olive oil — 12 ml

Snack 2. *Apple with Almonds* — Apple — 117 g; Almonds — 17 g

Dinner. *White Bean and Spinach Stew* — White beans (cooked) — 210 g; Spinach — 117 g; Tomato — 93 g; Extra virgin olive oil — 12 ml; Whole wheat bread — 35 g

Day 9

Breakfast. *Oat and Banana Pan-Cake with Honey* — Rolled oats — 47 g; Milk (1.5% fat) — 117 ml; Egg — 58 g; Banana — 70 g; Honey — 6 g **Snack 1.** *Greek Yogurt with Honey* — Greek yogurt (0% fat) — 175 g; Honey — 12 g

Lunch. *Stuffed Peppers with Rice (Gemista, light)* — Bell pepper — 233 g; Brown rice (cooked) — 140 g; Onion — 35 g; Tomato — 70 g; Extra virgin olive oil — 14 ml

Snack 2. *Pumpkin Seeds and Orange* — Pumpkin seeds — 17 g; Orange — 117 g

Dinner. *Egg and Vegetable Scramble with Feta* — Egg — 117 g; Spinach — 70 g; Tomato — 70 g; Feta cheese — 35 g; Extra virgin olive oil — 9 ml; Whole wheat bread — 35 g

Day 10

Breakfast. *Muesli with Greek Yogurt, Figs and Walnuts* — Rolled oats — 35 g; Greek yogurt (2% fat) — 140 g;

Dried dates — 23 g; Walnuts — 12 g; Honey — 6 g

Snack 1. *Hummus with Carrot and Cucumber Sticks* — Hummus — 70 g; Carrot — 70 g; Cucumber — 70 g

Lunch. *Turkey and Vegetable Pita Wrap* — Whole wheat pita bread — 70 g; Turkey breast — 117 g; Lettuce — 35 g; Tomato — 70 g; Cucumber — 47 g; Extra virgin olive oil — 9 ml

Snack 2. *Barley Rusk with Feta* — Barley rusk (paximadi) — 23 g; Feta cheese — 23 g

Dinner. *Grilled Chicken with Roasted Vegetables* — Chicken breast — 152 g; Zucchini — 93 g; Bell pepper — 70 g; Eggplant — 70 g; Extra virgin olive oil — 14 ml

Day 11

Breakfast. *Greek Yogurt with Honey, Walnuts and Strawberries* — Greek yogurt (2% fat) — 175 g; Honey — 17 g; Walnuts — 17 g; Strawberries — 93 g

Snack 1. *Almonds and Orange* — Almonds — 17 g; Orange — 117 g

Lunch. *Baked Sea Bream with Potatoes* — Sea bream fillet — 175 g; Potato — 210 g; Extra virgin olive oil — 14 ml; Lemon juice — 5 ml **Snack 2.** *Chia Pudding with Honey* — Chia seeds — 17 g; Milk (1.5% fat) — 117 ml; Honey — 6 g

Dinner. *Vegetable Omelette with Salad and Bread* — Egg — 117 g; Extra virgin olive oil — 9 ml; Zucchini — 70 g; Tomato — 70 g; Whole wheat bread — 35 g; Feta cheese — 23 g

Day 12

Breakfast. *Whole Wheat Toast with Feta and Tomato* — Whole wheat bread — 70 g; Feta cheese — 35 g; Tomato — 93 g; Extra virgin olive oil — 6 ml

Snack 1. *Feta and Grapes* — Feta cheese — 35 g; Grapes — 93 g

Lunch. *Chickpea Salad with Tuna* — Chickpeas (cooked) — 175 g; Tuna, canned in olive oil — 93 g; Tomato — 93 g; Cucumber — 70 g; Extra virgin olive oil — 9 ml

Snack 2. *Grapes and Walnuts* — Grapes — 117 g; Walnuts — 12 g

Dinner. *Chicken Souvlaki-Style with Salad and Pita* — Chicken breast — 152 g; Extra virgin olive oil — 9 ml; Lemon juice — 5 ml; Tomato — 93 g; Cucumber — 93 g; Whole wheat pita bread — 47 g

Day 13

Breakfast. *Vegetable Omelette with Toast* — Egg — 117 g; Extra virgin olive oil — 6 ml; Spinach — 47 g; Tomato — 47 g; Whole wheat bread — 35 g

Snack 1. *Barley Rusk with Honey* — Barley rusk (paximadi) — 29 g; Honey — 12 g

Lunch. *Green Bean and White Bean Stew with Feta* — Green beans — 175 g; White beans (cooked) — 117 g; Tomato — 117 g; Extra virgin olive oil — 14 ml; Feta cheese — 35 g; Whole wheat bread — 35 g

Snack 2. *Banana with Tahini* — Banana — 117 g; Tahini — 12 g

Dinner. *Baked Cod with Potatoes and Green Beans* — Cod fillet — 175 g; Extra virgin olive oil — 12 ml; Potato — 117 g; Green beans — 93 g

Day 14

Breakfast. *Overnight Oats with Banana and Chia* — Rolled oats — 47 g; Milk (1.5% fat) — 175 ml; Chia seeds — 9 g; Banana — 70 g; Honey — 6 g

Snack 1. *Fresh Watermelon Slices* — Watermelon — 175 g

Lunch. *Eggplant and Chickpea Stew (Briam-style)* — Eggplant — 175 g; Zucchini — 117 g; Chickpeas (cooked) — 117 g; Tomato — 117 g; Extra virgin olive oil — 14 ml; Whole wheat bread — 35 g

Snack 2. *Dark Chocolate and Almonds* — Dark chocolate (70% cocoa) — 12 g; Almonds — 12 g

Dinner. *Lentil and Vegetable Salad with Feta* — Lentils (cooked) — 210 g; Tomato — 93 g; Cucumber — 70 g; Onion — 23 g; Extra virgin olive oil — 12 ml; Feta cheese — 23 g

Day 15

Breakfast. *Dakos-Style Rusk with Tomato and Feta* — Barley rusk (paximadi) — 47 g; Extra virgin olive oil — 9

ml; Tomato — 93 g; Feta cheese — 23 g; Dried oregano — 1 g

Snack 1. *Dried Dates and Almonds* — Dried dates — 23 g; Almonds — 12 g

Lunch. *Greek Salad with Chickpeas and Bread* — Tomato — 175 g; Cucumber — 117 g; Bell pepper — 70 g; Kalamata olives — 23 g; Feta cheese — 47 g; Extra virgin olive oil — 17 ml; Chickpeas (cooked) — 117 g; Whole wheat bread — 35 g

Snack 2. *Greek Yogurt with Cinnamon and Honey* — Greek yogurt (2% fat) — 140 g; Cinnamon — 1 g; Honey — 9 g

Dinner. *Shrimp Saganaki (Tomato and Feta)* — Shrimp — 140 g; Tomato — 175 g; Feta cheese — 47 g; Extra virgin olive oil — 12 ml; Whole wheat bread — 35 g

Day 16

Breakfast. *Boiled Egg, Toast, Avocado and Cucumber* — Egg — 117 g; Whole wheat bread — 47 g; Avocado — 47 g; Cucumber — 47 g **Snack 1.** *Cottage Cheese with Strawberries* — Cottage cheese — 93 g; Strawberries — 70 g

Lunch. *Lentil Soup (Fakes)* — Lentils (cooked) — 292 g; Onion — 47 g; Carrot — 47 g; Tomato — 70 g; Extra virgin olive oil — 14 ml; Whole wheat bread — 47 g

Snack 2. *Walnuts and Dried Figs* — Walnuts — 17 g; Dried dates — 23 g

Dinner. *Vegetable and Chickpea Soup* — Chickpeas (cooked) — 175 g; Carrot — 47 g; Celery — 35 g; Tomato — 93 g; Extra virgin olive oil — 12 ml

Day 17

Breakfast. *Greek Yogurt with Honey, Walnuts and Fresh Fig* — Greek yogurt (0% fat) — 175 g; Honey — 12 g; Walnuts — 12 g; Fresh fig — 70 g **Snack 1.** *Toast with Tahini and Honey* — Whole wheat bread — 23 g; Tahini — 17 g; Honey — 6 g

Lunch. *Grilled Chicken with Bulgur Pilaf and Salad* — Chicken breast — 140 g; Bulgur (cooked) — 175 g; Extra virgin olive oil — 12 ml; Tomato — 93 g; Cucumber — 70 g; Lemon juice — 5 ml

Snack 2. *Cucumber and Carrot Sticks with Yogurt Dip* — Greek yogurt (0% fat) — 70 g; Cucumber — 47 g; Carrot — 70 g; Garlic — 2 g; Extra virgin olive oil — 3 ml

Dinner. *Grilled Salmon with Greek Salad* — Salmon fillet — 117 g; Tomato — 117 g; Cucumber — 93 g; Kalamata olives — 17 g; Feta cheese — 29 g; Extra virgin olive oil — 12 ml

Day 18

Breakfast. *Cottage Cheese, Toast and Grapes* — Cottage cheese — 117 g; Whole wheat bread — 35 g; Grapes — 93 g; Almonds — 12 g **Snack 1.** *Apple with Walnuts* — Apple — 140 g; Walnuts — 17 g

Lunch. *Baked Salmon with Quinoa and Zucchini* — Salmon fillet — 140 g; Quinoa (cooked) — 175 g; Zucchini — 93 g; Extra virgin olive oil — 12 ml; Lemon juice — 5 ml

Snack 2. *Apple with Almonds* — Apple — 117 g; Almonds — 17 g

Dinner. *Turkey Meatballs with Salad* — Turkey breast — 140 g; Whole wheat bread — 17 g; Egg — 29 g; Onion — 23 g; Extra virgin olive oil — 9 ml; Tomato — 93 g; Cucumber — 70 g

Day 19

Breakfast. *Oat and Banana Pan-Cake with Honey* — Rolled oats — 47 g; Milk (1.5% fat) — 117 ml; Egg — 58 g; Banana — 70 g; Honey — 6 g **Snack 1.** *Greek Yogurt with Honey* — Greek yogurt (0% fat) — 175 g; Honey — 12 g

Lunch. *Chickpea and Vegetable Stew (Revithia)* — Chickpeas (cooked) — 233 g; Tomato — 117 g; Onion — 47 g; Carrot — 47 g; Extra virgin olive oil — 14 ml; Whole wheat bread — 35 g

Snack 2. *Pumpkin Seeds and Orange* — Pumpkin seeds — 17 g; Orange — 117 g

Dinner. *Stuffed Zucchini with Rice* — Zucchini — 233 g; Brown rice (cooked) — 117 g; Onion — 35 g; Tomato — 70 g; Extra virgin olive oil — 12 ml

Day 20

Breakfast. *Muesli with Greek Yogurt, Figs and Walnuts* — Rolled oats — 35 g; Greek yogurt (2% fat) — 140 g; Dried dates — 23 g; Walnuts — 12 g; Honey — 6 g

Snack 1. *Hummus with Carrot and Cucumber Sticks* — Hummus — 70 g; Carrot — 70 g; Cucumber — 70 g

Lunch. *Whole Wheat Pasta with Sardines and Tomato Sauce* — Whole wheat pasta (cooked) — 233 g; Sardines, canned in olive oil — 93 g; Tomato — 140 g; Extra virgin olive oil — 9 ml; Garlic — 5 g

Snack 2. *Barley Rusk with Feta* — Barley rusk (paximadi) — 23 g; Feta cheese — 23 g

Dinner. *Braised Mixed Vegetables (Briam)* — Eggplant — 140 g; Zucchini — 140 g; Potato — 93 g; Tomato — 117 g; Extra virgin olive oil — 14 ml

Day 21

Breakfast. *Greek Yogurt with Honey, Walnuts and Strawberries* — Greek yogurt (2% fat) — 175 g; Honey — 17 g; Walnuts — 17 g; Strawberries — 93 g

Snack 1. *Almonds and Orange* — Almonds — 17 g; Orange — 117 g

Lunch. *White Bean Soup (Fasolada)* — White beans (cooked) — 257 g; Carrot — 47 g; Celery — 35 g; Tomato — 93 g; Extra virgin olive oil — 14 ml; Whole wheat bread — 35 g

Snack 2. *Chia Pudding with Honey* — Chia seeds — 17 g; Milk (1.5% fat) — 117 ml; Honey — 6 g

Dinner. *Grilled Sea Bream with Vegetables* — Sea bream fillet — 152 g; Zucchini — 93 g; Bell pepper — 70 g; Extra virgin olive oil — 12 ml; Lemon juice — 5 ml

Day 22

Breakfast. *Whole Wheat Toast with Feta and Tomato* — Whole wheat bread — 70 g; Feta cheese — 35 g; Tomato — 93 g; Extra virgin olive oil — 6 ml

Snack 1. *Feta and Grapes* — Feta cheese — 35 g; Grapes — 93 g

Lunch. *Grilled Shrimp with Brown Rice and Vegetables* — Shrimp — 140 g; Brown rice (cooked) — 175 g; Zucchini — 70 g; Bell pepper — 70 g; Extra virgin olive oil — 12 ml

Snack 2. *Grapes and Walnuts* — Grapes — 117 g; Walnuts — 12 g

Dinner. *White Bean and Spinach Stew* — White beans (cooked) — 210 g; Spinach — 117 g; Tomato — 93 g; Extra virgin olive oil — 12 ml; Whole wheat bread — 35 g

Day 23

Breakfast. *Vegetable Omelette with Toast* — Egg — 117 g; Extra virgin olive oil — 6 ml; Spinach — 47 g; Tomato — 47 g; Whole wheat bread — 35 g

Snack 1. *Barley Rusk with Honey* — Barley rusk (paximadi) — 29 g; Honey — 12 g

Lunch. *Stuffed Peppers with Rice (Gemista, light)* — Bell pepper — 233 g; Brown rice (cooked) — 140 g; Onion — 35 g; Tomato — 70 g; Extra virgin olive oil — 14 ml

Snack 2. *Banana with Tahini* — Banana — 117 g; Tahini — 12 g

Dinner. *Egg and Vegetable Scramble with Feta* — Egg — 117 g; Spinach — 70 g; Tomato — 70 g; Feta cheese — 35 g; Extra virgin olive oil — 9 ml; Whole wheat bread — 35 g

Day 24

Breakfast. *Overnight Oats with Banana and Chia* — Rolled oats — 47 g; Milk (1.5% fat) — 175 ml; Chia seeds — 9 g; Banana — 70 g; Honey — 6 g

Snack 1. *Fresh Watermelon Slices* — Watermelon — 175 g

Lunch. *Turkey and Vegetable Pita Wrap* — Whole wheat pita bread — 70 g; Turkey breast — 117 g; Lettuce — 35 g; Tomato — 70 g; Cucumber — 47 g; Extra virgin olive oil — 9 ml

Snack 2. *Dark Chocolate and Almonds* — Dark chocolate (70% cocoa) — 12 g; Almonds — 12 g

Dinner. *Grilled Chicken with Roasted Vegetables* — Chicken breast — 152 g; Zucchini — 93 g; Bell pepper — 70 g; Eggplant — 70 g; Extra virgin olive oil — 14 ml

Day 25

Breakfast. *Dakos-Style Rusk with Tomato and Feta* — Barley rusk (paximadi) — 47 g; Extra virgin olive oil — 9

ml; Tomato — 93 g; Feta cheese — 23 g; Dried oregano — 1 g

Snack 1. *Dried Dates and Almonds* — Dried dates — 23 g; Almonds — 12 g

Lunch. *Baked Sea Bream with Potatoes* — Sea bream fillet — 175 g; Potato — 210 g; Extra virgin olive oil — 14 ml; Lemon juice — 5 ml **Snack 2.** *Greek Yogurt with Cinnamon and Honey* — Greek yogurt (2% fat) — 140 g; Cinnamon — 1 g; Honey — 9 g

Dinner. *Vegetable Omelette with Salad and Bread* — Egg — 117 g; Extra virgin olive oil — 9 ml; Zucchini — 70 g; Tomato — 70 g; Whole wheat bread — 35 g; Feta cheese — 23 g

Day 26

Breakfast. *Boiled Egg, Toast, Avocado and Cucumber* — Egg — 117 g; Whole wheat bread — 47 g; Avocado — 47 g; Cucumber — 47 g **Snack 1.** *Cottage Cheese with Strawberries* — Cottage cheese — 93 g; Strawberries — 70 g

Lunch. *Chickpea Salad with Tuna* — Chickpeas (cooked) — 175 g; Tuna, canned in olive oil — 93 g; Tomato — 93 g; Cucumber — 70 g; Extra virgin olive oil — 9 ml

Snack 2. *Walnuts and Dried Figs* — Walnuts — 17 g; Dried dates — 23 g

Dinner. *Chicken Souvlaki-Style with Salad and Pita* — Chicken breast — 152 g; Extra virgin olive oil — 9 ml; Lemon juice — 5 ml; Tomato — 93 g; Cucumber — 93 g; Whole wheat pita bread — 47 g

Day 27

Breakfast. *Greek Yogurt with Honey, Walnuts and Fresh Fig* — Greek yogurt (0% fat) — 175 g; Honey — 12 g; Walnuts — 12 g; Fresh fig — 70 g **Snack 1.** *Toast with Tahini and Honey* — Whole wheat bread — 23 g; Tahini — 17 g; Honey — 6 g

Lunch. *Green Bean and White Bean Stew with Feta* — Green beans — 175 g; White beans (cooked) — 117 g; Tomato — 117 g; Extra virgin olive oil — 14 ml; Feta cheese — 35 g; Whole wheat bread — 35 g

Snack 2. *Cucumber and Carrot Sticks with Yogurt Dip* — Greek yogurt (0% fat) — 70 g; Cucumber — 47 g; Carrot — 70 g; Garlic — 2 g; Extra virgin olive oil — 3 ml

Dinner. *Baked Cod with Potatoes and Green Beans* — Cod fillet — 175 g; Extra virgin olive oil — 12 ml; Potato — 117 g; Green beans — 93 g

Day 28

Breakfast. *Cottage Cheese, Toast and Grapes* — Cottage cheese — 117 g; Whole wheat bread — 35 g; Grapes — 93 g; Almonds — 12 g **Snack 1.** *Apple with Walnuts* — Apple — 140 g; Walnuts — 17 g

Lunch. *Eggplant and Chickpea Stew (Briam-style)* — Eggplant — 175 g; Zucchini — 117 g; Chickpeas (cooked) — 117 g; Tomato — 117 g; Extra virgin olive oil — 14 ml; Whole wheat bread — 35 g

Snack 2. *Apple with Almonds* — Apple — 117 g; Almonds — 17 g

Dinner. *Lentil and Vegetable Salad with Feta* — Lentils (cooked) — 210 g; Tomato — 93 g; Cucumber — 70 g; Onion — 23 g; Extra virgin olive oil — 12 ml; Feta cheese — 23 g

3.2. Comparison with the MAI-DIET plan

The language-model plan reported a mean daily energy of 1595.0 kcal (-0.3 % of target) with a standard deviation of 98.4 kcal and a daily range of 1447 to 1791 kcal. The MAI-DIET plan for the same profile reported 1627.7 kcal (+1.7 %) with a standard deviation of 33.9 kcal. The language-model plan met 8 of the thirteen reference criteria and the MAI-DIET plan met 10. Day-to-day dispersion was 2.9 times greater in the language-model plan, so that individual days departed from the daily tolerance band even though the 28-day mean sat close to the target. Sodium was 1071.3 mg/day against 1878.0 mg/day for MAI-DIET, and calcium 714.7 mg/day against 783.5 mg/day. Protein was 92.9 g/day against 85.9 g/day, both above the reference range for this profile. The plan contained 58 unique dishes across 140 meal slots.

3.3. Compliance heat map

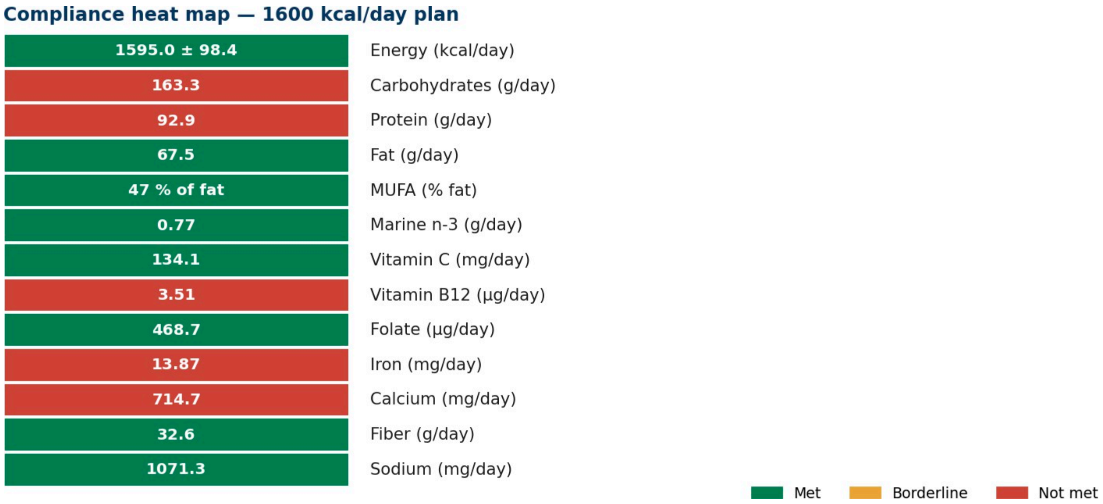

Figure S2. Compliance of the 1600 kcal/day language-model plan against the thirteen reference criteria applied in **Supplementary Table S7**. Values shown within each cell are the mean daily values reported by the model.

4. Plan 2 — 1800 kcal/day

**Sex**	Female	**Age**	35 y	**Height**	163 cm
**Body mass**	65 kg	**Activity**	1.55 (moderately active)	**Goal**	Mild weight loss

4.1. Twenty-eight-day meal plan

Day 1

Breakfast. *Greek Yogurt Bowl with Walnuts, Honey & Orange* — Greek yogurt (2% fat) — 200 g; Walnuts — 15 g; Honey — 8 g; Orange — 175 g **Snack 1.** *Kiwi with Almonds* — Almonds — 10 g; Kiwi — 150 g

Lunch. *Grilled Chicken Breast with Brown Rice & Greek Salad* — Chicken breast (cooked) — 120 g; Tomato — 100 g; Cucumber — 80 g; Red onion — 20 g; Feta cheese — 20 g; Extra virgin olive oil — 8 ml; Brown rice (cooked) — 155 g

Snack 2. *Apple with Walnuts* — Walnuts — 12 g; Apple — 140 g

Dinner. *Grilled Mackerel with Boiled Wild Greens (Horta), Potatoes & Feta* — Mackerel fillet (grilled) — 100 g; Boiled wild greens (horta) — 150 g; Extra virgin olive oil — 15 ml; Lemon juice — 5 ml; Feta cheese — 25 g; Potato (boiled) — 190 g

Day 2

Breakfast. *Whole Wheat Toast with Feta Cheese, Tomato, Boiled Egg & Olive Oil* — Feta cheese — 30 g; Tomato — 60 g; Extra virgin olive oil — 5 ml; Egg (whole) — 50 g; Whole wheat bread — 60 g

Snack 1. *Plain Greek Yogurt with a Touch of Honey* — Honey — 5 g; Greek yogurt (2% fat) — 185 g

Lunch. *Greek Lentil Soup (Fakes) with Whole Wheat Bread & Side Salad* — Lentils (cooked) — 250 g; Carrot — 30 g; Celery — 20 g; Extra virgin olive oil — 8 ml; Lettuce — 50 g; Tomato — 50 g; Whole wheat bread — 65 g

Snack 2. *Greek Yogurt with Pomegranate Seeds* — Pomegranate seeds — 40 g; Greek yogurt (2% fat) — 160 g

Dinner. *Greek White Bean Soup (Fasolada) with Bread* — White beans (cooked) — 250 g; Carrot — 40 g; Celery — 20 g; Tomato — 40 g; Extra virgin olive oil — 8 ml; Whole wheat bread — 65 g

Day 3

Breakfast. *Cinnamon Oatmeal with Milk, Almonds & Grapes* — Low-fat milk — 200 ml; Almonds — 10 g; Honey — 5 g; Rolled oats — 50 g; Grapes — 60 g

Snack 1. *Whole Wheat Crackers with Low-Fat Cottage Cheese* — Cottage cheese (low-fat) — 60 g; Whole wheat crackers — 25 g

Lunch. *Baked Salmon with Quinoa & Steamed Broccoli* — Salmon fillet (baked) — 120 g; Broccoli — 120 g; Extra virgin olive oil — 6 ml; Lemon juice — 5 ml; Quinoa (cooked) — 175 g

Snack 2. *Whole Grain Rusk with Honey* — Honey — 8 g; Barley rusk (paximadi) — 40 g

Dinner. *Oven-Baked Chicken with Roasted Mediterranean Vegetables* — Chicken breast (cooked) — 130 g; Zucchini — 100 g; Eggplant — 100 g; Bell pepper — 60 g; Extra virgin olive oil — 10 ml; Potato (boiled) — 260 g

Day 4

Breakfast. *Boiled Eggs with Whole Wheat Bread, Olives & Tomato* — Egg (whole) — 100 g; Kalamata olives — 20 g; Tomato — 60 g; Whole wheat bread — 70 g

Snack 1. *Homemade Hummus with Cucumber & Carrot Sticks* — Chickpeas (cooked) — 50 g; Tahini (sesame paste) — 6 g; Lemon juice — 5 ml; Cucumber — 50 g; Carrot — 50 g

Lunch. *Greek Chickpea Stew (Revithia) with Bread & Salad* — Chickpeas (cooked) — 220 g; Tomato — 60 g; Red onion — 30 g; Extra virgin olive oil — 8 ml; Lettuce — 50 g; Whole wheat bread — 35 g

Snack 2. *Carrot & Celery Sticks with Tahini Dip & Pita* — Carrot — 60 g; Celery — 40 g; Tahini (sesame paste) — 10 g; Whole wheat pita bread — 25 g

Dinner. *Shrimp & Vegetable Stir-Fry with Brown Rice* — Shrimp (cooked) — 130 g; Bell pepper — 60 g; Zucchini — 80 g; Carrot — 40 g; Extra virgin olive oil — 8 ml; Brown rice (cooked) — 285 g

Day 5

Breakfast. *Cretan Dakos: Barley Rusk with Tomato, Feta & Olive Oil* — Feta cheese — 30 g; Tomato — 80 g; Extra virgin olive oil — 6 ml; Garlic — 3 g; Barley rusk (paximadi) — 60 g

Snack 1. *Grapes with a Square of Dark Chocolate* — Dark chocolate (70% cocoa) — 8 g; Grapes — 150 g

Lunch. *Whole Wheat Pasta with Shrimp & Cherry Tomato Sauce* — Shrimp (cooked) — 120 g; Tomato — 150 g; Garlic — 5 g; Extra virgin olive oil — 8 ml; Whole wheat pasta (cooked) — 265 g

Snack 2. *Almonds with Dried Dates* — Almonds — 12 g; Dried dates — 30 g

Dinner. *Stuffed Peppers & Tomatoes (Gemista) with Turkey & Rice* — Bell pepper — 150 g; Tomato — 150 g; Turkey breast (cooked) — 60 g; Extra virgin olive oil — 10 ml; Brown rice (cooked) — 280 g

Day 6

Breakfast. *Greek Yogurt with Toasted Oats, Pistachios, Honey & Pear* — Greek yogurt (2% fat) — 150 g; Rolled oats — 20 g; Pistachios — 10 g; Honey — 5 g; Pear — 165 g

Snack 1. *Pistachios with Dried Apricots* — Pistachios — 15 g; Dried apricots — 25 g

Lunch. *Greek Salad with Grilled Halloumi & Whole Wheat Pita* — Tomato — 120 g; Cucumber — 100 g; Red onion — 30 g; Bell pepper — 40 g; Kalamata olives — 20 g; Halloumi cheese — 60 g; Extra virgin olive oil — 8 ml; Whole wheat pita bread — 80 g

Snack 2. *Low-Fat Cottage Cheese with Cucumber Slices* — Cottage cheese (low-fat) — 80 g; Cucumber — 300 g

Dinner. *Baked Cod with Lemon, Green Beans, Almonds & Potatoes* — Cod fillet (baked) — 150 g; Green beans — 120 g; Extra virgin olive oil — 15 ml; Lemon juice — 5 ml; Almonds — 10 g; Potato (boiled) — 245 g

Day 7

Breakfast. *Vegetable Omelette (Pepper & Spinach) with Whole Wheat Bread* — Egg (whole) — 100 g; Bell pepper — 40 g; Spinach — 30 g; Extra virgin olive oil — 5 ml; Whole wheat bread — 60 g

Snack 1. *Low-Fat Milk with Walnuts* — Walnuts — 10 g; Low-fat milk — 185 ml

Lunch. *Grilled Turkey Breast Souvlaki-Style with Bulgur Pilaf & Salad* — Turkey breast (cooked) — 120 g; Tomato — 60 g; Cucumber — 60 g; Extra virgin olive oil — 10 ml; Red onion — 20 g; Bulgur (cooked) — 295 g

Snack 2. *Banana with a Square of Dark Chocolate* — Dark chocolate (70% cocoa) — 10 g; Banana — 100 g

Dinner. *Mediterranean Lentil & Vegetable Stew with Feta Crumbles* — Lentils (cooked) — 220 g; Carrot — 30 g; Zucchini — 60 g; Feta cheese — 20 g; Extra virgin olive oil — 6 ml; Whole wheat bread — 90 g

Day 8

Breakfast. *Greek Yogurt Bowl with Walnuts, Honey & Apple* — Greek yogurt (2% fat) — 200 g; Walnuts — 15 g; Honey — 8 g; Apple — 155 g **Snack 1.** *Orange with Almonds* — Almonds — 10 g; Orange — 195 g

Lunch. *Grilled Turkey Breast with Brown Rice & Greek Salad* — Turkey breast (cooked) — 120 g; Tomato — 100 g; Cucumber — 80 g; Red onion — 20 g; Feta cheese — 20 g; Extra virgin olive oil — 8 ml; Brown rice (cooked) — 165 g **Snack 2.** *Pear with Walnuts* — Walnuts — 12 g; Pear — 125 g

Dinner. *Grilled Sardines with Boiled Wild Greens (Horta), Potatoes & Feta* — Sardines (grilled) — 100 g; Boiled wild greens (horta) — 150 g; Extra virgin olive oil — 15 ml; Lemon juice — 5 ml; Feta cheese — 25 g; Potato (boiled) — 185 g

Day 9

Breakfast. *Whole Wheat Toast with Feta Cheese, Tomato, Boiled Egg & Olive Oil* — Feta cheese — 30 g; Tomato — 60 g; Extra virgin olive oil — 5 ml; Egg (whole) — 50 g; Whole wheat bread — 60 g

Snack 1. *Plain Greek Yogurt with a Touch of Honey* — Honey — 5 g; Greek yogurt (2% fat) — 185 g

Lunch. *Greek Lentil Soup (Fakes) with Whole Wheat Bread & Side Salad* — Lentils (cooked) — 250 g; Carrot — 30 g; Celery — 20 g; Extra virgin olive oil — 8 ml; Lettuce — 50 g; Tomato — 50 g; Whole wheat bread — 65 g

Snack 2. *Greek Yogurt with Pomegranate Seeds* — Pomegranate seeds — 40 g; Greek yogurt (2% fat) — 160 g

Dinner. *Greek White Bean Soup (Fasolada) with Bread* — White beans (cooked) — 250 g; Carrot — 40 g; Celery — 20 g; Tomato — 40 g; Extra virgin olive oil — 8 ml; Whole wheat bread — 65 g

Day 10

Breakfast. *Cinnamon Oatmeal with Milk, Almonds & Strawberries* — Low-fat milk — 200 ml; Almonds — 10 g; Honey — 5 g; Rolled oats — 50 g; Strawberries — 60 g

Snack 1. *Whole Wheat Crackers with Low-Fat Cottage Cheese* — Cottage cheese (low-fat) — 60 g; Whole wheat crackers — 25 g

Lunch. *Grilled Mackerel with Quinoa & Steamed Broccoli* — Mackerel fillet (grilled) — 120 g; Broccoli — 120 g; Extra virgin olive oil — 6 ml; Lemon juice — 5 ml; Quinoa (cooked) — 175 g

Snack 2. *Whole Grain Rusk with Honey* — Honey — 8 g; Barley rusk (paximadi) — 40 g

Dinner. *Oven-Baked Chicken with Roasted Mediterranean Vegetables* — Chicken breast (cooked) — 130 g; Zucchini — 100 g; Eggplant — 100 g; Bell pepper — 60 g; Extra virgin olive oil — 10 ml; Potato (boiled) — 260 g

Day 11

Breakfast. *Boiled Eggs with Whole Wheat Bread, Olives & Tomato* — Egg (whole) — 100 g; Kalamata olives — 20 g; Tomato — 60 g; Whole wheat bread — 70 g

Snack 1. *Homemade Hummus with Cucumber & Carrot Sticks* — Chickpeas (cooked) — 50 g; Tahini (sesame paste) — 6 g; Lemon juice — 5 ml; Cucumber — 50 g; Carrot — 50 g

Lunch. *Greek Chickpea Stew (Revithia) with Bread & Salad* — Chickpeas (cooked) — 220 g; Tomato — 60 g; Red onion — 30 g; Extra virgin olive oil — 8 ml; Lettuce — 50 g; Whole wheat bread — 35 g

Snack 2. *Carrot & Celery Sticks with Tahini Dip & Pita* — Carrot — 60 g; Celery — 40 g; Tahini (sesame paste) — 10 g; Whole wheat pita bread — 25 g

Dinner. *Shrimp & Vegetable Stir-Fry with Brown Rice* — Shrimp (cooked) — 130 g; Bell pepper — 60 g; Zucchini — 80 g; Carrot — 40 g; Extra virgin olive oil — 8 ml; Brown rice (cooked) — 285 g

Day 12

Breakfast. *Cretan Dakos: Barley Rusk with Tomato, Feta & Olive Oil* — Feta cheese — 30 g; Tomato — 80 g; Extra virgin olive oil — 6 ml; Garlic — 3 g; Barley rusk (paximadi) — 60 g

Snack 1. *Strawberries with a Square of Dark Chocolate* — Dark chocolate (70% cocoa) — 8 g; Strawberries — 300 g

Lunch. *Whole Wheat Pasta with Shrimp & Cherry Tomato Sauce* — Shrimp (cooked) — 120 g; Tomato — 150 g; Garlic — 5 g; Extra virgin olive oil — 8 ml; Whole wheat pasta (cooked) — 265 g

Snack 2. *Almonds with Dried Dates* — Almonds — 12 g; Dried dates — 30 g

Dinner. *Stuffed Peppers & Tomatoes (Gemista) with Turkey & Rice* — Bell pepper — 150 g; Tomato — 150 g; Turkey breast (cooked) — 60 g; Extra virgin olive oil — 10 ml; Brown rice (cooked) — 280 g

Day 13

Breakfast. *Greek Yogurt with Toasted Oats, Pistachios, Honey & Kiwi* — Greek yogurt (2% fat) — 150 g; Rolled oats — 20 g; Pistachios — 10 g; Honey — 5 g; Kiwi — 155 g

Snack 1. *Pistachios with Dried Apricots* — Pistachios — 15 g; Dried apricots — 25 g

Lunch. *Greek Salad with Grilled Halloumi & Whole Wheat Pita* — Tomato — 120 g; Cucumber — 100 g; Red onion — 30 g; Bell pepper — 40 g; Kalamata olives — 20 g; Halloumi cheese — 60 g; Extra virgin olive oil — 8 ml; Whole wheat pita bread — 80 g

Snack 2. *Low-Fat Cottage Cheese with Cucumber Slices* — Cottage cheese (low-fat) — 80 g; Cucumber — 300 g

Dinner. *Baked Cod with Lemon, Green Beans, Almonds & Potatoes* — Cod fillet (baked) — 150 g; Green beans — 120 g; Extra virgin olive oil — 15 ml; Lemon juice — 5 ml; Almonds — 10 g; Potato (boiled) — 245 g

Day 14

Breakfast. *Vegetable Omelette (Pepper & Spinach) with Whole Wheat Bread* — Egg (whole) — 100 g; Bell pepper — 40 g; Spinach — 30 g; Extra virgin olive oil — 5 ml; Whole wheat bread — 60 g

Snack 1. *Low-Fat Milk with Walnuts* — Walnuts — 10 g; Low-fat milk — 185 ml

Lunch. *Grilled Chicken Breast Souvlaki-Style with Bulgur Pilaf & Salad* — Chicken breast (cooked) — 120 g; Tomato — 60 g; Cucumber — 60 g; Extra virgin olive oil — 10 ml; Red onion — 20 g; Bulgur (cooked) — 285 g

Snack 2. *Banana with a Square of Dark Chocolate* — Dark chocolate (70% cocoa) — 10 g; Banana — 100 g

Dinner. *Mediterranean Lentil & Vegetable Stew with Feta Crumbles* — Lentils (cooked) — 220 g; Carrot — 30 g; Zucchini — 60 g; Feta cheese — 20 g; Extra virgin olive oil — 6 ml; Whole wheat bread — 90 g

Day 15

Breakfast. *Greek Yogurt Bowl with Walnuts, Honey & Pear* — Greek yogurt (2% fat) — 200 g; Walnuts — 15 g; Honey — 8 g; Pear — 145 g **Snack 1.** *Apple with Almonds* — Almonds — 10 g; Apple — 175 g

Lunch. *Grilled Chicken Breast with Brown Rice & Greek Salad* — Chicken breast (cooked) — 120 g; Tomato — 100 g; Cucumber — 80 g; Red onion — 20 g; Feta cheese — 20 g; Extra virgin olive oil — 8 ml; Brown rice (cooked) — 155 g

Snack 2. *Kiwi with Walnuts* — Walnuts — 12 g; Kiwi — 115 g

Dinner. *Grilled Sardines with Boiled Wild Greens (Horta), Potatoes & Feta* — Sardines (grilled) — 100 g; Boiled wild greens (horta) — 150 g; Extra virgin olive oil — 15 ml; Lemon juice — 5 ml; Feta cheese — 25 g; Potato (boiled) — 185 g

Day 16

Breakfast. *Whole Wheat Toast with Grilled Halloumi, Tomato, Boiled Egg & Olive Oil* — Halloumi cheese — 30 g; Tomato — 60 g; Extra virgin olive oil — 5 ml; Egg (whole) — 50 g; Whole wheat bread — 50 g

Snack 1. *Plain Greek Yogurt with a Touch of Honey* — Honey — 5 g; Greek yogurt (2% fat) — 185 g

Lunch. *Greek Lentil Soup (Fakes) with Whole Wheat Bread & Side Salad* — Lentils (cooked) — 250 g; Carrot — 30 g; Celery — 20 g; Extra virgin olive oil — 8 ml; Lettuce — 50 g; Tomato — 50 g; Whole wheat bread — 65 g

Snack 2. *Greek Yogurt with Pomegranate Seeds* — Pomegranate seeds — 40 g; Greek yogurt (2% fat) — 160 g

Dinner. *Greek White Bean Soup (Fasolada) with Bread* — White beans (cooked) — 250 g; Carrot — 40 g; Celery — 20 g; Tomato — 40 g; Extra virgin olive oil — 8 ml; Whole wheat bread — 65 g

Day 17

Breakfast. *Cinnamon Oatmeal with Milk, Almonds & Watermelon* — Low-fat milk — 200 ml; Almonds — 10 g; Honey — 5 g; Rolled oats — 50 g; Watermelon — 60 g

Snack 1. *Whole Wheat Crackers with Low-Fat Cottage Cheese* — Cottage cheese (low-fat) — 60 g; Whole wheat crackers — 25 g

Lunch. *Grilled Sardines with Quinoa & Steamed Broccoli* — Sardines (grilled) — 120 g; Broccoli — 120 g; Extra virgin olive oil — 6 ml; Lemon juice — 5 ml; Quinoa (cooked) — 170 g

Snack 2. *Whole Grain Rusk with Honey* — Honey — 8 g; Barley rusk (paximadi) — 40 g

Dinner. *Oven-Baked Chicken with Roasted Mediterranean Vegetables* — Chicken breast (cooked) — 130 g; Zucchini — 100 g; Eggplant — 100 g; Bell pepper — 60 g; Extra virgin olive oil — 10 ml; Potato (boiled) — 260 g

Day 18

Breakfast. *Boiled Eggs with Whole Wheat Bread, Olives & Tomato* — Egg (whole) — 100 g; Kalamata olives — 20 g; Tomato — 60 g; Whole wheat bread — 70 g

Snack 1. *Homemade Hummus with Cucumber & Carrot Sticks* — Chickpeas (cooked) — 50 g; Tahini (sesame paste) — 6 g; Lemon juice — 5 ml; Cucumber — 50 g; Carrot — 50 g

Lunch. *Greek Chickpea Stew (Revithia) with Bread & Salad* — Chickpeas (cooked) — 220 g; Tomato — 60 g; Red onion — 30 g; Extra virgin olive oil — 8 ml; Lettuce — 50 g; Whole wheat bread — 35 g

Snack 2. *Carrot & Celery Sticks with Tahini Dip & Pita* — Carrot — 60 g; Celery — 40 g; Tahini (sesame paste) — 10 g; Whole wheat pita bread — 25 g

Dinner. *Shrimp & Vegetable Stir-Fry with Brown Rice* — Shrimp (cooked) — 130 g; Bell pepper — 60 g; Zucchini — 80 g; Carrot — 40 g; Extra virgin olive oil — 8 ml; Brown rice (cooked) — 285 g

Day 19

Breakfast. *Cretan Dakos: Barley Rusk with Tomato, Feta & Olive Oil* — Feta cheese — 30 g; Tomato — 80 g; Extra virgin olive oil — 6 ml; Garlic — 3 g; Barley rusk (paximadi) — 60 g

Snack 1. *Watermelon with a Square of Dark Chocolate* — Dark chocolate (70% cocoa) — 8 g; Watermelon — 300 g

Lunch. *Whole Wheat Pasta with Shrimp & Cherry Tomato Sauce* — Shrimp (cooked) — 120 g; Tomato — 150 g; Garlic — 5 g; Extra virgin olive oil — 8 ml; Whole wheat pasta (cooked) — 265 g

Snack 2. *Almonds with Dried Dates* — Almonds — 12 g; Dried dates — 30 g

Dinner. *Stuffed Peppers & Tomatoes (Gemista) with Turkey & Rice* — Bell pepper — 150 g; Tomato — 150 g; Turkey breast (cooked) — 60 g; Extra virgin olive oil — 10 ml; Brown rice (cooked) — 280 g

Day 20

Breakfast. *Greek Yogurt with Toasted Oats, Pistachios, Honey & Orange* — Greek yogurt (2% fat) — 150 g; Rolled oats — 20 g; Pistachios — 10 g; Honey — 5 g; Orange — 200 g

Snack 1. *Pistachios with Dried Apricots* — Pistachios — 15 g; Dried apricots — 25 g

Lunch. *Greek Salad with Grilled Halloumi & Whole Wheat Pita* — Tomato — 120 g; Cucumber — 100 g; Red onion — 30 g; Bell pepper — 40 g; Kalamata olives — 20 g; Halloumi cheese — 60 g; Extra virgin olive oil — 8 ml; Whole wheat pita bread — 80 g

Snack 2. *Low-Fat Cottage Cheese with Cucumber Slices* — Cottage cheese (low-fat) — 80 g; Cucumber — 300 g

Dinner. *Baked Cod with Lemon, Green Beans, Almonds & Potatoes* — Cod fillet (baked) — 150 g; Green beans — 120 g; Extra virgin olive oil — 15 ml; Lemon juice — 5 ml; Almonds — 10 g; Potato (boiled) — 245 g

Day 21

Breakfast. *Vegetable Omelette (Pepper & Spinach) with Whole Wheat Bread* — Egg (whole) — 100 g; Bell pepper — 40 g; Spinach — 30 g; Extra virgin olive oil — 5 ml; Whole wheat bread — 60 g

Snack 1. *Low-Fat Milk with Walnuts* — Walnuts — 10 g; Low-fat milk — 185 ml

Lunch. *Grilled Turkey Breast Souvlaki-Style with Bulgur Pilaf & Salad* — Turkey breast (cooked) — 120 g; Tomato — 60 g; Cucumber — 60 g; Extra virgin olive oil — 10 ml; Red onion — 20 g; Bulgur (cooked) — 295 g

Snack 2. *Banana with a Square of Dark Chocolate* — Dark chocolate (70% cocoa) — 10 g; Banana — 100 g

Dinner. *Mediterranean Lentil & Vegetable Stew with Feta Crumbles* — Lentils (cooked) — 220 g; Carrot — 30 g; Zucchini — 60 g; Feta cheese — 20 g; Extra virgin olive oil — 6 ml; Whole wheat bread — 90 g

Day 22

Breakfast. *Greek Yogurt Bowl with Walnuts, Honey & Kiwi* — Greek yogurt (2% fat) — 200 g; Walnuts — 15 g; Honey — 8 g; Kiwi — 135 g

Snack 1. *Pear with Almonds* — Almonds — 10 g; Pear — 160 g

Lunch. *Grilled Turkey Breast with Brown Rice & Greek Salad* — Turkey breast (cooked) — 120 g; Tomato — 100 g; Cucumber — 80 g; Red onion — 20 g; Feta cheese — 20 g; Extra virgin olive oil — 8 ml; Brown rice (cooked) — 165 g

Snack 2. *Orange with Walnuts* — Walnuts — 12 g; Orange — 150 g

Dinner. *Grilled Sardines with Boiled Wild Greens (Horta), Potatoes & Feta* — Sardines (grilled) — 100 g; Boiled wild greens (horta) — 150 g; Extra virgin olive oil — 15 ml; Lemon juice — 5 ml; Feta cheese — 25 g; Potato (boiled) — 185 g

Day 23

Breakfast. *Whole Wheat Toast with Feta Cheese, Tomato, Boiled Egg & Olive Oil* — Feta cheese — 30 g; Tomato — 60 g; Extra virgin olive oil — 5 ml; Egg (whole) — 50 g; Whole wheat bread — 60 g

Snack 1. *Plain Greek Yogurt with a Touch of Honey* — Honey — 5 g; Greek yogurt (2% fat) — 185 g

Lunch. *Greek Lentil Soup (Fakes) with Whole Wheat Bread & Side Salad* — Lentils (cooked) — 250 g; Carrot — 30 g; Celery — 20 g; Extra virgin olive oil — 8 ml; Lettuce — 50 g; Tomato — 50 g; Whole wheat bread — 65 g

Snack 2. *Greek Yogurt with Pomegranate Seeds* — Pomegranate seeds — 40 g; Greek yogurt (2% fat) — 160 g

Dinner. *Greek White Bean Soup (Fasolada) with Bread* — White beans (cooked) — 250 g; Carrot — 40 g; Celery — 20 g; Tomato — 40 g; Extra virgin olive oil — 8 ml; Whole wheat bread — 65 g

Day 24

Breakfast. *Cinnamon Oatmeal with Milk, Almonds & Melon* — Low-fat milk — 200 ml; Almonds — 10 g; Honey — 5 g; Rolled oats — 50 g; Melon — 60 g

Snack 1. *Whole Wheat Crackers with Low-Fat Cottage Cheese* — Cottage cheese (low-fat) — 60 g; Whole wheat crackers — 25 g

Lunch. *Baked Cod with Quinoa & Steamed Broccoli* — Cod fillet (baked) — 120 g; Broccoli — 120 g; Extra virgin olive oil — 6 ml; Lemon juice — 5 ml; Quinoa (cooked) — 220 g

Snack 2. *Whole Grain Rusk with Honey* — Honey — 8 g; Barley rusk (paximadi) — 40 g

Dinner. *Oven-Baked Chicken with Roasted Mediterranean Vegetables* — Chicken breast (cooked) — 130 g; Zucchini — 100 g; Eggplant — 100 g; Bell pepper — 60 g; Extra virgin olive oil — 10 ml; Potato (boiled) — 260 g

Day 25

Breakfast. *Boiled Eggs with Whole Wheat Bread, Olives & Tomato* — Egg (whole) — 100 g; Kalamata olives — 20 g; Tomato — 60 g; Whole wheat bread — 70 g

Snack 1. *Homemade Hummus with Cucumber & Carrot Sticks* — Chickpeas (cooked) — 50 g; Tahini (sesame paste) — 6 g; Lemon juice — 5 ml; Cucumber — 50 g; Carrot — 50 g

Lunch. *Greek Chickpea Stew (Revithia) with Bread & Salad* — Chickpeas (cooked) — 220 g; Tomato — 60 g; Red onion — 30 g; Extra virgin olive oil — 8 ml; Lettuce — 50 g; Whole wheat bread — 35 g

Snack 2. *Carrot & Celery Sticks with Tahini Dip & Pita* — Carrot — 60 g; Celery — 40 g; Tahini (sesame paste) — 10 g; Whole wheat pita bread — 25 g

Dinner. *Shrimp & Vegetable Stir-Fry with Brown Rice* — Shrimp (cooked) — 130 g; Bell pepper — 60 g; Zucchini — 80 g; Carrot — 40 g; Extra virgin olive oil — 8 ml; Brown rice (cooked) — 285 g

Day 26

Breakfast. *Cretan Dakos: Barley Rusk with Tomato, Feta & Olive Oil* — Feta cheese — 30 g; Tomato — 80 g; Extra virgin olive oil — 6 ml; Garlic — 3 g; Barley rusk (paximadi) — 60 g

Snack 1. *Melon with a Square of Dark Chocolate* — Dark chocolate (70% cocoa) — 8 g; Melon — 300 g

Lunch. *Whole Wheat Pasta with Shrimp & Cherry Tomato Sauce* — Shrimp (cooked) — 120 g; Tomato — 150 g; Garlic — 5 g; Extra virgin olive oil — 8 ml; Whole wheat pasta (cooked) — 265 g

Snack 2. *Almonds with Dried Dates* — Almonds — 12 g; Dried dates — 30 g

Dinner. *Stuffed Peppers & Tomatoes (Gemista) with Turkey & Rice* — Bell pepper — 150 g; Tomato — 150 g; Turkey breast (cooked) — 60 g; Extra virgin olive oil — 10 ml; Brown rice (cooked) — 280 g

Day 27

Breakfast. *Greek Yogurt with Toasted Oats, Pistachios, Honey & Apple* — Greek yogurt (2% fat) — 150 g; Rolled oats — 20 g; Pistachios — 10 g; Honey — 5 g; Apple — 180 g

Snack 1. *Pistachios with Dried Apricots* — Pistachios — 15 g; Dried apricots — 25 g

Lunch. *Greek Salad with Grilled Halloumi & Whole Wheat Pita* — Tomato — 120 g; Cucumber — 100 g; Red onion — 30 g; Bell pepper — 40 g; Kalamata olives — 20 g; Halloumi cheese — 60 g; Extra virgin olive oil —

8 ml; Whole wheat pita bread — 80 g

Snack 2. *Low-Fat Cottage Cheese with Cucumber Slices* — Cottage cheese (low-fat) — 80 g; Cucumber — 300 g

Dinner. *Baked Cod with Lemon, Green Beans, Almonds & Potatoes* — Cod fillet (baked) — 150 g; Green beans — 120 g; Extra virgin olive oil — 15 ml; Lemon juice — 5 ml; Almonds — 10 g; Potato (boiled) — 245 g

Day 28

Breakfast. *Vegetable Omelette (Pepper & Spinach) with Whole Wheat Bread* — Egg (whole) — 100 g; Bell pepper — 40 g; Spinach — 30 g; Extra virgin olive oil — 5 ml; Whole wheat bread — 60 g

Snack 1. *Low-Fat Milk with Walnuts* — Walnuts — 10 g; Low-fat milk — 185 ml

Lunch. *Grilled Chicken Breast Souvlaki-Style with Bulgur Pilaf & Salad* — Chicken breast (cooked) — 120 g; Tomato — 60 g; Cucumber — 60 g; Extra virgin olive oil — 10 ml; Red onion — 20 g; Bulgur (cooked) — 285 g

Snack 2. *Banana with a Square of Dark Chocolate* — Dark chocolate (70% cocoa) — 10 g; Banana — 100 g

Dinner. *Mediterranean Lentil & Vegetable Stew with Feta Crumbles* — Lentils (cooked) — 220 g; Carrot — 30 g; Zucchini — 60 g; Feta cheese — 20 g; Extra virgin olive oil — 6 ml; Whole wheat bread — 90 g

4.2. Comparison with the MAI-DIET plan

The language-model plan reported a mean daily energy of 1798.4 kcal (-0.1 % of target) with a standard deviation of 23.0 kcal and a daily range of 1755 to 1851 kcal. The MAI-DIET plan for the same profile reported 1822.4 kcal (+1.2 %) with a standard deviation of 37.9 kcal. The language-model plan met 9 of the thirteen reference criteria and the MAI-DIET plan met 9. Day-to-day dispersion was lower than in the MAI-DIET plan. Sodium was 1110.8 mg/day against 2318.2 mg/day for MAI-DIET, and calcium 769.6 mg/day against 946.4 mg/day. Protein was 103.6 g/day against 96.8 g/day, both above the reference range for this profile. The plan contained 60 unique dishes across 140 meal slots.

4.3. Compliance heat map

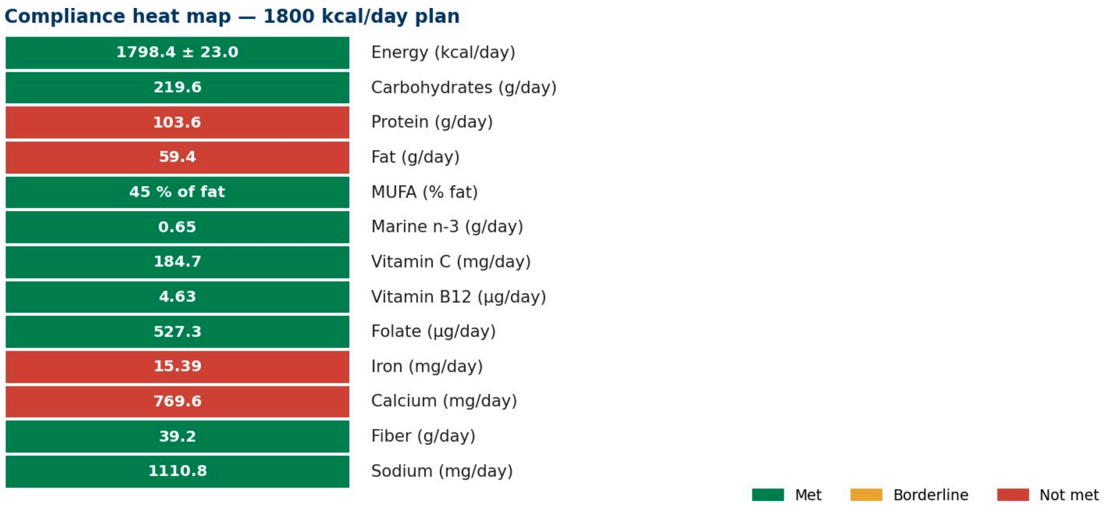

Figure S3. Compliance of the 1800 kcal/day language-model plan against the thirteen reference criteria applied in **Supplementary Table S7**. Values shown within each cell are the mean daily values reported by the model.

5. Plan 3 — 2000 kcal/day

**Sex**	Male	**Age**	35 y	**Height**	170 cm
**Body mass**	72 kg	**Activity**	1.55 (moderately active)	**Goal**	Mild weight loss

5.1. Twenty-eight-day meal plan

Day 1

Breakfast. *Greek Yogurt with Honey and Walnuts* — Greek yogurt (2%) — 255 g; Honey — 20 g; Walnuts — 20 g; Apple — 125 g **Snack 1.** *Grapes with Feta Cubes* — Grapes — 125 g; Feta cheese — 25 g

Lunch. *Wholewheat Pasta with Tuna and Tomato Sauce* — Wholewheat pasta (cooked) — 280 g; Tuna (canned in water) — 125 g; Tomato — 150 g; Onion — 40 g; Olive oil — 15 g

Snack 2. *Melon* — Melon — 255 g

Dinner. *Chicken and Vegetable Soup with Bread* — Chicken breast — 150 g; Carrot — 65 g; Celery — 40 g; Onion — 40 g; Wholewheat bread — 50 g; Olive oil — 10 g

Day 2

Breakfast. *Veggie Omelette with Wholewheat Toast* — Eggs — 150 g; Tomato — 75 g; Bell pepper — 40 g; Olive oil — 5 g; Wholewheat bread — 50 g

Snack 1. *Banana with Almonds* — Banana — 150 g; Almonds — 10 g

Lunch. *Beef and Vegetable Stew with Bulgur* — Lean beef — 165 g; Bulgur (cooked) — 230 g; Carrot — 65 g; Onion — 50 g; Tomato — 75 g; Olive oil — 15 g

Snack 2. *Walnuts with Figs* — Walnuts — 15 g; Fresh figs — 75 g

Dinner. *Black Bean Stew with Brown Rice* — Black beans (cooked) — 280 g; Brown rice (cooked) — 165 g; Tomato — 90 g; Onion — 40 g; Olive oil — 15 g

Day 3

Breakfast. *Oats with Milk, Banana and Almonds* — Oats — 65 g; Low-fat milk — 255 g; Banana — 125 g; Almonds — 15 g **Snack 1.** *Carrot Sticks with Hummus* — Carrot — 125 g; Hummus — 40 g

Lunch. *Shrimp with Brown Rice and Vegetables* — Shrimp — 190 g; Brown rice (cooked) — 205 g; Zucchini — 100 g; Bell pepper — 75 g; Olive oil — 15 g; Lemon juice — 10 g

Snack 2. *Strawberries with Greek Yogurt* — Strawberries — 125 g; Greek yogurt (2%) — 100 g

Dinner. *Grilled Shrimp with Couscous and Salad* — Shrimp — 180 g; Couscous (cooked) — 180 g; Lettuce — 50 g; Tomato — 75 g; Cucumber — 75 g; Olive oil — 15 g; Lemon juice — 10 g

Day 4

Breakfast. *Feta and Tomato Wholewheat Toast* — Wholewheat bread — 75 g; Feta cheese — 50 g; Tomato — 100 g; Olive oil — 5 g; Olives — 20 g **Snack 1.** *Melon Slices* — Melon — 255 g

Lunch. *White Bean Soup with Olive Oil and Bread* — White beans (cooked) — 320 g; Tomato — 100 g; Carrot — 50 g; Celery — 40 g; Olive oil — 15 g; Wholewheat bread — 65 g

Snack 2. *Pomegranate with Walnuts* — Pomegranate seeds — 125 g; Walnuts — 10 g

Dinner. *Grilled Halloumi and Vegetable Platter with Pita* — Halloumi cheese — 100 g; Zucchini — 125 g; Bell pepper — 100 g; Eggplant — 100 g; Olive oil — 15 g; Wholewheat pita — 50 g

Day 5

Breakfast. *Greek Yogurt Parfait with Strawberries* — Greek yogurt (2%) — 230 g; Strawberries — 125 g; Honey — 15 g; Sesame seeds — 15 g **Snack 1.** *Fresh Figs with Walnuts* — Fresh figs — 100 g; Walnuts — 10 g

Lunch. *Turkey Breast with Couscous and Roasted Vegetables* — Turkey breast — 180 g; Couscous (cooked) — 205 g; Zucchini — 90 g; Eggplant — 90 g; Olive oil — 15 g

Snack 2. *Mini Pita with Hummus* — Wholewheat pita — 30 g; Hummus — 30 g

Dinner. *Grilled Salmon with Roasted Vegetables and Potato* — Salmon fillet — 150 g; Potato — 190 g; Zucchini — 100 g; Bell pepper — 75 g; Olive oil — 15 g; Lemon juice — 10 g

Day 6

Breakfast. *Egg and Spinach Scramble with Bread* — Eggs — 150 g; Spinach — 75 g; Olive oil — 5 g; Wholewheat bread — 50 g **Snack 1.** *Cucumber with Yogurt Dip* — Cucumber — 125 g; Greek yogurt (2%) — 100 g

Lunch. *Baked Pork Loin with Potatoes and Green Beans* — Pork loin — 165 g; Potato — 255 g; Green beans — 125 g; Olive oil — 15 g **Snack 2.** *Kiwi with Almonds* — Kiwi — 150 g; Almonds — 15 g

Dinner. *Vegetable and Chickpea Bake* — Chickpeas (cooked) — 230 g; Eggplant — 190 g; Tomato — 125 g; Onion — 50 g; Olive oil — 15 g; Feta cheese — 40 g

Day 7

Breakfast. *Wholewheat Toast with Hummus and Cucumber* — Wholewheat bread — 75 g; Hummus — 50 g; Cucumber — 75 g; Olives — 15 g **Snack 1.** *Watermelon with Feta* — Watermelon — 190 g; Feta cheese — 25 g

Lunch. *Stuffed Peppers with Rice and Beef* — Lean beef (minced) — 150 g; Brown rice (cooked) — 190 g; Bell pepper — 190 g; Tomato — 75 g; Olive oil — 10 g

Snack 2. *Greek Yogurt with Walnuts* — Greek yogurt (2%) — 190 g; Walnuts — 15 g

Dinner. *Chicken Souvlaki with Salad and Pita* — Chicken breast — 190 g; Wholewheat pita — 65 g; Tomato — 100 g; Cucumber — 100 g; Onion — 25 g; Olive oil — 15 g; Greek yogurt (2%) — 65 g

Day 8

Breakfast. *Cinnamon Oats Cooked in Milk* — Oats — 55 g; Low-fat milk — 280 g; Honey — 15 g; Cinnamon — 2 g **Snack 1.** *Apple with Walnuts* — Apple — 165 g; Walnuts — 10 g

Lunch. *Sardines with Wholewheat Bread and Salad* — Sardines (canned) — 125 g; Wholewheat bread — 75 g; Tomato — 100 g; Cucumber — 75 g; Olive oil — 15 g; Lemon juice — 10 g

Snack 2. *Hummus with Cucumber and Carrot* — Hummus — 50 g; Cucumber — 75 g; Carrot — 75 g

Dinner. *Garlic Shrimp with Brown Rice and Spinach* — Shrimp — 180 g; Brown rice (cooked) — 180 g; Spinach — 100 g; Olive oil — 15 g; Lemon juice — 10 g

Day 9

Breakfast. *Greek Yogurt with Figs and Walnuts* — Greek yogurt (2%) — 255 g; Fresh figs — 100 g; Walnuts — 20 g **Snack 1.** *Strawberries with Almonds* — Strawberries — 190 g; Almonds — 15 g

Lunch. *Black Bean and Quinoa Bowl* — Black beans (cooked) — 280 g; Quinoa (cooked) — 190 g; Bell pepper — 75 g; Tomato — 75 g; Olive oil — 15 g; Lemon juice — 10 g

Snack 2. *Orange* — Orange — 255 g

Dinner. *Lentil and Vegetable Stew* — Lentils (cooked) — 280 g; Carrot — 65 g; Onion — 50 g; Tomato — 100 g; Olive oil — 15 g; Wholewheat bread — 40 g

Day 10

Breakfast. *Grilled Halloumi Toast with Tomato* — Wholewheat bread — 65 g; Halloumi cheese — 50 g; Tomato — 90 g; Olive oil — 5 g **Snack 1.** *Mini Pita with Hummus* — Wholewheat pita — 25 g; Hummus — 30 g

Lunch. *Grilled Chicken Breast with Brown Rice and Salad* — Chicken breast — 190 g; Brown rice (cooked) — 230 g; Tomato — 100 g; Cucumber — 100 g; Olive oil — 15 g; Lemon juice — 10 g; Lettuce — 50 g **Snack 2.** *Almonds with Apple* — Almonds — 25 g; Apple — 100 g

Dinner. *Turkey Meatballs with Tomato Sauce and Bulgur* — Turkey breast (minced) — 180 g; Bulgur (cooked) — 190 g; Tomato — 150 g; Onion — 40 g; Olive oil — 15 g

Day 11

Breakfast. *Banana Almond Breakfast Bowl* — Low-fat milk — 190 g; Banana — 150 g; Almonds — 20 g; Oats — 25 g **Snack 1.** *Kiwi with Walnuts* — Kiwi — 190 g; Walnuts — 10 g

Lunch. *Chickpea and Vegetable Stew with Wholewheat Bread* — Chickpeas (cooked) — 280 g; Tomato — 125 g; Onion — 65 g; Carrot — 65 g; Olive oil — 15 g; Wholewheat bread — 65 g

Snack 2. *Cottage Cheese with Tomato* — Cottage cheese (low-fat) — 150 g; Tomato — 75 g

Dinner. *Greek Baked White Beans with Bread* — White beans (cooked) — 280 g; Tomato — 125 g; Carrot — 50 g; Olive oil — 15 g; Wholewheat bread — 50 g

Day 12

Breakfast. *Egg, Feta and Pepper Wrap* — Eggs — 125 g; Feta cheese — 30 g; Bell pepper — 50 g; Wholewheat pita — 65 g; Olive oil — 5 g **Snack 1.** *Apple with Almonds* — Apple — 150 g; Almonds — 15 g

Lunch. *Baked Salmon with Quinoa and Green Beans* — Salmon fillet — 165 g; Quinoa (cooked) — 205 g; Green beans — 125 g; Olive oil — 15 g; Lemon juice — 10 g

Snack 2. *Banana with Almonds* — Banana — 125 g; Almonds — 15 g

Dinner. *Grilled Pork Loin with Green Beans and Potato* — Pork loin — 165 g; Potato — 190 g; Green beans — 125 g; Olive oil — 15 g

Day 13

Breakfast. *Greek Yogurt with Pomegranate and Pumpkin Seeds* — Greek yogurt (2%) — 255 g; Pomegranate seeds — 100 g; Pumpkin seeds — 15 g; Honey — 10 g

Snack 1. *Orange with Walnuts* — Orange — 190 g; Walnuts — 15 g

Lunch. *Lentil Soup with Wholewheat Bread and Salad* — Lentils (cooked) — 320 g; Onion — 50 g; Carrot — 50 g; Celery — 40 g; Olive oil — 15 g; Wholewheat bread — 65 g; Tomato — 75 g

Snack 2. *Grapes* — Grapes — 190 g

Dinner. *Stuffed Zucchini with Rice and Beef* — Lean beef (minced) — 125 g; Brown rice (cooked) — 165 g; Zucchini — 255 g; Tomato — 75 g; Olive oil — 10 g

Day 14

Breakfast. *Wholewheat Toast with Cottage Cheese and Cucumber* — Wholewheat bread — 75 g; Cottage cheese (low-fat) — 125 g; Cucumber — 75 g; Olive oil — 5 g

Snack 1. *Greek Yogurt Cup* — Greek yogurt (2%) — 190 g

Lunch. *Greek Salad with Grilled Chicken and Pita* — Lettuce — 75 g; Tomato — 125 g; Cucumber — 100 g; Feta cheese — 50 g; Olives — 25 g; Olive oil — 15 g; Chicken breast — 125 g; Wholewheat pita — 50 g

Snack 2. *Feta with Olives and Cucumber* — Feta cheese — 40 g; Olives — 25 g; Cucumber — 65 g

Dinner. *Baked Sardines with Vegetables and Bread* — Sardines (canned) — 125 g; Tomato — 100 g; Zucchini — 100 g; Wholewheat bread — 50 g; Olive oil — 10 g

Day 15

Breakfast. *Greek Yogurt with Honey and Walnuts* — Greek yogurt (2%) — 255 g; Honey — 20 g; Walnuts — 20 g; Apple — 125 g **Snack 1.** *Grapes with Feta Cubes* — Grapes — 125 g; Feta cheese — 25 g

Lunch. *Wholewheat Pasta with Tuna and Tomato Sauce* — Wholewheat pasta (cooked) — 280 g; Tuna (canned in water) — 125 g; Tomato — 150 g; Onion — 40 g; Olive oil — 15 g

Snack 2. *Melon* — Melon — 255 g

Dinner. *Chicken and Vegetable Soup with Bread* — Chicken breast — 150 g; Carrot — 65 g; Celery — 40 g; Onion — 40 g; Wholewheat bread — 50 g; Olive oil — 10 g

Day 16

Breakfast. *Veggie Omelette with Wholewheat Toast* — Eggs — 150 g; Tomato — 75 g; Bell pepper — 40 g; Olive oil — 5 g; Wholewheat bread — 50 g

Snack 1. *Banana with Almonds* — Banana — 150 g; Almonds — 10 g

Lunch. *Beef and Vegetable Stew with Bulgur* — Lean beef — 165 g; Bulgur (cooked) — 230 g; Carrot — 65 g; Onion — 50 g; Tomato — 75 g; Olive oil — 15 g

Snack 2. *Walnuts with Figs* — Walnuts — 15 g; Fresh figs — 75 g

Dinner. *Black Bean Stew with Brown Rice* — Black beans (cooked) — 280 g; Brown rice (cooked) — 165 g; Tomato — 90 g; Onion — 40 g; Olive oil — 15 g

Day 17

Breakfast. *Oats with Milk, Banana and Almonds* — Oats — 65 g; Low-fat milk — 255 g; Banana — 125 g; Almonds — 15 g **Snack 1.** *Carrot Sticks with Hummus* — Carrot — 125 g; Hummus — 40 g

Lunch. *Shrimp with Brown Rice and Vegetables* — Shrimp — 190 g; Brown rice (cooked) — 205 g; Zucchini — 100 g; Bell pepper — 75 g; Olive oil — 15 g; Lemon juice — 10 g

Snack 2. *Strawberries with Greek Yogurt* — Strawberries — 125 g; Greek yogurt (2%) — 100 g

Dinner. *Grilled Shrimp with Couscous and Salad* — Shrimp — 180 g; Couscous (cooked) — 180 g; Lettuce — 50 g; Tomato — 75 g; Cucumber — 75 g; Olive oil — 15 g; Lemon juice — 10 g

Day 18

Breakfast. *Feta and Tomato Wholewheat Toast* — Wholewheat bread — 75 g; Feta cheese — 50 g; Tomato — 100 g; Olive oil — 5 g; Olives — 20 g **Snack 1.** *Melon Slices* — Melon — 255 g

Lunch. *White Bean Soup with Olive Oil and Bread* — White beans (cooked) — 320 g; Tomato — 100 g; Carrot — 50 g; Celery — 40 g; Olive oil — 15 g; Wholewheat bread — 65 g

Snack 2. *Pomegranate with Walnuts* — Pomegranate seeds — 125 g; Walnuts — 10 g

Dinner. *Grilled Halloumi and Vegetable Platter with Pita* — Halloumi cheese — 100 g; Zucchini — 125 g; Bell pepper — 100 g; Eggplant — 100 g; Olive oil — 15 g; Wholewheat pita — 50 g

Day 19

Breakfast. *Greek Yogurt Parfait with Strawberries* — Greek yogurt (2%) — 230 g; Strawberries — 125 g; Honey — 15 g; Sesame seeds — 15 g **Snack 1.** *Fresh Figs with Walnuts* — Fresh figs — 100 g; Walnuts — 10 g

Lunch. *Turkey Breast with Couscous and Roasted Vegetables* — Turkey breast — 180 g; Couscous (cooked) — 205 g; Zucchini — 90 g; Eggplant — 90 g; Olive oil — 15 g

Snack 2. *Mini Pita with Hummus* — Wholewheat pita — 30 g; Hummus — 30 g

Dinner. *Grilled Salmon with Roasted Vegetables and Potato* — Salmon fillet — 150 g; Potato — 190 g; Zucchini — 100 g; Bell pepper — 75 g; Olive oil — 15 g; Lemon juice — 10 g

Day 20

Breakfast. *Egg and Spinach Scramble with Bread* — Eggs — 150 g; Spinach — 75 g; Olive oil — 5 g; Wholewheat bread — 50 g **Snack 1.** *Cucumber with Yogurt Dip* — Cucumber — 125 g; Greek yogurt (2%) — 100 g

Lunch. *Baked Pork Loin with Potatoes and Green Beans* — Pork loin — 165 g; Potato — 255 g; Green beans — 125 g; Olive oil — 15 g **Snack 2.** *Kiwi with Almonds* — Kiwi — 150 g; Almonds — 15 g

Dinner. *Vegetable and Chickpea Bake* — Chickpeas (cooked) — 230 g; Eggplant — 190 g; Tomato — 125 g; Onion — 50 g; Olive oil — 15 g; Feta cheese — 40 g

Day 21

Breakfast. *Wholewheat Toast with Hummus and Cucumber* — Wholewheat bread — 75 g; Hummus — 50 g; Cucumber — 75 g; Olives — 15 g **Snack 1.** *Watermelon with Feta* — Watermelon — 190 g; Feta cheese — 25 g

Lunch. *Stuffed Peppers with Rice and Beef* — Lean beef (minced) — 150 g; Brown rice (cooked) — 190 g; Bell pepper — 190 g; Tomato — 75 g; Olive oil — 10 g

Snack 2. *Greek Yogurt with Walnuts* — Greek yogurt (2%) — 190 g; Walnuts — 15 g

Dinner. *Chicken Souvlaki with Salad and Pita* — Chicken breast — 190 g; Wholewheat pita — 65 g; Tomato — 100 g; Cucumber — 100 g; Onion — 25 g; Olive oil — 15 g; Greek yogurt (2%) — 65 g

Day 22

Breakfast. *Cinnamon Oats Cooked in Milk* — Oats — 55 g; Low-fat milk — 280 g; Honey — 15 g; Cinnamon — 2 g **Snack 1.** *Apple with Walnuts* — Apple — 165 g; Walnuts — 10 g

Lunch. *Sardines with Wholewheat Bread and Salad* — Sardines (canned) — 125 g; Wholewheat bread — 75 g; Tomato — 100 g; Cucumber — 75 g; Olive oil — 15 g; Lemon juice — 10 g

Snack 2. *Hummus with Cucumber and Carrot* — Hummus — 50 g; Cucumber — 75 g; Carrot — 75 g

Dinner. *Garlic Shrimp with Brown Rice and Spinach* — Shrimp — 180 g; Brown rice (cooked) — 180 g; Spinach — 100 g; Olive oil — 15 g; Lemon juice — 10 g

Day 23

Breakfast. *Greek Yogurt with Figs and Walnuts* — Greek yogurt (2%) — 255 g; Fresh figs — 100 g; Walnuts — 20 g **Snack 1.** *Strawberries with Almonds* — Strawberries — 190 g; Almonds — 15 g

Lunch. *Black Bean and Quinoa Bowl* — Black beans (cooked) — 280 g; Quinoa (cooked) — 190 g; Bell pepper — 75 g; Tomato — 75 g; Olive oil — 15 g; Lemon juice — 10 g

Snack 2. *Orange* — Orange — 255 g

Dinner. *Lentil and Vegetable Stew* — Lentils (cooked) — 280 g; Carrot — 65 g; Onion — 50 g; Tomato — 100 g; Olive oil — 15 g; Wholewheat bread — 40 g

Day 24

Breakfast. *Grilled Halloumi Toast with Tomato* — Wholewheat bread — 65 g; Halloumi cheese — 50 g; Tomato — 90 g; Olive oil — 5 g **Snack 1.** *Mini Pita with Hummus* — Wholewheat pita — 25 g; Hummus — 30 g

Lunch. *Grilled Chicken Breast with Brown Rice and Salad* — Chicken breast — 190 g; Brown rice (cooked) — 230 g; Tomato — 100 g; Cucumber — 100 g; Olive oil — 15 g; Lemon juice — 10 g; Lettuce — 50 g **Snack 2.** *Almonds with Apple* — Almonds — 25 g; Apple — 100 g

Dinner. *Turkey Meatballs with Tomato Sauce and Bulgur* — Turkey breast (minced) — 180 g; Bulgur (cooked) — 190 g; Tomato — 150 g; Onion — 40 g; Olive oil — 15 g

Day 25

Breakfast. *Banana Almond Breakfast Bowl* — Low-fat milk — 190 g; Banana — 150 g; Almonds — 20 g; Oats — 25 g **Snack 1.** *Kiwi with Walnuts* — Kiwi — 190 g; Walnuts — 10 g

Lunch. *Chickpea and Vegetable Stew with Wholewheat Bread* — Chickpeas (cooked) — 280 g; Tomato — 125 g; Onion — 65 g; Carrot — 65 g; Olive oil — 15 g; Wholewheat bread — 65 g

Snack 2. *Cottage Cheese with Tomato* — Cottage cheese (low-fat) — 150 g; Tomato — 75 g

Dinner. *Greek Baked White Beans with Bread* — White beans (cooked) — 280 g; Tomato — 125 g; Carrot — 50 g; Olive oil — 15 g; Wholewheat bread — 50 g

Day 26

Breakfast. *Egg, Feta and Pepper Wrap* — Eggs — 125 g; Feta cheese — 30 g; Bell pepper — 50 g; Wholewheat pita — 65 g; Olive oil — 5 g **Snack 1.** *Apple with Almonds* — Apple — 150 g; Almonds — 15 g

Lunch. *Baked Salmon with Quinoa and Green Beans* — Salmon fillet — 165 g; Quinoa (cooked) — 205 g; Green beans — 125 g; Olive oil — 15 g; Lemon juice — 10 g

Snack 2. *Banana with Almonds* — Banana — 125 g; Almonds — 15 g

Dinner. *Grilled Pork Loin with Green Beans and Potato* — Pork loin — 165 g; Potato — 190 g; Green beans — 125 g; Olive oil — 15 g

Day 27

Breakfast. *Greek Yogurt with Pomegranate and Pumpkin Seeds* — Greek yogurt (2%) — 255 g; Pomegranate seeds — 100 g; Pumpkin seeds — 15 g; Honey — 10 g

Snack 1. *Orange with Walnuts* — Orange — 190 g; Walnuts — 15 g

Lunch. *Lentil Soup with Wholewheat Bread and Salad* — Lentils (cooked) — 320 g; Onion — 50 g; Carrot — 50 g; Celery — 40 g; Olive oil — 15 g; Wholewheat bread — 65 g; Tomato — 75 g

Snack 2. *Grapes* — Grapes — 190 g

Dinner. *Stuffed Zucchini with Rice and Beef* — Lean beef (minced) — 125 g; Brown rice (cooked) — 165 g; Zucchini — 255 g; Tomato — 75 g; Olive oil — 10 g

Day 28

Breakfast. *Wholewheat Toast with Cottage Cheese and Cucumber* — Wholewheat bread — 75 g; Cottage cheese (low-fat) — 125 g; Cucumber — 75 g; Olive oil — 5 g

Snack 1. *Greek Yogurt Cup* — Greek yogurt (2%) — 190 g

Lunch. *Greek Salad with Grilled Chicken and Pita* — Lettuce — 75 g; Tomato — 125 g; Cucumber — 100 g; Feta cheese — 50 g; Olives — 25 g; Olive oil — 15 g; Chicken breast — 125 g; Wholewheat pita — 50 g

Snack 2. *Feta with Olives and Cucumber* — Feta cheese — 40 g; Olives — 25 g; Cucumber — 65 g

Dinner. *Baked Sardines with Vegetables and Bread* — Sardines (canned) — 125 g; Tomato — 100 g; Zucchini — 100 g; Wholewheat bread — 50 g; Olive oil — 10 g

5.2. Comparison with the MAI-DIET plan

The language-model plan reported a mean daily energy of 2004.5 kcal (+0.2 % of target) with a standard deviation of 131.3 kcal and a daily range of 1794 to 2253 kcal. The MAI-DIET plan for the same profile reported 2032.6 kcal (+1.6 %) with a standard deviation of 46.3 kcal. The language-model plan met 10 of the thirteen reference criteria and the MAI-DIET plan met 10. Day-to-day dispersion was 2.8 times greater in the language-model plan, so that individual days departed from the daily tolerance band even though the 28-day mean sat close to the target. Sodium was 1273.0 mg/day against 2555.9 mg/day for MAI-DIET, and calcium 875.4 mg/day against 1048.6 mg/day. Protein was 126.8 g/day against 106.7 g/day, both above the reference range for this profile. The plan contained 68 unique dishes across 140 meal slots.

5.3. Compliance heat map

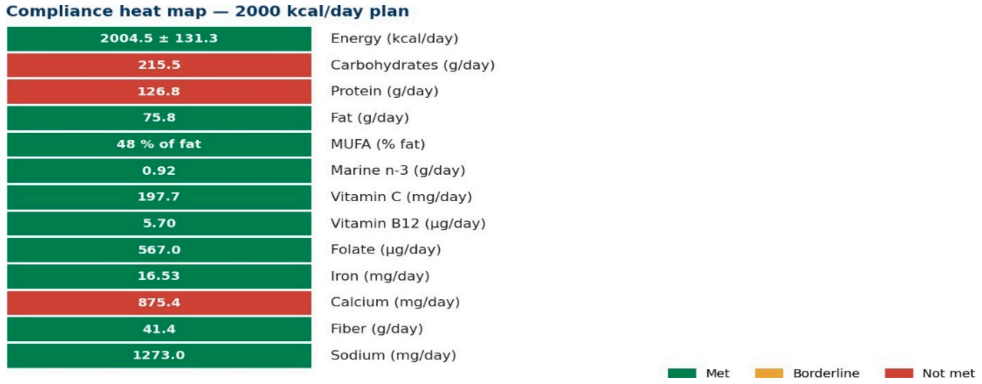

Figure S4. Compliance of the 2000 kcal/day language-model plan against the thirteen reference criteria applied in **Supplementary Table S7**. Values shown within each cell are the mean daily values reported by the model.

6. Plan 4 — 2200 kcal/day

**Sex**	Female	**Age**	35 y	**Height**	168 cm
**Body mass**	75 kg	**Activity**	1.55 (moderately active)	**Goal**	Weight maintenance

6.1. Twenty-eight-day meal plan

Day 1

Breakfast. *Greek yogurt bowl with honey and walnuts* — Greek yogurt 2% fat — 290 g; Honey — 20 g; Walnuts — 20 g; Strawberries — 105 g **Snack 1.** *Fresh fruit* — Orange — 200 g

Lunch. *Grilled chicken souvlaki with Greek salad* — Chicken breast — 200 g; Extra virgin olive oil — 15 g; Tomato — 160 g; Cucumber — 130 g; Red onion — 40 g; Feta cheese — 55 g; Kalamata olives — 20 g; Whole wheat bread — 65 g

Snack 2. *Hummus with vegetable sticks* — Hummus — 80 g; Carrot — 105 g; Cucumber — 80 g

Dinner. *Baked sea bream with roasted vegetables* — Sea bream fillet — 240 g; Zucchini — 160 g; Bell pepper — 130 g; Extra virgin olive oil — 15 g; Lemon juice — 15 g; Brown rice (cooked) — 160 g

Day 2

Breakfast. *Whole wheat toast with egg and tomato* — Whole wheat bread — 90 g; Egg — 130 g; Tomato — 105 g; Extra virgin olive oil — 10 g; Feta cheese — 25 g

Snack 1. *Yogurt with fruit* — Greek yogurt 2% fat — 200 g; Banana — 130 g

Lunch. *Lentil soup (fakes) with bread* — Lentils (cooked) — 370 g; Carrot — 65 g; Red onion — 55 g; Extra virgin olive oil — 20 g; Whole wheat bread — 65 g; Lemon juice — 15 g

Snack 2. *Almonds and dried apricots* — Almonds — 25 g; Dried apricots — 35 g

Dinner. *Grilled chicken thigh with bulgur and greens* — Chicken thigh, skinless — 200 g; Bulgur (cooked) — 200 g; Wild greens (horta) — 160 g; Extra virgin olive oil — 15 g; Lemon juice — 10 g

Day 3

Breakfast. *Oatmeal with milk, banana and cinnamon* — Oats (dry) — 65 g; Low-fat milk 1.5% — 265 g; Banana — 130 g; Cinnamon — 3 g **Snack 1.** *Handful of pistachios* — Pistachios — 35 g

Lunch. *Chickpea stew (revithada) with salad* — Chickpeas (cooked) — 330 g; Tomato — 130 g; Red onion — 55 g; Extra virgin olive oil — 20 g; Lettuce — 80 g; Cucumber — 80 g

Snack 2. *Fresh fruit* — Apple — 200 g

Dinner. *Baked salmon with quinoa and broccoli* — Salmon fillet — 200 g; Quinoa (cooked) — 170 g; Broccoli — 160 g; Extra virgin olive oil — 15 g; Lemon juice — 10 g

Day 4

Breakfast. *Greek yogurt with oats and figs* — Greek yogurt 2% fat — 265 g; Oats (dry) — 40 g; Fresh figs — 105 g; Honey — 15 g

Snack 1. *Carrot and cucumber sticks with tzatziki-style yogurt* — Greek yogurt 2% fat — 80 g; Carrot — 90 g; Cucumber — 90 g; Garlic — 4 g **Lunch.** *Whole wheat pasta with tomato sauce and feta* — Whole wheat pasta (cooked) — 330 g; Tomato — 200 g; Extra virgin olive oil — 20 g; Feta cheese — 55 g; Garlic — 7 g; Parsley — 7 g

Snack 2. *Fresh fruit* — Grapes — 170 g

Dinner. *Grilled shrimp with fennel-cucumber salad* — Shrimp — 240 g; Fennel bulb — 130 g; Cucumber — 130 g; Extra virgin olive oil — 15 g; Lemon juice — 15 g; Whole wheat bread — 65 g

Day 5

Breakfast. *Whole grain rusks with cottage cheese and tomato* — Whole grain rusks (paximadi) — 80 g; Cottage cheese low-fat — 130 g; Tomato — 130 g; Extra virgin olive oil — 10 g

Snack 1. *Fresh fruit* — Pear — 200 g

Lunch. *Fasolada (white bean soup) with bread* — White beans (cooked) — 370 g; Carrot — 80 g; Celery — 55 g;

Tomato — 80 g; Extra virgin olive oil — 20 g; Whole wheat bread — 65 g

Snack 2. *Walnuts* — Walnuts — 25 g

Dinner. *Grilled turkey breast with roasted potatoes and greens* — Turkey breast — 200 g; Potato (boiled) — 240 g; Wild greens (horta) — 130 g; Extra virgin olive oil — 15 g; Lemon juice — 10 g

Day 6

Breakfast. *Two-egg omelette with spinach and feta* — Egg — 160 g; Spinach (raw) — 80 g; Feta cheese — 40 g; Extra virgin olive oil — 10 g; Whole wheat bread — 65 g

Snack 1. *Fresh fruit* — Mandarin/clementine — 200 g

Lunch. *Octopus with white bean salad* — Octopus (cooked) — 200 g; White beans (cooked) — 240 g; Red onion — 40 g; Extra virgin olive oil — 20 g; Lemon juice — 15 g; Parsley — 7 g

Snack 2. *Greek yogurt with honey* — Greek yogurt 2% fat — 160 g; Honey — 15 g

Dinner. *Chicken and vegetable bulgur pilaf* — Chicken breast — 160 g; Bulgur (cooked) — 200 g; Zucchini — 105 g; Bell pepper — 105 g; Extra virgin olive oil — 15 g

Day 7

Breakfast. *Greek yogurt with pistachios and pomegranate* — Greek yogurt 2% fat — 290 g; Pistachios — 20 g; Pomegranate arils — 80 g **Snack 1.** *Whole grain crackers with cheese* — Whole grain crackers — 40 g; Anthotyro cheese (soft) — 55 g

Lunch. *Grilled sea bream with briam (roasted vegetables)* — Sea bream fillet — 225 g; Zucchini — 130 g; Eggplant — 130 g; Tomato — 105 g; Extra virgin olive oil — 20 g; Whole wheat bread — 55 g

Snack 2. *Fresh fruit* — Watermelon — 240 g

Dinner. *Lentil and vegetable stew* — Lentils (cooked) — 345 g; Carrot — 80 g; Celery — 55 g; Tomato — 80 g; Extra virgin olive oil — 15 g; Lemon juice — 10 g

Day 8

Breakfast. *Oatmeal with walnuts and honey* — Oats (dry) — 65 g; Low-fat milk 1.5% — 240 g; Walnuts — 20 g; Honey — 15 g **Snack 1.** *Fresh fruit* — Kiwi — 200 g

Lunch. *Chicken souvlaki wrap with vegetables* — Chicken breast — 200 g; Whole wheat bread — 90 g; Tomato — 105 g; Cucumber — 80 g; Greek yogurt 2% fat — 55 g; Extra virgin olive oil — 10 g

Snack 2. *Almonds* — Almonds — 35 g

Dinner. *Baked mackerel with steamed greens and potatoes* — Mackerel fillet — 200 g; Wild greens (horta) — 130 g; Potato (boiled) — 200 g; Extra virgin olive oil — 15 g; Lemon juice — 10 g

Day 9

Breakfast. *Whole wheat toast with tahini and honey* — Whole wheat bread — 90 g; Tahini — 25 g; Honey — 15 g; Banana — 105 g **Snack 1.** *Yogurt with fruit* — Greek yogurt 2% fat — 200 g; Strawberries — 130 g

Lunch. *Chickpea and vegetable salad with tuna* — Chickpeas (cooked) — 265 g; Tuna canned in water, drained — 105 g; Tomato — 130 g; Cucumber — 105 g; Red onion — 40 g; Extra virgin olive oil — 20 g; Lemon juice — 10 g

Snack 2. *Fresh fruit* — Fresh figs — 130 g

Dinner. *Pork tenderloin with roasted vegetables* — Pork tenderloin — 200 g; Zucchini — 130 g; Bell pepper — 130 g; Carrot — 105 g; Extra virgin olive oil — 15 g

Day 10

Breakfast. *Greek yogurt with granola-style oats and apple* — Greek yogurt 2% fat — 265 g; Oats (dry) — 35 g; Apple — 130 g; Cinnamon — 1 g **Snack 1.** *Hummus with cucumber* — Hummus — 80 g; Cucumber — 105 g

Lunch. *Baked chicken with orzo-bulgur and salad* — Chicken breast — 200 g; Bulgur (cooked) — 200 g; Lettuce — 80 g; Tomato — 90 g; Extra virgin olive oil — 15 g; Lemon juice — 10 g

Snack 2. *Fresh fruit* — Grapes — 160 g

Dinner. *Grilled shrimp with quinoa tabbouleh* — Shrimp — 225 g; Quinoa (cooked) — 160 g; Parsley — 40 g; Tomato — 90 g; Extra virgin olive oil — 15 g; Lemon juice — 15 g

Day 11

Breakfast. *Egg and vegetable scramble with bread* — Egg — 160 g; Bell pepper — 80 g; Red onion — 40 g; Extra virgin olive oil — 10 g; Whole wheat bread — 80 g

Snack 1. *Fresh fruit* — Orange — 200 g

Lunch. *Fasolada with feta and olives* — White beans (cooked) — 345 g; Carrot — 65 g; Celery — 40 g; Feta cheese — 40 g; Kalamata olives — 20 g; Extra virgin olive oil — 15 g

Snack 2. *Walnuts and dried apricots* — Walnuts — 20 g; Dried apricots — 25 g

Dinner. *Grilled salmon with green beans and potatoes* — Salmon fillet — 200 g; Green beans — 160 g; Potato (boiled) — 170 g; Extra virgin olive oil — 15 g; Lemon juice — 10 g

Day 12

Breakfast. *Whole grain rusks with anthotyro and tomato* — Whole grain rusks (paximadi) — 75 g; Anthotyro cheese (soft) — 65 g; Tomato — 120 g; Extra virgin olive oil — 10 g

Snack 1. *Fresh fruit* — Melon (cantaloupe) — 240 g

Lunch. *Turkey breast with brown rice and vegetables* — Turkey breast — 200 g; Brown rice (cooked) — 200 g; Carrot — 80 g; Broccoli — 130 g; Extra virgin olive oil — 15 g

Snack 2. *Greek yogurt with honey* — Greek yogurt 2% fat — 170 g; Honey — 15 g

Dinner. *Octopus and lentil salad* — Octopus (cooked) — 170 g; Lentils (cooked) — 265 g; Red onion — 40 g; Extra virgin olive oil — 15 g; Lemon juice — 15 g; Parsley — 7 g

Day 13

Breakfast. *Oatmeal with mixed berries and almonds* — Oats (dry) — 65 g; Low-fat milk 1.5% — 240 g; Strawberries — 105 g; Almonds — 20 g **Snack 1.** *Fresh fruit* — Pear — 200 g

Lunch. *Grilled chicken with Greek salad and bread* — Chicken breast — 200 g; Tomato — 160 g; Cucumber — 130 g; Feta cheese — 55 g; Kalamata olives — 20 g; Extra virgin olive oil — 20 g; Whole wheat bread — 55 g

Snack 2. *Whole grain crackers with hummus* — Whole grain crackers — 35 g; Hummus — 65 g

Dinner. *Baked cod-style sea bream with cauliflower rice-style bulgur* — Sea bream fillet — 225 g; Cauliflower — 130 g; Bulgur (cooked) — 160 g; Extra virgin olive oil — 15 g; Lemon juice — 10 g

Day 14

Breakfast. *Greek yogurt with dark chocolate shavings and banana* — Greek yogurt 2% fat — 265 g; Dark chocolate 70% — 15 g; Banana — 130 g **Snack 1.** *Fresh fruit* — Mandarin/clementine — 200 g

Lunch. *Chickpea and spinach stew with bread* — Chickpeas (cooked) — 330 g; Spinach (raw) — 130 g; Tomato — 105 g; Extra virgin olive oil — 20 g; Whole wheat bread — 65 g

Snack 2. *Almonds* — Almonds — 25 g

Dinner. *Grilled pork tenderloin with green beans and potatoes* — Pork tenderloin — 200 g; Green beans — 160 g; Potato (boiled) — 170 g; Extra virgin olive oil — 15 g; Lemon juice — 10 g

Day 15

Breakfast. *Whole wheat toast with egg and avocado* — Whole wheat bread — 80 g; Egg — 130 g; Avocado — 65 g; Tomato — 80 g **Snack 1.** *Yogurt with fruit* — Greek yogurt 2% fat — 200 g; Kiwi — 130 g

Lunch. *Baked chicken souvlaki with bulgur pilaf* — Chicken breast — 200 g; Bulgur (cooked) — 200 g; Zucchini — 105 g; Extra virgin olive oil — 15 g; Lemon juice — 10 g

Snack 2. *Fresh fruit* — Fresh figs — 120 g

Dinner. *Sardines with horiatiki-style salad* — Sardines canned in olive oil, drained — 160 g; Tomato — 160 g; Cucumber — 130 g; Red onion — 40 g; Kalamata olives — 20 g; Whole wheat bread — 55 g

Day 16

Breakfast. *Oatmeal with tahini and dried apricots* — Oats (dry) — 65 g; Low-fat milk 1.5% — 240 g; Tahini — 15 g; Dried apricots — 35 g **Snack 1.** *Fresh fruit* — Apple — 200 g

Lunch. *Lentil soup with feta and bread* — Lentils (cooked) — 345 g; Carrot — 65 g; Feta cheese — 40 g; Extra virgin olive oil — 20 g; Whole wheat bread — 65 g

Snack 2. *Pistachios* — Pistachios — 25 g

Dinner. *Grilled shrimp with roasted vegetables and quinoa* — Shrimp — 225 g; Zucchini — 130 g; Bell pepper — 120 g; Quinoa (cooked) — 130 g; Extra virgin olive oil — 15 g; Lemon juice — 10 g

Day 17

Breakfast. *Greek yogurt bowl with walnuts and honey* — Greek yogurt 2% fat — 290 g; Walnuts — 20 g; Honey — 20 g; Grapes — 90 g **Snack 1.** *Carrot sticks with hummus* — Carrot — 105 g; Hummus — 65 g

Lunch. *Baked sea bream with roasted potatoes and greens* — Sea bream fillet — 240 g; Potato (boiled) — 200 g; Wild greens (horta) — 130 g; Extra virgin olive oil — 20 g; Lemon juice — 15 g

Snack 2. *Fresh fruit* — Orange — 200 g

Dinner. *Turkey breast with cauliflower and brown rice* — Turkey breast — 200 g; Cauliflower — 160 g; Brown rice (cooked) — 160 g; Extra virgin olive oil — 15 g

Day 18

Breakfast. *Whole grain rusks with cottage cheese and cucumber* — Whole grain rusks (paximadi) — 75 g; Cottage cheese low-fat — 130 g; Cucumber — 105 g; Extra virgin olive oil — 10 g

Snack 1. *Fresh fruit* — Banana — 160 g

Lunch. *White bean and vegetable salad with tuna* — White beans (cooked) — 290 g; Tuna canned in water, drained — 105 g; Tomato — 120 g; Red onion — 40 g; Extra virgin olive oil — 20 g; Lemon juice — 10 g

Snack 2. *Dried apricots and almonds* — Dried apricots — 25 g; Almonds — 20 g

Dinner. *Chicken thigh with eggplant and bulgur* — Chicken thigh, skinless — 200 g; Eggplant — 160 g; Bulgur (cooked) — 170 g; Extra virgin olive oil — 15 g; Garlic — 4 g

Day 19

Breakfast. *Egg and mushroom scramble with bread* — Egg — 160 g; Mushrooms — 90 g; Extra virgin olive oil — 10 g; Whole wheat bread — 80 g **Snack 1.** *Yogurt with fruit* — Greek yogurt 2% fat — 200 g; Strawberries — 120 g

Lunch. *Grilled chicken with quinoa tabbouleh* — Chicken breast — 200 g; Quinoa (cooked) — 170 g; Parsley — 35 g; Tomato — 90 g; Extra virgin olive oil — 15 g; Lemon juice — 10 g

Snack 2. *Fresh fruit* — Watermelon — 240 g

Dinner. *Baked salmon with steamed broccoli and potatoes* — Salmon fillet — 200 g; Broccoli — 145 g; Potato (boiled) — 170 g; Extra virgin olive oil — 15 g; Lemon juice — 10 g

Day 20

Breakfast. *Greek yogurt with figs and pistachios* — Greek yogurt 2% fat — 275 g; Fresh figs — 120 g; Pistachios — 20 g **Snack 1.** *Whole grain crackers with cheese* — Whole grain crackers — 40 g; Anthotyro cheese (soft) — 45 g

Lunch. *Octopus with fennel-cucumber salad and bread* — Octopus (cooked) — 200 g; Fennel bulb — 120 g; Cucumber — 120 g; Extra virgin olive oil — 20 g; Lemon juice — 15 g; Whole wheat bread — 55 g

Snack 2. *Fresh fruit* — Pear — 185 g

Dinner. *Chickpea and vegetable stew* — Chickpeas (cooked) — 345 g; Zucchini — 130 g; Tomato — 105 g; Extra virgin olive oil — 15 g; Lemon juice — 7 g

Day 21

Breakfast. *Oatmeal with banana and cinnamon* — Oats (dry) — 75 g; Low-fat milk 1.5% — 250 g; Banana — 130 g; Cinnamon — 3 g **Snack 1.** *Fresh fruit* — Kiwi — 185 g

Lunch. *Grilled pork tenderloin with Greek salad* — Pork tenderloin — 200 g; Tomato — 145 g; Cucumber — 130 g; Feta cheese — 45 g; Kalamata olives — 20 g; Extra virgin olive oil — 15 g; Whole wheat bread — 55 g

Snack 2. *Almonds* — Almonds — 30 g

Dinner. *Baked mackerel with lentil salad* — Mackerel fillet — 200 g; Lentils (cooked) — 240 g; Red onion — 35 g; Extra virgin olive oil — 15 g; Lemon juice — 10 g

Day 22

Breakfast. *Whole wheat toast with tahini and banana* — Whole wheat bread — 90 g; Tahini — 25 g; Banana — 120 g **Snack 1.** *Yogurt with fruit* — Greek yogurt 2% fat — 200 g; Grapes — 130 g

Lunch. *Chicken breast with brown rice and green beans* — Chicken breast — 200 g; Brown rice (cooked) — 200 g; Green beans — 145 g; Extra virgin olive oil — 15 g

Snack 2. *Fresh fruit* — Mandarin/clementine — 200 g

Dinner. *Shrimp with roasted vegetables and bulgur* — Shrimp — 225 g; Eggplant — 130 g; Bell pepper — 120 g; Bulgur (cooked) — 145 g; Extra virgin olive oil — 15 g; Lemon juice — 10 g

Day 23

Breakfast. *Greek yogurt with honey and almonds* — Greek yogurt 2% fat — 290 g; Honey — 20 g; Almonds — 25 g

Snack 1. *Carrot and cucumber sticks with tzatziki-style yogurt* — Greek yogurt 2% fat — 80 g; Carrot — 90 g; Cucumber — 90 g; Garlic — 4 g **Lunch.** *Baked chicken with roasted potatoes and horta* — Chicken breast — 200 g; Potato (boiled) — 210 g; Wild greens (horta) — 130 g; Extra virgin olive oil — 15 g; Lemon juice — 10 g

Snack 2. *Fresh fruit* — Fresh figs — 130 g

Dinner. *Sardines with quinoa and tomato salad* — Sardines canned in olive oil, drained — 160 g; Quinoa (cooked) — 145 g; Tomato — 130 g; Extra virgin olive oil — 10 g; Lemon juice — 10 g

Day 24

Breakfast. *Egg and spinach wrap* — Egg — 145 g; Spinach (raw) — 80 g; Whole wheat bread — 85 g; Extra virgin olive oil — 10 g; Feta cheese — 35 g

Snack 1. *Fresh fruit* — Apple — 200 g

Lunch. *White bean salad with sardines* — White beans (cooked) — 290 g; Sardines canned in olive oil, drained — 105 g; Tomato — 120 g; Red onion — 40 g; Extra virgin olive oil — 15 g; Lemon juice — 10 g

Snack 2. *Walnuts* — Walnuts — 25 g

Dinner. *Turkey meatball-style breast with zucchini and bulgur* — Turkey breast — 200 g; Zucchini — 160 g; Bulgur (cooked) — 170 g; Extra virgin olive oil — 15 g; Garlic — 4 g

Day 25

Breakfast. *Oatmeal with strawberries and walnuts* — Oats (dry) — 65 g; Low-fat milk 1.5% — 240 g; Strawberries — 120 g; Walnuts — 20 g **Snack 1.** *Fresh fruit* — Orange — 200 g

Lunch. *Grilled sea bream with fennel and potatoes* — Sea bream fillet — 240 g; Fennel bulb — 120 g; Potato (boiled) — 170 g; Extra virgin olive oil — 15 g; Lemon juice — 15 g

Snack 2. *Hummus with cucumber and carrot* — Hummus — 75 g; Cucumber — 80 g; Carrot — 65 g

Dinner. *Lentil stew with feta and bread* — Lentils (cooked) — 345 g; Carrot — 65 g; Feta cheese — 40 g; Extra virgin olive oil — 15 g; Whole wheat bread — 60 g

Day 26

Breakfast. *Greek yogurt with pomegranate and pistachios* — Greek yogurt 2% fat — 275 g; Pomegranate arils — 90 g; Pistachios — 20 g **Snack 1.** *Fresh fruit* — Pear — 200 g

Lunch. *Chicken souvlaki plate with bulgur and salad* — Chicken breast — 200 g; Bulgur (cooked) — 170 g; Lettuce — 80 g; Tomato — 90 g; Extra virgin olive oil — 15 g; Lemon juice — 10 g

Snack 2. *Fresh fruit* — Grapes — 160 g

Dinner. *Baked salmon with cauliflower and quinoa* — Salmon fillet — 200 g; Cauliflower — 145 g; Quinoa (cooked) — 145 g; Extra virgin olive oil — 15 g; Lemon juice — 10 g

Day 27

Breakfast. *Whole grain rusks with anthotyro, olives and tomato* — Whole grain rusks (paximadi) — 75 g; Anthotyro cheese (soft) — 60 g; Kalamata olives — 20 g; Tomato — 120 g; Extra virgin olive oil — 8 g

Snack 1. *Yogurt with fruit* — Greek yogurt 2% fat — 200 g; Banana — 130 g

Lunch. *Octopus and chickpea salad* — Octopus (cooked) — 200 g; Chickpeas (cooked) — 265 g; Red onion — 40 g; Extra virgin olive oil — 15 g; Lemon juice — 15 g; Parsley — 7 g

Snack 2. *Almonds and dried apricots* — Almonds — 25 g; Dried apricots — 25 g

Dinner. *Pork tenderloin with green beans and brown rice* — Pork tenderloin — 200 g; Green beans — 160 g; Brown rice (cooked) — 170 g; Extra virgin olive oil — 15 g

Day 28

Breakfast. *Oatmeal with honey, banana and almonds* — Oats (dry) — 75 g; Low-fat milk 1.5% — 250 g; Honey — 15 g; Banana — 120 g; Almonds — 15 g

Snack 1. *Fresh fruit* — Melon (cantaloupe) — 240 g

Lunch. *Grilled turkey breast with Greek salad and bread* — Turkey breast — 200 g; Tomato — 145 g; Cucumber — 130 g; Feta cheese — 45 g; Kalamata olives — 20 g; Extra virgin olive oil — 15 g; Whole wheat bread — 55 g

Snack 2. *Whole grain crackers with hummus* — Whole grain crackers — 35 g; Hummus — 65 g

Dinner. *Baked mackerel with roasted vegetables and bulgur* — Mackerel fillet — 200 g; Zucchini — 130 g; Bell pepper — 120 g; Bulgur (cooked) — 160 g; Extra virgin olive oil — 15 g; Lemon juice — 10 g

6.2. Comparison with the MAI-DIET plan

The language-model plan reported a mean daily energy of 2208.2 kcal (+0.4 % of target) with a standard deviation of 183.9 kcal and a daily range of 1892 to 2613 kcal. The MAI-DIET plan for the same profile reported 2234.4 kcal (+1.6 %) with a standard deviation of 50.1 kcal. The language-model plan met 10 of the thirteen reference criteria and the MAI-DIET plan met 10. Day-to-day dispersion was 3.7 times greater in the language-model plan, so that individual days departed from the daily tolerance band even though the 28-day mean sat close to the target. Sodium was 1193.4 mg/day against 2873.0 mg/day for MAI-DIET, and calcium 872.0 mg/day against 1195.2 mg/day. Protein was 154.6 g/day against 117.0 g/day, both above the reference range for this profile. The plan contained 101 unique dishes across 140 meal slots.

6.3. Compliance heat map

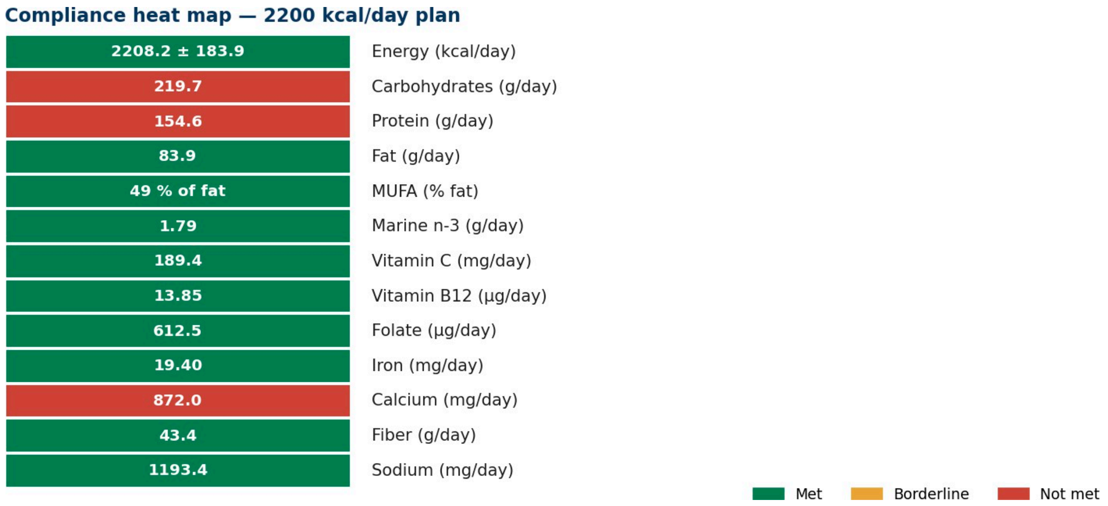

Figure S5. Compliance of the 2200 kcal/day language-model plan against the thirteen reference criteria applied in **Supplementary Table S7**. Values shown within each cell are the mean daily values reported by the model.

7. Plan 5 — 2400 kcal/day

**Sex**	Female	**Age**	28 y	**Height**	165 cm
**Body mass**	64 kg	**Activity**	1.725 (very active)	**Goal**	Weight maintenance

7.1. Twenty-eight-day meal plan

Day 1

Breakfast. *Greek yogurt with honey and walnuts* — Greek yogurt (2% fat) — 323.2 g; Honey — 25.9 g; Walnuts — 25.9 g; Strawberries — 103.4 g **Snack 1.** *Apple with almonds* — Apple — 193.9 g; Almonds — 19.4 g

Lunch. *Chickpea and tomato stew with bread* — Chickpeas, cooked — 387.8 g; Tomato — 129.3 g; Olive oil — 15.5 ml; Wholegrain bread — 64.6 g **Snack 2.** *Wholegrain crackers with feta cheese* — Wholegrain crackers — 38.8 g; Feta cheese — 32.3 g

Dinner. *Turkey meatballs with tomato sauce and pasta* — Turkey breast, roasted — 193.9 g; Egg — 38.8 g; Wholegrain pasta, cooked — 258.5 g; Tomato — 155.1 g; Olive oil — 12.9 ml

Day 2

Breakfast. *Vegetable and feta omelette with wholegrain bread* — Egg — 155.1 g; Spinach — 51.7 g; Tomato — 64.6 g; Feta cheese — 38.8 g; Olive oil — 6.5 ml; Wholegrain bread — 51.7 g

Snack 1. *Greek yogurt with honey* — Greek yogurt (2% fat) — 193.9 g; Honey — 12.9 g

Lunch. *Wholegrain pasta with tuna and tomato* — Wholegrain pasta, cooked — 323.2 g; Tuna, canned in water — 129.3 g; Tomato — 129.3 g; Olive oil — 12.9 ml

Snack 2. *Walnuts and dried apricots* — Walnuts — 25.9 g; Dried apricots — 32.3 g

Dinner. *Octopus with boiled potatoes and lemon* — Octopus, cooked — 193.9 g; Potato, boiled — 232.7 g; Olive oil — 12.9 ml; Lemon juice — 12.9 ml

Day 3

Breakfast. *Oat porridge with milk, banana and almonds* — Rolled oats — 64.6 g; Low-fat milk — 258.5 ml; Banana — 129.3 g; Almonds — 19.4 g **Snack 1.** *Orange with walnuts* — Orange — 193.9 g; Walnuts — 19.4 g

Lunch. *Baked salmon with quinoa and zucchini* — Salmon, grilled — 193.9 g; Quinoa, cooked — 258.5 g; Zucchini — 129.3 g; Olive oil — 12.9 ml **Snack 2.** *Yogurt with honey and sesame seeds* — Greek yogurt (2% fat) — 193.9 g; Honey — 12.9 g; Sesame seeds — 12.9 g

Dinner. *Lentil and vegetable stew with feta* — Lentils, cooked — 387.8 g; Carrot — 77.6 g; Tomato — 129.3 g; Feta cheese — 38.8 g; Olive oil — 12.9 ml

Day 4

Breakfast. *Wholegrain toast with hummus, tomato, cucumber and egg* — Wholegrain bread — 77.6 g; Hummus — 51.7 g; Tomato — 77.6 g; Cucumber — 77.6 g; Egg — 71.1 g

Snack 1. *Wholegrain crackers with feta* — Wholegrain crackers — 38.8 g; Feta cheese — 25.9 g

Lunch. *White bean salad with olive oil and bread* — White beans, cooked — 387.8 g; Tomato — 103.4 g; Red onion — 38.8 g; Olive oil — 19.4 ml; Wholegrain bread — 51.7 g

Snack 2. *Watermelon with feta cheese* — Watermelon — 258.5 g; Feta cheese — 38.8 g

Dinner. *Baked sardines with tomato, zucchini and bread* — Sardines, canned — 155.1 g; Tomato — 129.3 g; Zucchini — 129.3 g; Olive oil — 15.5 ml; Wholegrain bread — 64.6 g

Day 5

Breakfast. *Greek yogurt parfait with oats, honey and figs* — Greek yogurt (2% fat) — 258.5 g; Rolled oats — 38.8 g; Honey — 19.4 g; Fresh fig — 103.4 g

Snack 1. *Grapes with almonds* — Grapes — 155.1 g; Almonds — 15.5 g

Lunch. *Chicken souvlaki plate with pita and yogurt* — Chicken breast, grilled — 193.9 g; Wholegrain bread —

90.5 g; Tomato — 77.6 g; Cucumber
— 77.6 g; Greek yogurt (2% fat) — 77.6 g; Olive oil — 10.3 ml

Snack 2. *Cottage cheese with pomegranate* — Cottage cheese (low fat) — 193.9 g; Pomegranate seeds — 77.6 g

Dinner. *Mussels in tomato sauce with bread* — Mussels, cooked — 258.5 g; Tomato — 193.9 g; Olive oil — 15.5 ml; Wholegrain bread — 64.6 g

Day 6

Breakfast. *Feta and tomato sandwich with olives* — Wholegrain bread — 90.5 g; Feta cheese — 51.7 g; Tomato — 77.6 g; Kalamata olives — 25.9 g; Olive oil — 6.5 ml

Snack 1. *Carrot sticks with hummus* — Carrot — 129.3 g; Hummus — 51.7 g

Lunch. *Octopus with white beans and spinach* — Octopus, cooked — 168 g; White beans, cooked — 258.5 g; Spinach — 77.6 g; Olive oil — 15.5 ml **Snack 2.** *Greek yogurt with pumpkin seeds* — Greek yogurt (2% fat) — 193.9 g; Pumpkin seeds — 19.4 g

Dinner. *Grilled chicken with roasted vegetables* — Chicken breast, grilled — 219.8 g; Eggplant — 129.3 g; Zucchini — 129.3 g; Potato, boiled — 129.3 g; Olive oil — 15.5 ml

Day 7

Breakfast. *Egg and roasted pepper plate with bread* — Egg — 142.2 g; Bell pepper — 77.6 g; Wholegrain bread — 51.7 g; Olive oil — 6.5 ml **Snack 1.** *Banana with almonds* — Banana — 129.3 g; Almonds — 15.5 g

Lunch. *Turkey breast with brown rice and peppers* — Turkey breast, roasted — 193.9 g; Brown rice, cooked — 258.5 g; Bell pepper — 103.4 g; Olive oil — 12.9 ml

Snack 2. *Mixed fruit salad* — Apple — 103.4 g; Orange — 103.4 g; Grapes — 77.6 g

Dinner. *Baked salmon with lemon potatoes and spinach* — Salmon, grilled — 206.8 g; Potato, boiled — 258.5 g; Spinach — 77.6 g; Olive oil — 12.9 ml; Lemon juice — 12.9 ml

Day 8

Breakfast. *Fruit and walnut yogurt bowl* — Greek yogurt (2% fat) — 284.4 g; Walnuts — 19.4 g; Honey — 19.4 g; Orange — 129.3 g **Snack 1.** *Dried apricots with walnuts* — Dried apricots — 38.8 g; Walnuts — 19.4 g

Lunch. *Grilled chicken breast with brown rice and salad* — Chicken breast, grilled — 193.9 g; Brown rice, cooked — 258.5 g; Tomato — 103.4 g; Cucumber — 103.4 g; Olive oil — 12.9 ml; Lemon juice — 12.9 ml

Snack 2. *Hummus with carrot and cucumber* — Hummus — 77.6 g; Carrot — 103.4 g; Cucumber — 77.6 g

Dinner. *Stuffed peppers with rice and feta* — Bell pepper — 258.5 g; Brown rice, cooked — 193.9 g; Tomato — 129.3 g; Feta cheese — 51.7 g; Olive oil — 15.5 ml

Day 9

Breakfast. *Savoury yogurt with cucumber, garlic and bread* — Greek yogurt (2% fat) — 258.5 g; Cucumber — 103.4 g; Wholegrain bread — 64.6 g; Olive oil — 6.5 ml; Garlic — 3.9 g

Snack 1. *Apple with almonds* — Apple — 193.9 g; Almonds — 19.4 g

Lunch. *Lentil soup with wholegrain bread* — Lentils, cooked — 452.4 g; Carrot — 64.6 g; Olive oil — 12.9 ml; Wholegrain bread — 64.6 g **Snack 2.** *Wholegrain crackers with feta cheese* — Wholegrain crackers — 38.8 g; Feta cheese — 32.3 g

Dinner. *Chickpea and spinach stew with bread* — Chickpeas, cooked — 387.8 g; Spinach — 129.3 g; Tomato — 129.3 g; Olive oil — 15.5 ml; Wholegrain bread — 51.7 g

Day 10

Breakfast. *Banana almond oat bowl* — Rolled oats — 51.7 g; Low-fat milk — 232.7 ml; Banana — 116.3 g; Almonds — 19.4 g; Honey — 12.9 g **Snack 1.** *Greek yogurt with honey* — Greek yogurt (2% fat) — 193.9 g; Honey — 12.9 g

Lunch. *Greek salad with grilled shrimp* — Shrimp, cooked — 193.9 g; Tomato — 193.9 g; Cucumber — 129.3 g; Bell pepper — 77.6 g; Feta cheese

— 64.6 g; Kalamata olives — 25.9 g; Olive oil — 19.4 ml

Snack 2. *Walnuts and dried apricots* — Walnuts — 25.9 g; Dried apricots — 32.3 g

Dinner. *Grilled shrimp with quinoa salad* — Shrimp, cooked — 206.8 g; Quinoa, cooked — 232.7 g; Cucumber — 103.4 g; Tomato — 103.4 g; Olive oil — 12.9 ml

Day 11

Breakfast. *Greek yogurt with honey and walnuts* — Greek yogurt (2% fat) — 323.2 g; Honey — 25.9 g; Walnuts — 25.9 g; Strawberries — 103.4 g **Snack 1.** *Orange with walnuts* — Orange — 193.9 g; Walnuts — 19.4 g

Lunch. *Chickpea and tomato stew with bread* — Chickpeas, cooked — 387.8 g; Tomato — 129.3 g; Olive oil — 15.5 ml; Wholegrain bread — 64.6 g **Snack 2.** *Yogurt with honey and sesame seeds* — Greek yogurt (2% fat) — 193.9 g; Honey — 12.9 g; Sesame seeds — 12.9 g

Dinner. *Turkey meatballs with tomato sauce and pasta* — Turkey breast, roasted — 193.9 g; Egg — 38.8 g; Wholegrain pasta, cooked — 258.5 g; Tomato — 155.1 g; Olive oil — 12.9 ml

Day 12

Breakfast. *Vegetable and feta omelette with wholegrain bread* — Egg — 155.1 g; Spinach — 51.7 g; Tomato — 64.6 g; Feta cheese — 38.8 g; Olive oil — 6.5 ml; Wholegrain bread — 51.7 g

Snack 1. *Wholegrain crackers with feta* — Wholegrain crackers — 38.8 g; Feta cheese — 25.9 g

Lunch. *Wholegrain pasta with tuna and tomato* — Wholegrain pasta, cooked — 323.2 g; Tuna, canned in water — 129.3 g; Tomato — 129.3 g; Olive oil — 12.9 ml

Snack 2. *Watermelon with feta cheese* — Watermelon — 258.5 g; Feta cheese — 38.8 g

Dinner. *Octopus with boiled potatoes and lemon* — Octopus, cooked — 193.9 g; Potato, boiled — 232.7 g; Olive oil — 12.9 ml; Lemon juice — 12.9 ml

Day 13

Breakfast. *Oat porridge with milk, banana and almonds* — Rolled oats — 64.6 g; Low-fat milk — 258.5 ml; Banana — 129.3 g; Almonds — 19.4 g **Snack 1.** *Grapes with almonds* — Grapes — 155.1 g; Almonds — 15.5 g

Lunch. *Baked salmon with quinoa and zucchini* — Salmon, grilled — 193.9 g; Quinoa, cooked — 258.5 g; Zucchini — 129.3 g; Olive oil — 12.9 ml **Snack 2.** *Cottage cheese with pomegranate* — Cottage cheese (low fat) — 193.9 g; Pomegranate seeds — 77.6 g

Dinner. *Lentil and vegetable stew with feta* — Lentils, cooked — 387.8 g; Carrot — 77.6 g; Tomato — 129.3 g; Feta cheese — 38.8 g; Olive oil — 12.9 ml

Day 14

Breakfast. *Wholegrain toast with hummus, tomato, cucumber and egg* — Wholegrain bread — 77.6 g; Hummus — 51.7 g; Tomato — 77.6 g; Cucumber — 77.6 g; Egg — 71.1 g

Snack 1. *Carrot sticks with hummus* — Carrot — 129.3 g; Hummus — 51.7 g

Lunch. *White bean salad with olive oil and bread* — White beans, cooked — 387.8 g; Tomato — 103.4 g; Red onion — 38.8 g; Olive oil — 19.4 ml; Wholegrain bread — 51.7 g

Snack 2. *Greek yogurt with pumpkin seeds* — Greek yogurt (2% fat) — 193.9 g; Pumpkin seeds — 19.4 g

Dinner. *Baked sardines with tomato, zucchini and bread* — Sardines, canned — 155.1 g; Tomato — 129.3 g; Zucchini — 129.3 g; Olive oil — 15.5 ml; Wholegrain bread — 64.6 g

Day 15

Breakfast. *Greek yogurt parfait with oats, honey and figs* — Greek yogurt (2% fat) — 258.5 g; Rolled oats — 38.8 g; Honey — 19.4 g; Fresh fig — 103.4 g

Snack 1. *Banana with almonds* — Banana — 129.3 g; Almonds — 15.5 g

Lunch. *Chicken souvlaki plate with pita and yogurt* — Chicken breast, grilled — 193.9 g; Wholegrain bread — 90.5 g; Tomato — 77.6 g; Cucumber — 77.6 g; Greek yogurt (2% fat) — 77.6 g; Olive oil — 10.3 ml

Snack 2. *Mixed fruit salad* — Apple — 103.4 g; Orange — 103.4 g; Grapes — 77.6 g

Dinner. *Mussels in tomato sauce with bread* — Mussels, cooked — 258.5 g; Tomato — 193.9 g; Olive oil — 15.5 ml; Wholegrain bread — 64.6 g

Day 16

Breakfast. *Feta and tomato sandwich with olives* — Wholegrain bread — 90.5 g; Feta cheese — 51.7 g; Tomato — 77.6 g; Kalamata olives — 25.9 g; Olive oil — 6.5 ml

Snack 1. *Dried apricots with walnuts* — Dried apricots — 38.8 g; Walnuts — 19.4 g

Lunch. *Octopus with white beans and spinach* — Octopus, cooked — 168 g; White beans, cooked — 258.5 g; Spinach — 77.6 g; Olive oil — 15.5 ml **Snack 2.** *Hummus with carrot and cucumber* — Hummus — 77.6 g; Carrot — 103.4 g; Cucumber — 77.6 g

Dinner. *Grilled chicken with roasted vegetables* — Chicken breast, grilled — 219.8 g; Eggplant — 129.3 g; Zucchini — 129.3 g; Potato, boiled — 129.3 g; Olive oil — 15.5 ml

Day 17

Breakfast. *Egg and roasted pepper plate with bread* — Egg — 142.2 g; Bell pepper — 77.6 g; Wholegrain bread — 51.7 g; Olive oil — 6.5 ml **Snack 1.** *Apple with almonds* — Apple — 193.9 g; Almonds — 19.4 g

Lunch. *Turkey breast with brown rice and peppers* — Turkey breast, roasted — 193.9 g; Brown rice, cooked — 258.5 g; Bell pepper — 103.4 g; Olive oil — 12.9 ml

Snack 2. *Wholegrain crackers with feta cheese* — Wholegrain crackers — 38.8 g; Feta cheese — 32.3 g

Dinner. *Baked salmon with lemon potatoes and spinach* — Salmon, grilled — 206.8 g; Potato, boiled — 258.5 g; Spinach — 77.6 g; Olive oil — 12.9 ml; Lemon juice — 12.9 ml

Day 18

Breakfast. *Fruit and walnut yogurt bowl* — Greek yogurt (2% fat) — 284.4 g; Walnuts — 19.4 g; Honey — 19.4 g; Orange — 129.3 g **Snack 1.** *Greek yogurt with honey* — Greek yogurt (2% fat) — 193.9 g; Honey — 12.9 g

Lunch. *Grilled chicken breast with brown rice and salad* — Chicken breast, grilled — 193.9 g; Brown rice, cooked — 258.5 g; Tomato — 103.4 g; Cucumber — 103.4 g; Olive oil — 12.9 ml; Lemon juice — 12.9 ml

Snack 2. *Walnuts and dried apricots* — Walnuts — 25.9 g; Dried apricots — 32.3 g

Dinner. *Stuffed peppers with rice and feta* — Bell pepper — 258.5 g; Brown rice, cooked — 193.9 g; Tomato — 129.3 g; Feta cheese — 51.7 g; Olive oil — 15.5 ml

Day 19

Breakfast. *Savoury yogurt with cucumber, garlic and bread* — Greek yogurt (2% fat) — 258.5 g; Cucumber — 103.4 g; Wholegrain bread — 64.6 g; Olive oil — 6.5 ml; Garlic — 3.9 g

Snack 1. *Orange with walnuts* — Orange — 193.9 g; Walnuts — 19.4 g

Lunch. *Lentil soup with wholegrain bread* — Lentils, cooked — 452.4 g; Carrot — 64.6 g; Olive oil — 12.9 ml; Wholegrain bread — 64.6 g **Snack 2.** *Yogurt with honey and sesame seeds* — Greek yogurt (2% fat) — 193.9 g; Honey — 12.9 g; Sesame seeds — 12.9 g

Dinner. *Chickpea and spinach stew with bread* — Chickpeas, cooked — 387.8 g; Spinach — 129.3 g; Tomato — 129.3 g; Olive oil — 15.5 ml; Wholegrain bread — 51.7 g

Day 20

Breakfast. *Banana almond oat bowl* — Rolled oats — 51.7 g; Low-fat milk — 232.7 ml; Banana — 116.3 g; Almonds — 19.4 g; Honey — 12.9 g **Snack 1.** *Wholegrain crackers with feta* — Wholegrain crackers — 38.8 g; Feta cheese — 25.9 g

Lunch. *Greek salad with grilled shrimp* — Shrimp, cooked — 193.9 g; Tomato — 193.9 g; Cucumber — 129.3 g; Bell pepper — 77.6 g; Feta cheese — 64.6 g; Kalamata olives — 25.9 g; Olive oil — 19.4 ml

Snack 2. *Watermelon with feta cheese* — Watermelon — 258.5 g; Feta cheese — 38.8 g

Dinner. *Grilled shrimp with quinoa salad* — Shrimp, cooked — 206.8 g; Quinoa, cooked — 232.7 g; Cucumber — 103.4 g; Tomato — 103.4 g; Olive oil — 12.9 ml

Day 21

Breakfast. *Greek yogurt with honey and walnuts* — Greek yogurt (2% fat) — 323.2 g; Honey — 25.9 g; Walnuts — 25.9 g; Strawberries — 103.4 g **Snack 1.** *Grapes with almonds* — Grapes — 155.1 g; Almonds — 15.5 g

Lunch. *Chickpea and tomato stew with bread* — Chickpeas, cooked — 387.8 g; Tomato — 129.3 g; Olive oil — 15.5 ml; Wholegrain bread — 64.6 g **Snack 2.** *Cottage cheese with pomegranate* — Cottage cheese (low fat) — 193.9 g; Pomegranate seeds — 77.6 g

Dinner. *Turkey meatballs with tomato sauce and pasta* — Turkey breast, roasted — 193.9 g; Egg — 38.8 g; Wholegrain pasta, cooked — 258.5 g; Tomato — 155.1 g; Olive oil — 12.9 ml

Day 22

Breakfast. *Vegetable and feta omelette with wholegrain bread* — Egg — 155.1 g; Spinach — 51.7 g; Tomato — 64.6 g; Feta cheese — 38.8 g; Olive oil — 6.5 ml; Wholegrain bread — 51.7 g

Snack 1. *Carrot sticks with hummus* — Carrot — 129.3 g; Hummus — 51.7 g

Lunch. *Wholegrain pasta with tuna and tomato* — Wholegrain pasta, cooked — 323.2 g; Tuna, canned in water — 129.3 g; Tomato — 129.3 g; Olive oil — 12.9 ml

Snack 2. *Greek yogurt with pumpkin seeds* — Greek yogurt (2% fat) — 193.9 g; Pumpkin seeds — 19.4 g

Dinner. *Octopus with boiled potatoes and lemon* — Octopus, cooked — 193.9 g; Potato, boiled — 232.7 g; Olive oil — 12.9 ml; Lemon juice — 12.9 ml

Day 23

Breakfast. *Oat porridge with milk, banana and almonds* — Rolled oats — 64.6 g; Low-fat milk — 258.5 ml; Banana — 129.3 g; Almonds — 19.4 g **Snack 1.** *Banana with almonds* — Banana — 129.3 g; Almonds — 15.5 g

Lunch. *Baked salmon with quinoa and zucchini* — Salmon, grilled — 193.9 g; Quinoa, cooked — 258.5 g; Zucchini — 129.3 g; Olive oil — 12.9 ml **Snack 2.** *Mixed fruit salad* — Apple — 103.4 g; Orange — 103.4 g; Grapes — 77.6 g

Dinner. *Lentil and vegetable stew with feta* — Lentils, cooked — 387.8 g; Carrot — 77.6 g; Tomato — 129.3 g; Feta cheese — 38.8 g; Olive oil — 12.9 ml

Day 24

Breakfast. *Wholegrain toast with hummus, tomato, cucumber and egg* — Wholegrain bread — 77.6 g; Hummus — 51.7 g; Tomato — 77.6 g; Cucumber — 77.6 g; Egg — 71.1 g

Snack 1. *Dried apricots with walnuts* — Dried apricots — 38.8 g; Walnuts — 19.4 g

Lunch. *White bean salad with olive oil and bread* — White beans, cooked — 387.8 g; Tomato — 103.4 g; Red onion — 38.8 g; Olive oil — 19.4 ml; Wholegrain bread — 51.7 g

Snack 2. *Hummus with carrot and cucumber* — Hummus — 77.6 g; Carrot — 103.4 g; Cucumber — 77.6 g

Dinner. *Baked sardines with tomato, zucchini and bread* — Sardines, canned — 155.1 g; Tomato — 129.3 g; Zucchini — 129.3 g; Olive oil — 15.5 ml; Wholegrain bread — 64.6 g

Day 25

Breakfast. *Greek yogurt parfait with oats, honey and figs* — Greek yogurt (2% fat) — 258.5 g; Rolled oats — 38.8 g; Honey — 19.4 g; Fresh fig — 103.4 g

Snack 1. *Apple with almonds* — Apple — 193.9 g; Almonds — 19.4 g

Lunch. *Chicken souvlaki plate with pita and yogurt* — Chicken breast, grilled — 193.9 g; Wholegrain bread — 90.5 g; Tomato — 77.6 g; Cucumber — 77.6 g; Greek yogurt (2% fat) — 77.6 g; Olive oil — 10.3 ml

Snack 2. *Wholegrain crackers with feta cheese* — Wholegrain crackers — 38.8 g; Feta cheese — 32.3 g

Dinner. *Mussels in tomato sauce with bread* — Mussels, cooked — 258.5 g; Tomato — 193.9 g; Olive oil — 15.5

ml; Wholegrain bread — 64.6 g

Day 26

Breakfast. *Feta and tomato sandwich with olives* — Wholegrain bread — 90.5 g; Feta cheese — 51.7 g; Tomato — 77.6 g; Kalamata olives — 25.9 g; Olive oil — 6.5 ml

Snack 1. *Greek yogurt with honey* — Greek yogurt (2% fat) — 193.9 g; Honey — 12.9 g

Lunch. *Octopus with white beans and spinach* — Octopus, cooked — 168 g; White beans, cooked — 258.5 g; Spinach — 77.6 g; Olive oil — 15.5 ml **Snack 2.** *Walnuts and dried apricots* — Walnuts — 25.9 g; Dried apricots — 32.3 g

Dinner. *Grilled chicken with roasted vegetables* — Chicken breast, grilled — 219.8 g; Eggplant — 129.3 g; Zucchini — 129.3 g; Potato, boiled — 129.3 g; Olive oil — 15.5 ml

Day 27

Breakfast. *Egg and roasted pepper plate with bread* — Egg — 142.2 g; Bell pepper — 77.6 g; Wholegrain bread — 51.7 g; Olive oil — 6.5 ml **Snack 1.** *Orange with walnuts* — Orange — 193.9 g; Walnuts — 19.4 g

Lunch. *Turkey breast with brown rice and peppers* — Turkey breast, roasted — 193.9 g; Brown rice, cooked — 258.5 g; Bell pepper — 103.4 g; Olive oil — 12.9 ml

Snack 2. *Yogurt with honey and sesame seeds* — Greek yogurt (2% fat) — 193.9 g; Honey — 12.9 g; Sesame seeds — 12.9 g

Dinner. *Baked salmon with lemon potatoes and spinach* — Salmon, grilled — 206.8 g; Potato, boiled — 258.5 g; Spinach — 77.6 g; Olive oil — 12.9 ml; Lemon juice — 12.9 ml

Day 28

Breakfast. *Fruit and walnut yogurt bowl* — Greek yogurt (2% fat) — 284.4 g; Walnuts — 19.4 g; Honey — 19.4 g; Orange — 129.3 g **Snack 1.** *Wholegrain crackers with feta* — Wholegrain crackers — 38.8 g; Feta cheese — 25.9 g

Lunch. *Grilled chicken breast with brown rice and salad* — Chicken breast, grilled — 193.9 g; Brown rice, cooked — 258.5 g; Tomato — 103.4 g; Cucumber — 103.4 g; Olive oil — 12.9 ml; Lemon juice — 12.9 ml

Snack 2. *Watermelon with feta cheese* — Watermelon — 258.5 g; Feta cheese — 38.8 g

Dinner. *Stuffed peppers with rice and feta* — Bell pepper — 258.5 g; Brown rice, cooked — 193.9 g; Tomato — 129.3 g; Feta cheese — 51.7 g; Olive oil — 15.5 ml

7.2. Comparison with the MAI-DIET plan

The language-model plan reported a mean daily energy of 2399.8 kcal (-0.0 % of target) with a standard deviation of 167.9 kcal and a daily range of 2212 to 2725 kcal. The MAI-DIET plan for the same profile reported 2420.0 kcal (+0.8 %) with a standard deviation of 53.7 kcal. The language-model plan met 11 of the thirteen reference criteria and the MAI-DIET plan met 10. Day-to-day dispersion was 3.1 times greater in the language-model plan, so that individual days departed from the daily tolerance band even though the 28-day mean sat close to the target. Sodium was 1636.5 mg/day against 2955.1 mg/day for MAI-DIET, and calcium 1070.0 mg/day against 1213.1 mg/day. Protein was 161.8 g/day against 126.6 g/day, both above the reference range for this profile. The plan contained 46 unique dishes across 140 meal slots.

7.3. Compliance heat map

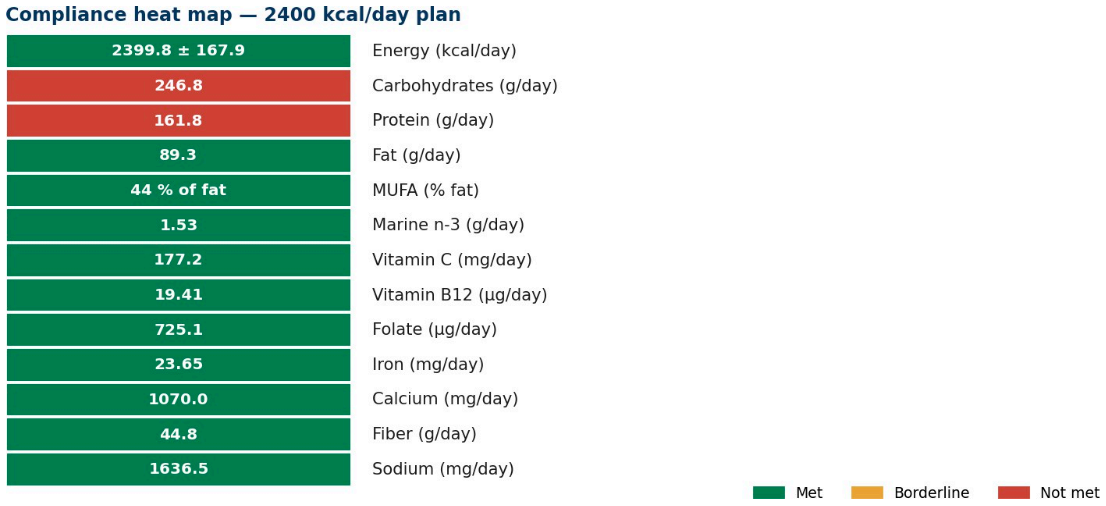

Figure S6. Compliance of the 2400 kcal/day language-model plan against the thirteen reference criteria applied in **Supplementary Table S7**. Values shown within each cell are the mean daily values reported by the model.

8. Plan 6 — 2600 kcal/day

**Sex**	Male	**Age**	30 y	**Height**	178 cm
**Body mass**	67 kg	**Activity**	1.725 (very active)	**Goal**	Weight maintenance

8.1. Twenty-eight-day meal plan

Day 1

Breakfast. *Vegetable Omelette with Whole-Wheat Toast* — Egg — 120 g; Spinach — 40 g; Tomato — 50 g; Olive oil — 8 ml; Whole-wheat bread — 160 g

Snack 1. *Greek Yogurt with Honey* — Greek yogurt (0% fat) — 170 g; Honey — 12 g

Lunch. *Grilled Salmon with Bulgur and Green Beans, Greek Salad* — Salmon fillet — 150 g; Green beans — 150 g; Tomato — 80 g; Cucumber — 60 g; Onion — 30 g; Olives — 15 g; Feta cheese — 30 g; Olive oil — 12 ml; Lemon juice — 10 ml; Bulgur (cooked) — 325 g

Snack 2. *Mixed Nuts & Dried Figs* — Cashews — 25 g; Dried figs — 25 g

Dinner. *Lentils Cooked with Green Beans and Whole Wheat Pita* — Lentils (cooked) — 140 g; Green beans — 180 g; Onion — 30 g; Garlic — 5 g; Olive oil — 12 ml; Lemon juice — 10 ml; Parsley — 5 g; Whole-wheat pita bread — 185 g

Day 2

Breakfast. *Mediterranean Toast: Feta, Tomato & Olives* — Feta cheese — 40 g; Tomato — 70 g; Olives — 15 g; Olive oil — 8 ml; Whole-wheat bread — 180 g

Snack 1. *Whole-Grain Crackers with Feta* — Whole-grain crackers — 30 g; Feta cheese — 30 g

Lunch. *Grilled Chickpeas Cooked with Whole Wheat Pasta and Broccoli, Greek Salad* — Chickpeas (cooked) — 160 g; Broccoli — 150 g; Tomato — 80 g; Cucumber — 60 g; Onion — 30 g; Olives — 15 g; Feta cheese — 30 g; Olive oil — 12 ml; Lemon juice — 10 ml; Whole-wheat pasta (cooked) — 255 g

Snack 2. *Whole-Grain Crackers with Tahini* — Whole-grain crackers — 30 g; Tahini — 15 g

Dinner. *Chicken Breast with Broccoli and Brown Rice* — Chicken breast — 130 g; Broccoli — 180 g; Onion — 30 g; Garlic — 5 g; Olive oil — 12 ml; Lemon juice — 10 ml; Parsley — 5 g; Brown rice (cooked) — 300 g

Day 3

Breakfast. *Oats Porridge with Milk, Cinnamon & Fruit* — Milk (1.5% fat) — 200 ml; Cinnamon — 2 g; Honey — 10 g; Strawberries — 100 g; Rolled oats — 130 g

Snack 1. *Rusk with Tomato & Olive Oil* — Whole-wheat rusk — 30 g; Tomato — 60 g; Olive oil — 5 ml

Lunch. *Grilled Beef Lean with Quinoa and Cauliflower, Greek Salad* — Lean beef — 150 g; Cauliflower — 150 g; Tomato — 80 g; Cucumber — 60 g; Onion — 30 g; Olives — 15 g; Feta cheese — 30 g; Olive oil — 12 ml; Lemon juice — 10 ml; Quinoa (cooked) — 275 g

Snack 2. *Fresh Fruit with Almonds* — Apricot — 150 g; Almonds — 20 g

Dinner. *Chickpeas Cooked with Cabbage and Potato* — Chickpeas (cooked) — 140 g; Cabbage — 180 g; Onion — 30 g; Garlic — 5 g; Olive oil — 12 ml; Lemon juice — 10 ml; Parsley — 5 g; Potato — 300 g

Day 4

Breakfast. *Cottage Cheese Toast with Cucumber & Tomato* — Cottage cheese — 150 g; Cucumber — 60 g; Tomato — 60 g; Olive oil — 6 ml; Whole-wheat bread — 175 g

Snack 1. *Dried Figs with Almonds* — Dried figs — 30 g; Almonds — 20 g

Lunch. *Grilled Tuna Fresh with Potato and Okra, Greek Salad* — Fresh tuna — 150 g; Okra — 150 g; Tomato — 80 g; Cucumber — 60 g; Onion — 30 g; Olives — 15 g; Feta cheese — 30 g; Olive oil — 12 ml; Lemon juice — 10 ml; Potato — 350 g

Snack 2. *Greek Yogurt with Walnuts & Honey* — Greek yogurt (2% fat) — 180 g; Pistachios — 20 g; Honey — 10 g

Dinner. *Tuna Canned Water with Artichoke and Barley* — Tuna (canned in water) — 130 g; Artichoke — 180 g; Onion — 30 g; Garlic — 5 g; Olive oil — 12 ml; Lemon juice — 10 ml; Parsley — 5 g; Barley (cooked) — 300 g

Day 5

Breakfast. *Tahini-Honey Toast with Banana* — Tahini — 20 g; Honey — 12 g; Banana — 100 g; Whole-wheat bread — 165 g **Snack 1.** *Fresh Fruit Plate* — Apricot — 120 g; Banana — 100 g

Lunch. *Grilled Lentils Cooked with Sweet Potato and Eggplant, Greek Salad* — Lentils (cooked) — 160 g; Eggplant — 150 g; Tomato — 80 g; Cucumber — 60 g; Onion — 30 g; Olives — 15 g; Feta cheese — 30 g; Olive oil — 12 ml; Lemon juice — 10 ml; Sweet potato — 350 g **Snack 2.** *Hummus with Whole-Wheat Pita* — Hummus — 80 g; Whole-wheat pita bread — 50 g

Dinner. *Shrimp with Okra and Quinoa* — Shrimp — 130 g; Okra — 180 g; Onion — 30 g; Garlic — 5 g; Olive oil — 12 ml; Lemon juice — 10 ml; Parsley — 5 g; Quinoa (cooked) — 300 g

Day 6

Breakfast. *Boiled Eggs with Whole-Wheat Bread, Olives & Cucumber* — Egg — 100 g; Olives — 20 g; Cucumber — 60 g; Olive oil — 6 ml; Whole-wheat bread — 170 g

Snack 1. *Fresh Fruit with Mixed Nuts* — Fresh fig — 150 g; Cashews — 25 g

Lunch. *Grilled Turkey Breast with Brown Rice and Carrot, Greek Salad* — Turkey breast — 150 g; Carrot — 150 g; Tomato — 80 g; Cucumber — 60 g; Onion — 30 g; Olives — 15 g; Feta cheese — 30 g; Olive oil — 12 ml; Lemon juice — 10 ml; Brown rice (cooked) — 295 g

Snack 2. *Fresh Fruit with Cottage Cheese* — Watermelon — 120 g; Cottage cheese — 100 g

Dinner. *Salmon with Cauliflower and Bulgur* — Salmon fillet — 130 g; Cauliflower — 180 g; Onion — 30 g; Garlic — 5 g; Olive oil — 12 ml; Lemon juice — 10 ml; Parsley — 5 g; Bulgur (cooked) — 300 g

Day 7

Breakfast. *Greek Yogurt Bowl with Honey, Walnuts & Fruit* — Greek yogurt (2% fat) — 220 g; Honey — 15 g; Cashews — 20 g; Peach — 100 g; Rolled oats — 80 g

Snack 1. *Greek Yogurt with Honey* — Greek yogurt (0% fat) — 170 g; Honey — 12 g

Lunch. *Grilled Shrimp with Bulgur and Cabbage, Greek Salad* — Shrimp — 150 g; Cabbage — 150 g; Tomato — 80 g; Cucumber — 60 g; Onion — 30 g; Olives — 15 g; Feta cheese — 30 g; Olive oil — 12 ml; Lemon juice — 10 ml; Bulgur (cooked) — 350 g

Snack 2. *Mixed Nuts & Dried Figs* — Almonds — 25 g; Dried figs — 25 g

Dinner. *Turkey Breast with Carrot and Whole Wheat Pita* — Turkey breast — 130 g; Carrot — 180 g; Onion — 30 g; Garlic — 5 g; Olive oil — 12 ml; Lemon juice — 10 ml; Parsley — 5 g; Whole-wheat pita bread — 170 g

Day 8

Breakfast. *Vegetable Omelette with Whole-Wheat Toast* — Egg — 120 g; Spinach — 40 g; Tomato — 50 g; Olive oil — 8 ml; Whole-wheat bread — 160 g

Snack 1. *Whole-Grain Crackers with Feta* — Whole-grain crackers — 30 g; Feta cheese — 30 g

Lunch. *Grilled Pork Tenderloin with Whole Wheat Pasta and Mushrooms, Greek Salad* — Pork tenderloin — 150 g; Mushrooms — 150 g; Tomato — 80 g; Cucumber — 60 g; Onion — 30 g; Olives — 15 g; Feta cheese — 30 g; Olive oil — 12 ml; Lemon juice — 10 ml; Whole-wheat pasta (cooked) — 310 g

Snack 2. *Whole-Grain Crackers with Tahini* — Whole-grain crackers — 30 g; Tahini — 15 g

Dinner. *Beef Lean with Spinach and Brown Rice* — Lean beef — 130 g; Spinach — 180 g; Onion — 30 g; Garlic — 5 g; Olive oil — 12 ml; Lemon juice — 10 ml; Parsley — 5 g; Brown rice (cooked) — 300 g

Day 9

Breakfast. *Mediterranean Toast: Feta, Tomato & Olives* — Feta cheese — 40 g; Tomato — 70 g; Olives — 15 g; Olive oil — 8 ml; Whole-wheat bread — 180 g

Snack 1. *Rusk with Tomato & Olive Oil* — Whole-wheat rusk — 30 g; Tomato — 60 g; Olive oil — 5 ml

Lunch. *Grilled Sardines with Quinoa and Artichoke, Greek Salad* — Sardines — 150 g; Artichoke — 150 g; Tomato

— 80 g; Cucumber — 60 g; Onion — 30 g; Olives — 15 g; Feta cheese — 30 g; Olive oil — 12 ml; Lemon juice — 10 ml; Quinoa (cooked) — 205 g

Snack 2. *Fresh Fruit with Almonds* — Orange — 150 g; Almonds — 20 g

Dinner. *Octopus with Eggplant and Potato* — Octopus — 130 g; Eggplant — 180 g; Onion — 30 g; Garlic — 5 g; Olive oil — 12 ml; Lemon juice
— 10 ml; Parsley — 5 g; Potato — 300 g

Day 10

Breakfast. *Oats Porridge with Milk, Cinnamon & Fruit* — Milk (1.5% fat) — 200 ml; Cinnamon — 2 g; Honey — 10 g; Melon — 100 g; Rolled oats
— 130 g

Snack 1. *Dried Figs with Almonds* — Dried figs — 30 g; Almonds — 20 g

Lunch. *Grilled Octopus with Potato and Zucchini, Greek Salad* — Octopus — 150 g; Zucchini — 150 g; Tomato — 80 g; Cucumber — 60 g; Onion
— 30 g; Olives — 15 g; Feta cheese — 30 g; Olive oil — 12 ml; Lemon juice — 10 ml; Potato — 350 g

Snack 2. *Greek Yogurt with Walnuts & Honey* — Greek yogurt (2% fat) — 180 g; Walnuts — 20 g; Honey — 10 g

Dinner. *Mussels with Zucchini and Barley* — Mussels — 130 g; Zucchini — 180 g; Onion — 30 g; Garlic — 5 g; Olive oil — 12 ml; Lemon juice — 10 ml; Parsley — 5 g; Barley (cooked) — 300 g

Day 11

Breakfast. *Cottage Cheese Toast with Cucumber & Tomato* — Cottage cheese — 150 g; Cucumber — 60 g; Tomato — 60 g; Olive oil — 6 ml; Whole-wheat bread — 175 g

Snack 1. *Fresh Fruit Plate* — Orange — 120 g; Kiwi — 100 g

Lunch. *Grilled Chicken Breast with Sweet Potato and Green Beans, Greek Salad* — Chicken breast — 150 g; Green beans — 150 g; Tomato — 80 g; Cucumber — 60 g; Onion — 30 g; Olives — 15 g; Feta cheese — 30 g; Olive oil — 12 ml; Lemon juice — 10 ml; Sweet potato — 350 g

Snack 2. *Hummus with Whole-Wheat Pita* — Hummus — 80 g; Whole-wheat pita bread — 50 g

Dinner. *Chickpeas Cooked with Green Beans and Quinoa* — Chickpeas (cooked) — 140 g; Green beans — 180 g; Onion — 30 g; Garlic — 5 g; Olive oil — 12 ml; Lemon juice — 10 ml; Parsley — 5 g; Quinoa (cooked) — 300 g

Day 12

Breakfast. *Tahini-Honey Toast with Banana* — Tahini — 20 g; Honey — 12 g; Banana — 100 g; Whole-wheat bread — 165 g **Snack 1.** *Fresh Fruit with Mixed Nuts* — Strawberries — 150 g; Almonds — 25 g

Lunch. *Grilled Chickpeas Cooked with Brown Rice and Broccoli, Greek Salad* — Chickpeas (cooked) — 160 g; Broccoli — 150 g; Tomato — 80 g; Cucumber — 60 g; Onion — 30 g; Olives — 15 g; Feta cheese — 30 g; Olive oil — 12 ml; Lemon juice — 10 ml; Brown rice (cooked) — 255 g **Snack 2.** *Fresh Fruit with Cottage Cheese* — Pear — 120 g; Cottage cheese — 100 g

Dinner. *Chicken Breast with Broccoli and Bulgur* — Chicken breast — 130 g; Broccoli — 180 g; Onion — 30 g; Garlic — 5 g; Olive oil — 12 ml; Lemon juice — 10 ml; Parsley — 5 g; Bulgur (cooked) — 300 g

Day 13

Breakfast. *Boiled Eggs with Whole-Wheat Bread, Olives & Cucumber* — Egg — 100 g; Olives — 20 g; Cucumber — 60 g; Olive oil — 6 ml; Whole-wheat bread — 170 g

Snack 1. *Greek Yogurt with Honey* — Greek yogurt (0% fat) — 170 g; Honey — 12 g

Lunch. *Grilled Salmon with Bulgur and Cauliflower, Greek Salad* — Salmon fillet — 150 g; Cauliflower — 150 g; Tomato — 80 g; Cucumber — 60 g; Onion — 30 g; Olives — 15 g; Feta cheese — 30 g; Olive oil — 12 ml; Lemon juice — 10 ml; Bulgur (cooked) — 340 g

Snack 2. *Mixed Nuts & Dried Figs* — Cashews — 25 g; Dried figs — 25 g

Dinner. *Lentils Cooked with Cabbage and Whole Wheat Pita* — Lentils (cooked) — 140 g; Cabbage — 180 g;

Onion — 30 g; Garlic — 5 g; Olive oil
— 12 ml; Lemon juice — 10 ml; Parsley — 5 g; Whole-wheat pita bread — 190 g

Day 14

Breakfast. *Greek Yogurt Bowl with Honey, Walnuts & Fruit* — Greek yogurt (2% fat) — 220 g; Honey — 15 g; Pistachios — 20 g; Orange — 100 g; Rolled oats — 75 g

Snack 1. *Whole-Grain Crackers with Feta* — Whole-grain crackers — 30 g; Feta cheese — 30 g

Lunch. *Grilled Turkey Breast with Whole Wheat Pasta and Okra, Greek Salad* — Turkey breast — 150 g; Okra — 150 g; Tomato — 80 g; Cucumber
— 60 g; Onion — 30 g; Olives — 15 g; Feta cheese — 30 g; Olive oil — 12 ml; Lemon juice — 10 ml;
Whole-wheat pasta (cooked) — 305 g **Snack 2.** *Whole-Grain Crackers with Tahini* — Whole-grain crackers — 30 g; Tahini — 15 g

Dinner. *Anchovies with Artichoke and Brown Rice* — Anchovies — 130 g; Artichoke — 180 g; Onion — 30 g; Garlic — 5 g; Olive oil — 12 ml; Lemon juice — 10 ml; Parsley — 5 g; Brown rice (cooked) — 300 g

Day 15

Breakfast. *Vegetable Omelette with Whole-Wheat Toast* — Egg — 120 g; Spinach — 40 g; Tomato — 50 g; Olive oil — 8 ml; Whole-wheat bread — 160 g

Snack 1. *Rusk with Tomato & Olive Oil* — Whole-wheat rusk — 30 g; Tomato — 60 g; Olive oil — 5 ml

Lunch. *Grilled Lentils Cooked with Quinoa and Eggplant, Greek Salad* — Lentils (cooked) — 160 g; Eggplant — 150 g; Tomato — 80 g; Cucumber
— 60 g; Onion — 30 g; Olives — 15 g; Feta cheese — 30 g; Olive oil — 12 ml; Lemon juice — 10
ml; Quinoa (cooked) — 340 g **Snack 2.** *Fresh Fruit with Almonds* — Apricot — 150 g; Almonds
— 20 g

Dinner. *Turkey Breast with Okra and Potato* — Turkey breast — 130 g; Okra — 180 g; Onion — 30 g; Garlic — 5 g; Olive oil — 12 ml; Lemon juice — 10 ml; Parsley — 5 g; Potato — 300 g

Day 16

Breakfast. *Mediterranean Toast: Feta, Tomato & Olives* — Feta cheese — 40 g; Tomato — 70 g; Olives — 15 g; Olive oil — 8 ml; Whole-wheat bread — 180 g

Snack 1. *Dried Figs with Almonds* — Dried figs — 30 g; Almonds — 20 g

Lunch. *Grilled Beef Lean with Potato and Carrot, Greek Salad* — Lean beef — 150 g; Carrot — 150 g; Tomato — 80 g; Cucumber — 60 g; Onion
— 30 g; Olives — 15 g; Feta cheese — 30 g; Olive oil — 12 ml; Lemon juice — 10 ml; Potato — 350 g

Snack 2. *Greek Yogurt with Walnuts & Honey* — Greek yogurt (2% fat) — 180 g; Pistachios — 20 g; Honey — 10 g

Dinner. *Tuna Fresh with Cauliflower and Barley* — Fresh tuna — 130 g; Cauliflower — 180 g; Onion — 30 g; Garlic — 5 g; Olive oil — 12 ml; Lemon juice — 10 ml; Parsley — 5 g; Barley (cooked) — 300 g

Day 17

Breakfast. *Oats Porridge with Milk, Cinnamon & Fruit* — Milk (1.5% fat) — 200 ml; Cinnamon — 2 g; Honey — 10 g; Pear — 100 g; Rolled oats
— 120 g

Snack 1. *Fresh Fruit Plate* — Apricot — 120 g; Banana — 100 g

Lunch. *Grilled Tuna Fresh with Sweet Potato and Cabbage, Greek Salad* — Fresh tuna — 150 g; Cabbage — 150 g; Tomato — 80 g; Cucumber — 60 g; Onion — 30 g; Olives — 15 g; Feta cheese — 30 g; Olive oil — 12 ml; Lemon juice — 10 ml; Sweet potato — 350 g

Snack 2. *Hummus with Whole-Wheat Pita* — Hummus — 80 g; Whole-wheat pita bread — 50 g

Dinner. *Pork Tenderloin with Carrot and Quinoa* — Pork tenderloin — 130 g; Carrot — 180 g; Onion — 30 g; Garlic — 5 g; Olive oil — 12 ml; Lemon juice — 10 ml; Parsley — 5 g; Quinoa (cooked) — 300 g

Day 18

Breakfast. *Cottage Cheese Toast with Cucumber & Tomato* — Cottage cheese — 150 g; Cucumber — 60 g;

Tomato — 60 g; Olive oil — 6 ml; Whole-wheat bread — 175 g

Snack 1. *Fresh Fruit with Mixed Nuts* — Fresh fig — 150 g; Cashews — 25 g

Lunch. *Grilled Chicken Breast with Brown Rice and Mushrooms, Greek Salad* — Chicken breast — 150 g; Mushrooms — 150 g; Tomato — 80 g; Cucumber — 60 g; Onion — 30 g; Olives — 15 g; Feta cheese — 30 g; Olive oil — 12 ml; Lemon juice — 10 ml; Brown rice (cooked) — 340 g **Snack 2.** *Fresh Fruit with Cottage Cheese* — Watermelon — 120 g; Cottage cheese — 100 g

Dinner. *Chickpeas Cooked with Spinach and Bulgur* — Chickpeas (cooked) — 140 g; Spinach — 180 g; Onion — 30 g; Garlic — 5 g; Olive oil — 12 ml; Lemon juice — 10 ml; Parsley — 5 g; Bulgur (cooked) — 300 g

Day 19

Breakfast. *Tahini-Honey Toast with Banana* — Tahini — 20 g; Honey — 12 g; Banana — 100 g; Whole-wheat bread — 165 g **Snack 1.** *Greek Yogurt with Honey* — Greek yogurt (0% fat) — 170 g; Honey — 12 g

Lunch. *Grilled Shrimp with Bulgur and Artichoke, Greek Salad* — Shrimp — 150 g; Artichoke — 150 g; Tomato — 80 g; Cucumber — 60 g; Onion — 30 g; Olives — 15 g; Feta cheese — 30 g; Olive oil — 12 ml; Lemon juice — 10 ml; Bulgur (cooked) — 350 g **Snack 2.** *Mixed Nuts & Dried Figs* — Almonds — 25 g; Dried figs — 25 g

Dinner. *Salmon with Eggplant and Whole Wheat Pita* — Salmon fillet — 130 g; Eggplant — 180 g; Onion — 30 g; Garlic — 5 g; Olive oil — 12 ml; Lemon juice — 10 ml; Parsley — 5 g; Whole-wheat pita bread — 145 g

Day 20

Breakfast. *Boiled Eggs with Whole-Wheat Bread, Olives & Cucumber* — Egg — 100 g; Olives — 20 g; Cucumber — 60 g; Olive oil — 6 ml; Whole-wheat bread — 170 g

Snack 1. *Whole-Grain Crackers with Feta* — Whole-grain crackers — 30 g; Feta cheese — 30 g

Lunch. *Grilled Mussels with Whole Wheat Pasta and Zucchini, Greek Salad* — Mussels — 150 g; Zucchini — 150 g; Tomato — 80 g; Cucumber — 60 g; Onion — 30 g; Olives — 15 g; Feta cheese — 30 g; Olive oil — 12 ml; Lemon juice — 10 ml; Whole-wheat pasta (cooked) — 350 g

Snack 2. *Whole-Grain Crackers with Tahini* — Whole-grain crackers — 30 g; Tahini — 15 g

Dinner. *Chicken Breast with Zucchini and Brown Rice* — Chicken breast — 130 g; Zucchini — 180 g; Onion — 30 g; Garlic — 5 g; Olive oil — 12 ml; Lemon juice — 10 ml; Parsley — 5 g; Brown rice (cooked) — 300 g

Day 21

Breakfast. *Greek Yogurt Bowl with Honey, Walnuts & Fruit* — Greek yogurt (2% fat) — 220 g; Honey — 15 g; Almonds — 20 g; Fresh fig — 100 g; Rolled oats — 65 g

Snack 1. *Rusk with Tomato & Olive Oil* — Whole-wheat rusk — 30 g; Tomato — 60 g; Olive oil — 5 ml

Lunch. *Grilled Chickpeas Cooked with Quinoa and Green Beans, Greek Salad* — Chickpeas (cooked) — 160 g; Green beans — 150 g; Tomato — 80 g; Cucumber — 60 g; Onion — 30 g; Olives — 15 g; Feta cheese — 30 g; Olive oil — 12 ml; Lemon juice — 10 ml; Quinoa (cooked) — 265 g **Snack 2.** *Fresh Fruit with Almonds* — Orange — 150 g; Almonds — 20 g

Dinner. *Lentils Cooked with Green Beans and Potato* — Lentils (cooked) — 140 g; Green beans — 180 g; Onion — 30 g; Garlic — 5 g; Olive oil — 12 ml; Lemon juice — 10 ml; Parsley — 5 g; Potato — 300 g

Day 22

Breakfast. *Vegetable Omelette with Whole-Wheat Toast* — Egg — 120 g; Spinach — 40 g; Tomato — 50 g; Olive oil — 8 ml; Whole-wheat bread — 160 g

Snack 1. *Dried Figs with Almonds* — Dried figs — 30 g; Almonds — 20 g

Lunch. *Grilled Turkey Breast with Potato and Broccoli, Greek Salad* — Turkey breast — 150 g; Broccoli — 150 g; Tomato — 80 g; Cucumber — 60 g; Onion — 30 g; Olives — 15 g; Feta cheese — 30 g; Olive oil — 12 ml; Lemon juice — 10 ml; Potato — 350 g

Snack 2. *Greek Yogurt with Walnuts & Honey* — Greek yogurt (2% fat) — 180 g; Walnuts — 20 g; Honey — 10

g

Dinner. *Shrimp with Broccoli and Barley* — Shrimp — 130 g; Broccoli — 180 g; Onion — 30 g; Garlic — 5 g; Olive oil — 12 ml; Lemon juice — 10 ml; Parsley — 5 g; Barley (cooked) — 300 g

Day 23

Breakfast. *Mediterranean Toast: Feta, Tomato & Olives* — Feta cheese — 40 g; Tomato — 70 g; Olives — 15 g; Olive oil — 8 ml; Whole-wheat bread — 180 g

Snack 1. *Fresh Fruit Plate* — Orange — 120 g; Kiwi — 100 g

Lunch. *Grilled Salmon with Sweet Potato and Cauliflower, Greek Salad* — Salmon fillet — 150 g; Cauliflower — 150 g; Tomato — 80 g; Cucumber — 60 g; Onion — 30 g; Olives — 15 g; Feta cheese — 30 g; Olive oil — 12 ml; Lemon juice — 10 ml; Sweet potato — 325 g **Snack 2.** *Hummus with Whole-Wheat Pita* — Hummus — 80 g; Whole-wheat pita bread — 50 g

Dinner. *Sardines with Cabbage and Quinoa* — Sardines — 130 g; Cabbage — 180 g; Onion — 30 g; Garlic — 5 g; Olive oil — 12 ml; Lemon juice — 10 ml; Parsley — 5 g; Quinoa (cooked) — 300 g

Day 24

Breakfast. *Oats Porridge with Milk, Cinnamon & Fruit* — Milk (1.5% fat) — 200 ml; Cinnamon — 2 g; Honey — 10 g; Banana — 100 g; Rolled oats — 115 g

Snack 1. *Fresh Fruit with Mixed Nuts* — Strawberries — 150 g; Almonds — 25 g

Lunch. *Grilled Pork Tenderloin with Brown Rice and Okra, Greek Salad* — Pork tenderloin — 150 g; Okra — 150 g; Tomato — 80 g; Cucumber — 60 g; Onion — 30 g; Olives — 15 g; Feta cheese — 30 g; Olive oil — 12 ml; Lemon juice — 10 ml; Brown rice (cooked) — 295 g

Snack 2. *Fresh Fruit with Cottage Cheese* — Pear — 120 g; Cottage cheese — 100 g

Dinner. *Turkey Breast with Artichoke and Bulgur* — Turkey breast — 130 g; Artichoke — 180 g; Onion — 30 g; Garlic — 5 g; Olive oil — 12 ml; Lemon juice — 10 ml; Parsley — 5 g; Bulgur (cooked) — 300 g

Day 25

Breakfast. *Cottage Cheese Toast with Cucumber & Tomato* — Cottage cheese — 150 g; Cucumber — 60 g; Tomato — 60 g; Olive oil — 6 ml; Whole-wheat bread — 175 g

Snack 1. *Greek Yogurt with Honey* — Greek yogurt (0% fat) — 170 g; Honey — 12 g

Lunch. *Grilled Lentils Cooked with Bulgur and Eggplant, Greek Salad* — Lentils (cooked) — 160 g; Eggplant — 150 g; Tomato — 80 g; Cucumber — 60 g; Onion — 30 g; Olives — 15 g; Feta cheese — 30 g; Olive oil — 12 ml; Lemon juice — 10 ml; Bulgur (cooked) — 350 g **Snack 2.** *Mixed Nuts & Dried Figs* — Cashews — 25 g; Dried figs — 25 g

Dinner. *Chickpeas Cooked with Okra and Whole Wheat Pita* — Chickpeas (cooked) — 140 g; Okra — 180 g; Onion — 30 g; Garlic — 5 g; Olive oil — 12 ml; Lemon juice — 10 ml; Parsley — 5 g; Whole-wheat pita bread — 155 g

Day 26

Breakfast. *Tahini-Honey Toast with Banana* — Tahini — 20 g; Honey — 12 g; Banana — 100 g; Whole-wheat bread — 165 g **Snack 1.** *Whole-Grain Crackers with Feta* — Whole-grain crackers — 30 g; Feta cheese — 30 g

Lunch. *Grilled Chicken Breast with Whole Wheat Pasta and Carrot, Greek Salad* — Chicken breast — 150 g; Carrot — 150 g; Tomato — 80 g; Cucumber — 60 g; Onion — 30 g; Olives — 15 g; Feta cheese — 30 g; Olive oil — 12 ml; Lemon juice — 10 ml; Whole-wheat pasta (cooked) — 315 g

Snack 2. *Whole-Grain Crackers with Tahini* — Whole-grain crackers — 30 g; Tahini — 15 g

Dinner. *Tuna Canned Water with Cauliflower and Brown Rice* — Tuna (canned in water) — 130 g; Cauliflower — 180 g; Onion — 30 g; Garlic — 5 g; Olive oil — 12 ml; Lemon juice — 10 ml; Parsley — 5 g; Brown rice (cooked) — 300 g

Day 27

Breakfast. *Boiled Eggs with Whole-Wheat Bread, Olives & Cucumber* — Egg — 100 g; Olives — 20 g; Cucumber — 60 g; Olive oil — 6 ml; Whole-wheat bread — 170 g

Snack 1. *Rusk with Tomato & Olive Oil* — Whole-wheat rusk — 30 g; Tomato — 60 g; Olive oil — 5 ml

Lunch. *Grilled Sardines with Quinoa and Cabbage, Greek Salad* — Sardines — 150 g; Cabbage — 150 g; Tomato — 80 g; Cucumber — 60 g; Onion — 30 g; Olives — 15 g; Feta cheese — 30 g; Olive oil — 12 ml; Lemon juice — 10 ml; Quinoa (cooked) — 235 g

Snack 2. *Fresh Fruit with Almonds* — Apricot — 150 g; Almonds — 20 g

Dinner. *Chicken Breast with Carrot and Potato* — Chicken breast — 130 g; Carrot — 180 g; Onion — 30 g; Garlic — 5 g; Olive oil — 12 ml; Lemon juice — 10 ml; Parsley — 5 g; Potato — 300 g

Day 28

Breakfast. *Greek Yogurt Bowl with Honey, Walnuts & Fruit* — Greek yogurt (2% fat) — 220 g; Honey — 15 g; Walnuts — 20 g; Grapes — 100 g; Rolled oats — 65 g

Snack 1. *Dried Figs with Almonds* — Dried figs — 30 g; Almonds — 20 g

Lunch. *Grilled Chicken Breast with Potato and Mushrooms, Greek Salad* — Chicken breast — 150 g; Mushrooms — 150 g; Tomato — 80 g; Cucumber — 60 g; Onion — 30 g; Olives — 15 g; Feta cheese — 30 g; Olive oil — 12 ml; Lemon juice — 10 ml; Potato — 350 g

Snack 2. *Greek Yogurt with Walnuts & Honey* — Greek yogurt (2% fat) — 180 g; Pistachios — 20 g; Honey — 10 g

Dinner. *Sardines with Spinach and Barley* — Sardines — 130 g; Spinach — 180 g; Onion — 30 g; Garlic — 5 g; Olive oil — 12 ml; Lemon juice — 10 ml; Parsley — 5 g; Barley (cooked) — 300 g

8.2. Comparison with the MAI-DIET plan

The language-model plan reported a mean daily energy of 2574.1 kcal (-1.0 % of target) with a standard deviation of 90.0 kcal and a daily range of 2390 to 2722 kcal. The MAI-DIET plan for the same profile reported 2632.0 kcal (+1.2 %) with a standard deviation of 62.3 kcal. The language-model plan met 11 of the thirteen reference criteria and the MAI-DIET plan met 10. Day-to-day dispersion was 1.4 times greater in the language-model plan, so that individual days departed from the daily tolerance band even though the 28-day mean sat close to the target. Sodium was 2199.3 mg/day against 3135.8 mg/day for MAI-DIET, and calcium 1086.2 mg/day against 1402.9 mg/day. Protein was 136.2 g/day against 139.2 g/day, both above the reference range for this profile. The plan contained 75 unique dishes across 140 meal slots.

8.3. Compliance heat map

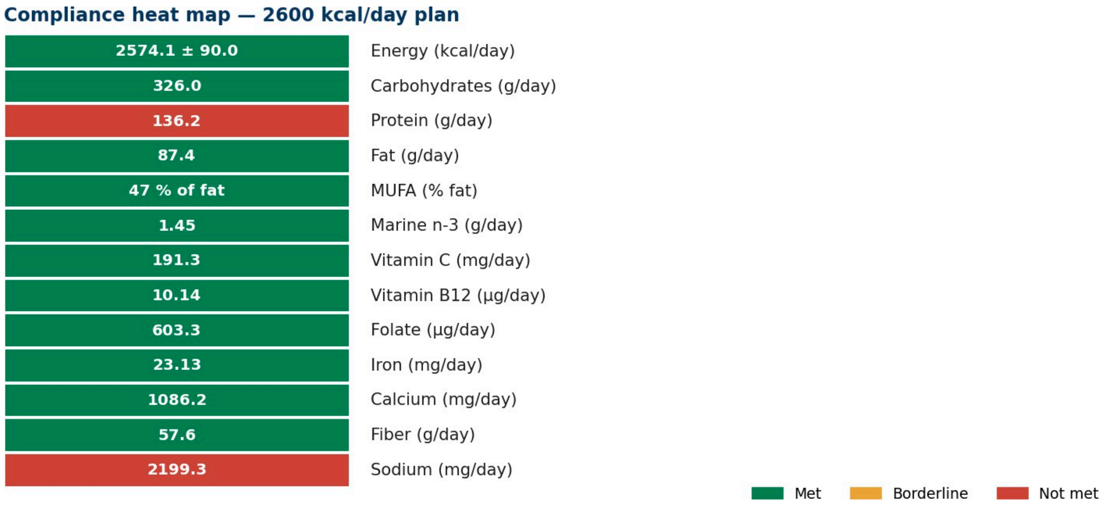

Figure S7. Compliance of the 2600 kcal/day language-model plan against the thirteen reference criteria applied in **Supplementary Table S7**. Values shown within each cell are the mean daily values reported by the model.

Error Analysis of the Six Matched Plan Set

Every 28-day plan produced by direct large-language-model generation was compared with the MAI-DIET plan generated for the same profile. Each system contributed six plans, 168 days and 840 core meal slots, counting the five requested eating occasions per day. Eight error classes were measured. The counts below are taken from the plan documents and workbooks themselves; nothing is carried over from the manuscript text.

Table S2. Eight error classes measured in the six directly generated plans and in the six MAI-DIET plans produced for the same profiles.

Error class	Direct LLM generation (six plans)	MAI-DIET (six plans)	Assessment
Days repeating an earlier day of the same plan in all five eating occasions	21 of 168 days (0, 7, 14, 0, 0, 0 per plan); in one plan days 15–28 repeat days 1–14 ingredient for ingredient	0 of 168 days	Separates the two systems. Constraints C1, C2 and C9 of the planning module apply: a recipe cannot recur within a day, cannot exceed two uses per calendar week, and cannot occupy the same slot on consecutive days. No rule tests whole-day identity directly, so the safeguard is strong but not absolute; no duplicate day occurred in 168 days.
Maximum uses of any one dish	3 to 27 over 28 days; 1 to 7 within one calendar week	6 to 7 over 28 days; 2 within one calendar week in all six plans	Separates on the bound rather than the average: the comparator is lower than MAI-DIET in five of six plans and far higher in the sixth, where one dish fills 27 of 140 core slots. Constraint C2 is a hard cap of two uses per recipe per calendar week, applied before scoring, and nothing can exceed it.
Single ingredient quantities above 300 g, excluding beverages and liquid milk	53 of 3,497 ingredient lines	28 of 3,059 ingredient lines	Does not separate the two systems. MAI-DIET's 2600 kcal plan contains 24 such lines, more than any single comparator plan. The largest quantities on both sides are cooked legumes and water-rich vegetables.
Printed ingredient quantities that are not whole numbers	514 of 3,497 (14.7 %); whole grams in five plans, one-decimal values throughout the sixth	646 of 3,059 (21.1 %); non-integer quantities in every plan	MAI-DIET is the less consistent of the two. Every recipe quantity is multiplied by k = daily target / 2000 and rounded to three decimals, which is what holds daily energy near the target; the fractional grams are its by-

			product.
Days containing all five requested eating occasions	168 of 168 days complete	168 of 168 days complete, with optional side or dessert slots additionally scheduled on 7 to 21 days per plan	No contrast.
Meal slots whose dish name is a generic placeholder rather than a named dish	88 of 840 slots, across 21 distinct placeholder names	23 of 840 slots, all one name	Separates the two systems. Dish names are fields of the curated recipe library and are not generated. In both systems the flagged slots still carry explicit ingredient lines; the defect is in the name a clinician or patient reads.
Days whose stated energy departs from 4 × protein + 4 × carbohydrate + 9 × fat by more than 5 %	0 of 168 days; mean deviation 1.54 % to 2.39 % per plan	0 of 168 days; mean deviation 0.85 % to 1.14 % per plan	No failures in either system.
Day-to-day variation in energy	SD 23.0 to 183.9 kcal; the widest plan spans 721 kcal between its lightest and heaviest day; 28-day mean within 1.00 % of the requested target	SD 33.9 to 62.3 kcal; the widest plan spans 233 kcal; 28-day mean within 1.73 % of the requested target	The comparator is closer on the 28-day mean; MAI-DIET is tighter on the individual day. Each completed MAI-DIET day is re-checked against a fixed tolerance band and a non-anchor meal is exchanged when the day falls outside it.

SD: standard deviation. C1, C2 and C9 are the constraint identifiers used in the MAI-DIET planning module and are specified in Supplementary Table S3. Each system produced 6 × 28 × 5 = 840 core meal slots

Provenance of the reported nutrient values

Three checks were run on the nutrient values reported for the directly generated plans. The mean stated in each workbook equals the mean of its 28 daily values, the largest discrepancy in any nutrient in any workbook being 0.39 kcal. No day fails the 5 % test against the Atwater factors. In the 21 days that repeat an earlier day of the same plan ingredient for ingredient, all thirteen nutrients are identical to full stated precision, including the seven scattered, interleaved pairs of one plan. The reported values therefore behave as a deterministic function of the food listed. They were not independently recomputed here against a food-composition database, so their accuracy is not established, and no claim is made about how they were derived. A fourth check, the correlation between total listed food mass and reported daily energy, discriminated nothing and no weight is placed on it: run as a control on the MAI-DIET plans, whose values are computed from the database, it gives a weaker association (r = −0.12 to +0.15) than the comparator plans (r = −0.69 to +0.4)

9. Interpretation

Both approaches produced complete 28-day plans with explicit ingredient quantities, and both achieved broadly comparable counts of met reference criteria. The plans differ in the property that the deterministic architecture was designed to provide. In the MAI-DIET plans, every reported value is the sum of ingredient-level quantities drawn from a standardized food-composition library, and the daily energy dispersion reflects the composition of the selected recipes. In the language-model plans, mean energy matched the requested target in every profile to within one per cent while daily dispersion varied more than sevenfold between profiles.

This variability is the behavior that a deterministic generation path is intended to remove, and it is observable without recomputing any nutrient value.

The MAI-DIET engine gives a useful reference point for what bounded variation looks like. When its random seed was changed across five sequences, mean daily energy varied by at most 3.2 % and any macronutrient share by at most 4.4 %, with the most seed-sensitive micronutrients varying by up to approximately 20 %. That variation comes from a single documented source and stays within known limits. The variation between the language-model plans was of a different kind: day-to-day energy dispersion differed roughly eightfold across the six profiles. In the engine, what changes between runs is which recipes are drawn; in the language-model plans, what changed between runs was how closely the stated requirements were followed.

Two limitations should be noted. Nutrient values for the language-model plans are self-reported and have not been independently recomputed from the stated ingredient quantities, so their accuracy is not established here. In addition, a single generation run was performed per profile, so run-to-run variability within the same profile was not assessed.

Supplementary Section S1. The Comparator Prompt as Issued

The prompt below is the one issued to the model, reproduced in full and without editing. It was issued once per profile. The six persona blocks follow; each was substituted for the line [ONE PERSONA BLOCK HERE] and nothing else in the prompt changed between profiles. The persona blocks match the profile table of each generated plan document field for field.

Shared prompt body, verbatim

Create a 28-day Mediterranean-style meal plan for the person described below.

Create and give me two downloadable files: a .docx file and an .xlsx file, as specified below. Name them Persona[N]_plan.docx and Persona[N]_nutrients.xlsx.

PERSON

[ONE PERSONA BLOCK HERE]

PLAN REQUIREMENTS

- Duration: 28 consecutive days.

- Five eating occasions per day: Breakfast, Lunch, Dinner, Snack 1, Snack 2.

- Mediterranean-style. Greek-Mediterranean dishes are welcome.

- Alcohol-free. No food allergies.

- Every ingredient must carry an explicit quantity in grams or millilitres. Do not write “1 medium”, “a handful”, “to taste”, or “1 cup”. Convert everything to g or ml.

OUTPUT 1 — WORD DOCUMENT (.docx)

Four sections, in this order.

Section 1. Metadata table. Two columns, "Field" and "Value". Rows: Model name; Model version; Provider; Interface; Date of generation; Run number; Tools used during generation. Leave every value as the literal text TO BE COMPLETED.

Section 2. Profile table. Two columns, "Field" and "Value". Echo back every value you were given: persona ID, target kcal/day, sex, age, height, body mass, physical activity level, goal, duration, meals per day, snacks per day, allergies, alcohol.

Section 3. The 28-day meal plan. For each day in order, a heading "Day N", then the five eating occasions in fixed order. For each occasion give the dish name, then its ingredients, each with a quantity in g or ml. Include no nutrient values in this section.

Section 4. Mean daily nutrient summary. Exactly 13 rows, one per nutrient, in this fixed order: Energy (kcal/day); Carbohydrates (g/day); Protein (g/day); Fat (g/day); MUFA (% of total fat); Marine n-3 EPA+DHA (g/day); Vitamin C (mg/day); Vitamin B12 (μg/day); Folate (μg/day); Iron (mg/day); Calcium (mg/day); Fiber (g/day); Sodium (mg/day). Three columns: "Nutrient", "Unit", "Mean daily value". Report the 28-day mean for each. Do not add any assessment, rating, or commentary on whether values are adequate.

OUTPUT 2 — EXCEL WORKBOOK (.xlsx)

Two sheets.

Sheet "Daily": header row plus 28 rows, one per day. First column "Day" (1 to 28), then one column per nutrient in the same fixed order as Section 4, with units in the header. Every cell a bare number.

Sheet "Summary": 13 rows, one per nutrient in the same fixed order. Columns: "Nutrient", "Unit", "Mean_daily", "SD".

FORMATTING RULES

- Numeric cells contain a number only. No units in the cell, no ranges, no "approx", no "~".

- Decimal point, not comma.

- Unavailable values written as NR.

- No merged cells, no blank rows, no footnotes inside tables.

- Produce all 28 days. Do not abbreviate with "repeat week 1" or similar. If you cannot complete all 28 days in one response, produce as much as you can and state clearly at the end which day you reached.

The six persona blocks, verbatim

Persona 1 | Female | 35 years | 155 cm | 50 kg | Lightly active (PAL 1.375) | Goal: weight maintenance | Target energy: about 1600 kcal/day | 28 days | 3 meals + 2 snacks | No allergies | Alcohol-free

Persona 2 | Female | 35 years | 163 cm | 65 kg | Moderately active (PAL 1.55) | Goal: mild weight loss | Target energy: about 1800 kcal/day | 28 days | 3 meals + 2 snacks | No allergies | Alcohol-free

Persona 3 | Male | 35 years | 170 cm | 72 kg | Moderately active (PAL 1.55) | Goal: mild weight loss | Target energy: about 2000 kcal/day | 28 days | 3 meals + 2 snacks | No allergies | Alcohol-free

Persona 4 | Female | 35 years | 168 cm | 75 kg | Moderately active (PAL 1.55) | Goal: weight maintenance | Target energy: about 2200 kcal/day | 28 days | 3 meals + 2 snacks | No allergies | Alcohol-free

Persona 5 | Female | 28 years | 165 cm | 64 kg | Very active (PAL 1.725) | Goal: weight maintenance | Target energy: about 2400 kcal/day | 28 days | 3 meals + 2 snacks | No allergies | Alcohol-free

Persona 6 | Male | 30 years | 178 cm | 67 kg | Very active (PAL 1.725) | Goal: weight maintenance | Target energy: about 2600 kcal/day | 28 days | 3 meals + 2 snacks | No allergies | Alcohol-free

Generation conditions

Table S3. Conditions under which the comparator plans were generated.

Item	Value
Model	Claude Sonnet 5 (Anthropic, San Francisco, CA, USA), medium effort setting
Interface	the provider's standard interactive interface, not a programmatic interface
Sampling parameters	not user-configurable through that interface and therefore not set (temperature, top-p, top-k, maximum tokens)
Runs	one prompt and one generation run per profile; the same one-run-per-profile rule was applied to MAI-DIET, so that no output in either arm was generated more than once or selected from among alternatives
Date of generation	03/08/2026
Whether each session was fresh	Confirmed

What the prompt did not supply

No nutrient reference values, no tolerance bands and no compliance criteria were supplied to the model. The prompt names thirteen nutrients only to fix the reporting format of Section 4 and of the workbook; it attaches no target, no reference range and no acceptability threshold to any of them, and it instructs the model not to comment on whether values are adequate. Compliance was assessed afterwards, by applying to both sets of plans the same thirteen reference criteria used for the MAI-DIET plans in **Supplementary Table S7**. The prompt contains no instruction on recipe repetition or dietary variety. The one sentence in it that mentions repeating a week is a formatting rule about output completeness and sets no limit on how often a dish may recur within a plan. The blank metadata tables in the six generated plan documents are not an oversight: the prompt instructs the model to leave every value as the literal text TO BE COMPLETED, and they were not filled in afterwards.
